# Proteomics and antivenomics of *Echis carinatus carinatus* venom: Correlation with pharmacological properties and pathophysiology of envenomation

**DOI:** 10.1038/s41598-017-17227-y

**Published:** 2017-12-07

**Authors:** Aparup Patra, Bhargab Kalita, Abhishek Chanda, Ashis K. Mukherjee

**Affiliations:** Microbial Biotechnology and Protein Research Laboratory, Department of Molecular Biology and Biotechnology, Tezpur University, Tezpur, 784028 Assam India

## Abstract

The proteome composition of *Echis carinatus carinatus* venom (ECV) from India was studied for the first time by tandem mass spectrometry analysis. A total of 90, 47, and 22 distinct enzymatic and non-enzymatic proteins belonging to 15, 10, and 6 snake venom protein families were identified in ECV by searching the ESI-LC-MS/MS data against non-redundant protein databases of Viperidae (taxid 8689), *Echis* (taxid 8699) and *Echis carinatus* (taxid 40353), respectively. However, analysis of MS/MS data against the Transcriptome Shotgun Assembly sequences (87 entries) of conger *E*. *coloratus* identified only 14 proteins in ECV. Snake venom metalloproteases and snaclecs, the most abundant enzymatic and non-enzymatic proteins, respectively in ECV account for defibrinogenation and the strong *in vitro* pro-coagulant activity. Further, glutaminyl cyclase, aspartic protease, aminopeptidase, phospholipase B, vascular endothelial growth factor, and nerve growth factor were reported for the first time in ECV. The proteome composition of ECV was well correlated with its biochemical and pharmacological properties and clinical manifestations observed in *Echis* envenomed patients. Neutralization of enzymes and pharmacological properties of ECV, and immuno-cross-reactivity studies unequivocally point to the poor recognition of <20 kDa ECV proteins, such as PLA_2_, subunits of snaclec, and disintegrin by commercial polyvalent antivenom.

## Introduction

**Figure 1:**
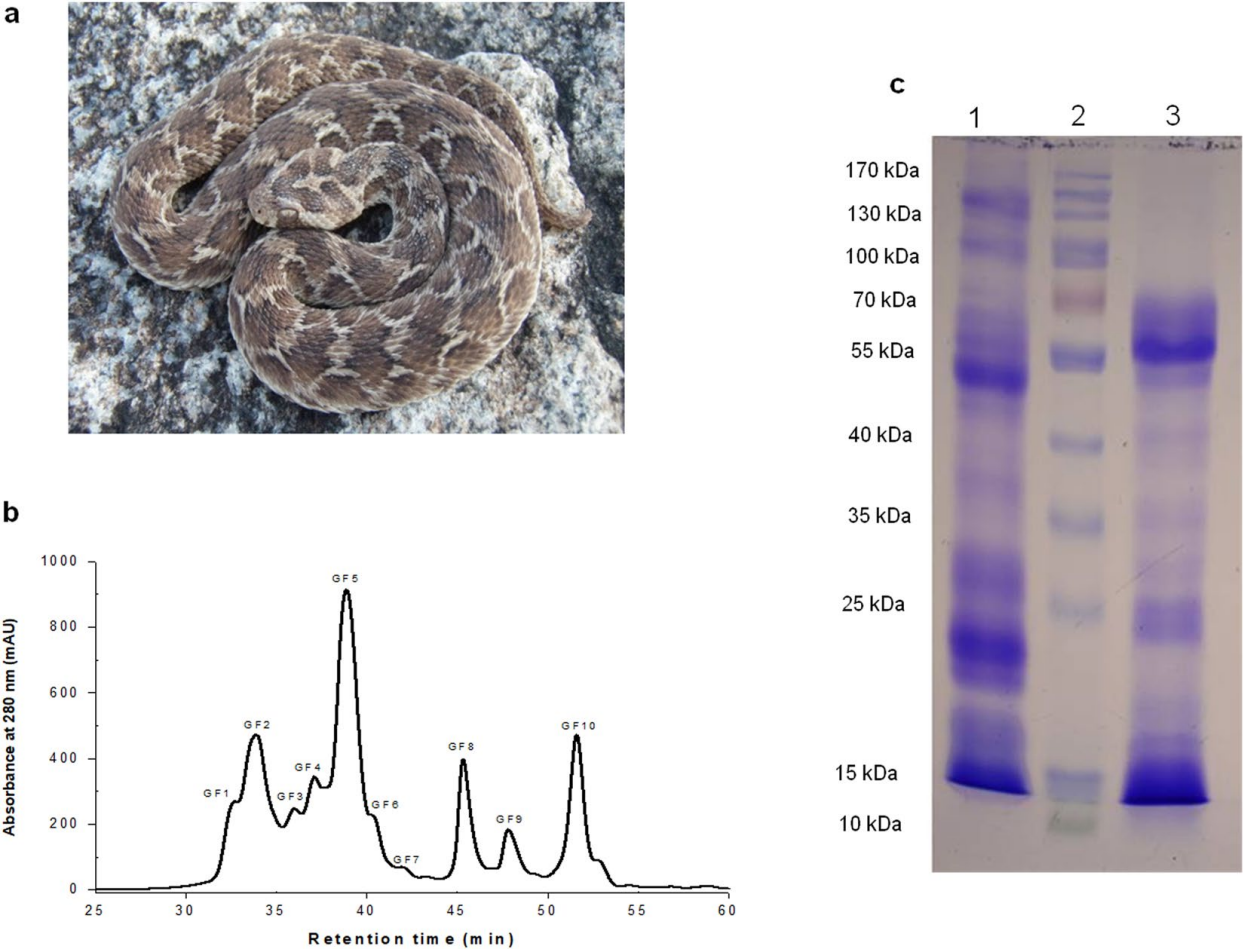
(**a**) Photograph of an Indian saw scaled viper (*Echis carinatus*). Photograph courtesy of Romulus Whitaker, Nov, 2010. (**b**) Fractionation of ECV (2.0 mg dry weight) on a Shodex KW- 803 gel filtration column coupled to Dionex Ultimate 3000 UHPLC system (Thermo Fisher Scientific, USA). The flow rate was maintained at 10 ml/h at room temperature (~23 °C) and fractions of 0.25 ml were collected. (**c**) SDS-PAGE analysis of crude ECV. Lane 1, 2 and 3 represents crude ECV (non-reduced), protein molecular markers and crude ECV (reduced) respectively.

According to the World Health Organization, snakebite is a major health challenge in tropical and sub-tropical countries including India, and therefore, it is considered as a neglected tropical disease^1^. The “big four” venomous snakes – *Daboia russelli*, *Naja naja*, *Echis carinatus*, and *Bungarus caeruleus* account for the most snakebite mortality and morbidity in the Indian subcontinent^2^. Notably, envenomation by the saw scaled viper (*E*. *carinatus;* EC) (Fig. 1a) also requires immediate medical attention; and therefore, it is considered as a category I medically important snake in India. Because the clinical symptoms and pathophysiological manifestations following envenomation may vary depending on the geographical origin of the snake, unveiling the complex venom proteome of a snake from a particular locale is extremely important for correlating venom composition with pharmacological properties and the pathophysiology of envenomation. *Echis carinatus* is divided into two sub-species namely *E*. *c*. *sochureki* and *E*. *c*. *carinatus* ^3^ of which the latter is confined to peninsular India and is responsible for many fatalities in parts of western and southern India^4^. Although Casewell and co-workers reported venom proteome composition of *E*. *c*. *sochureki* (from United Arab Emirates) by combination of transcriptomics and proteomics approaches^5^; however, to the best of our knowledge, no work has yet explored the venom proteome profile of *E*. *c*. *carinatus* from peninsular India or compared the data with pharmacological properties as well as clinical manifestations in *Echis* envenomed patients.

Although traditional methods (chromatographic separation of venom proteins followed by biochemical assays) have long been used to investigate snake venom composition^6–8^, they have been inadequate to unravel the detail composition of complex venom proteomes. In contrast, the modern proteomic approach has eliminated most shortcomings of classical chromatographic techniques when followed by biochemical assays to identify the venom proteins^9–13^. Although tandem mass spectrometry-based protein identification is limited because of the absence of sufficient sequences in the protein database repository, it can contribute to the quantitative identification of venom proteomes^14^.

The administration of polyvalent antivenom (raised against the big four venomous snakes in India) is the only choice of therapy against snake envenomation^15^. For an antivenom therapy to be completely efficacious, all toxins of the venom would need to be recognized by the antivenom with neutralization of the toxicity and venom-induced lethality^10–12,16^. Because clinical manifestations of snake envenoming depend primarily on venom composition, the efficacy of commercial equine polyvalent antivenom (PAV) also depends on the geographical source of venom used for antivenom production. Therefore, the commercial antivenom may lack its potency to neutralize venom induced lethality in geographical locales situated distant from the source of immunizing venoms^17^. Consequently, the venom-antivenom cross-reactivity is important for the information it provides to improve the efficacy of commercial antivenom and the subsequent efficient treatment of snakebites^11,12^.

In this study, the previously unexplored venom proteome composition of *E*. c. *carinatus* venom (ECV) from southern India was unraveled by proteomic analysis and the venom toxin profile was correlated with biochemical and pharmacological properties of venom and the clinical manifestations that follow ECV envenomation in southern India. An attempt has also been made to determine the venom-antivenom cross-reactivity and potency of commercial PAVs in neutralizing the enzymatic activities and some pharmacological properties of ECV and its fractions.

## Results and Discussion

### Proteomic analysis reveals the occurrence enzymatic and non-enzymatic proteins in ECV

De-complexation of ECV by U-HPLC gel filtration chromatography on Shodex KW-803 resolved it into 10 peaks (Fig. 1b). SDS-PAGE analyses of crude venom under reduced and non-reduced conditions confirmed the dynamic range of proteins present in the venom under study (Fig. 1c). The analysis of the protein bands from crude ECV suggested the predominance of proteins in the mass ranges of 55–90 kDa and 10–20 kDa. Viperidae snake venom proteins in the mass range of 55–90 kDa are usually represented by snake venom metalloprotease (SVMP), L-amino acid oxidase (LAAO), and nucleotidase (NT) or adenosine monophosphatase (AMPase)^18–20^, whereas the venom proteins in the mass range of 10–20 kDa include phospholipase A_2_ (PLA_2_), disintegrins, and snaclec monomers^21–23^. The MALDI-TOF-MS analysis of gel filtration fractions of ECV at different mass ranges suggested the presence of wide range of distinct ions (307) in the *m*/*z* range of 5.0–150.2 kDa (Supplementary Table S1). However, several of these ECV proteins may not be the real gene products but at least some of these may have produced by autocatalysis of venom proteins. Noteworthy, MALDI-TOF-MS data representing a “fingerprint” of ECV from southern India would be useful for comparing venom proteins from congeneric snakes from different locales. ECV proteins in the mass range of 41–100 kDa represent 47.5% of venom proteins followed by very high molecule weight (>100 kDa) proteins (23.5%) whereas the low molecular mass proteins (5–20 kDa) are the least abundant ECV proteins (Supplementary Table S1).

Tandem mass spectrometry coupled to protein database search has evolved as a gold standard for snake venom protein identification^11–13^. Although, several isotope labeling methods such as SILAC, ICAT, and iTRAQ, are applied for mass spectrometry-based protein quantification^24^, albeit some limitations of these techniques may restrict their use for quantification of venom proteins^24,25^. On the contrary, the label free protein quantification techniques such as spectral count (MS2) and area-based (MS1) methods in addition to circumvent the above disadvantages are also comparable to other approaches of protein quantification including isotope labeling and mass spectral peak intensities^26^. Nevertheless, label free protein quantification methods are not devoid of technical difficulties like protein size, availability of trypsin cleavage sites along the protein, and lack of a comprehensive snake venom protein databases^24,27^. Moreover, the relative abundance of a protein in snake venom invariable depends on the number of peptides identified which in turn is influenced by the degree of sequence similarity between homologous sequences present in the database^27^. Therefore, in this study to avoid unambiguous protein identification as well as to improve the protein quantification, the semi-tryptic sequences were also considered, the spectral count as well as the area under peptides were normalized by the number of theoretical peptides^13,24,27^, and at least one overlapping distinct peptide was considered for ECV protein identification^13^. Further, to enhance the relevance of the venom proteomic analysis we have compared the ECV proteome with the published reports on venom proteome composition of congeneric snakes from other locales^5^.

**Figure 2:**
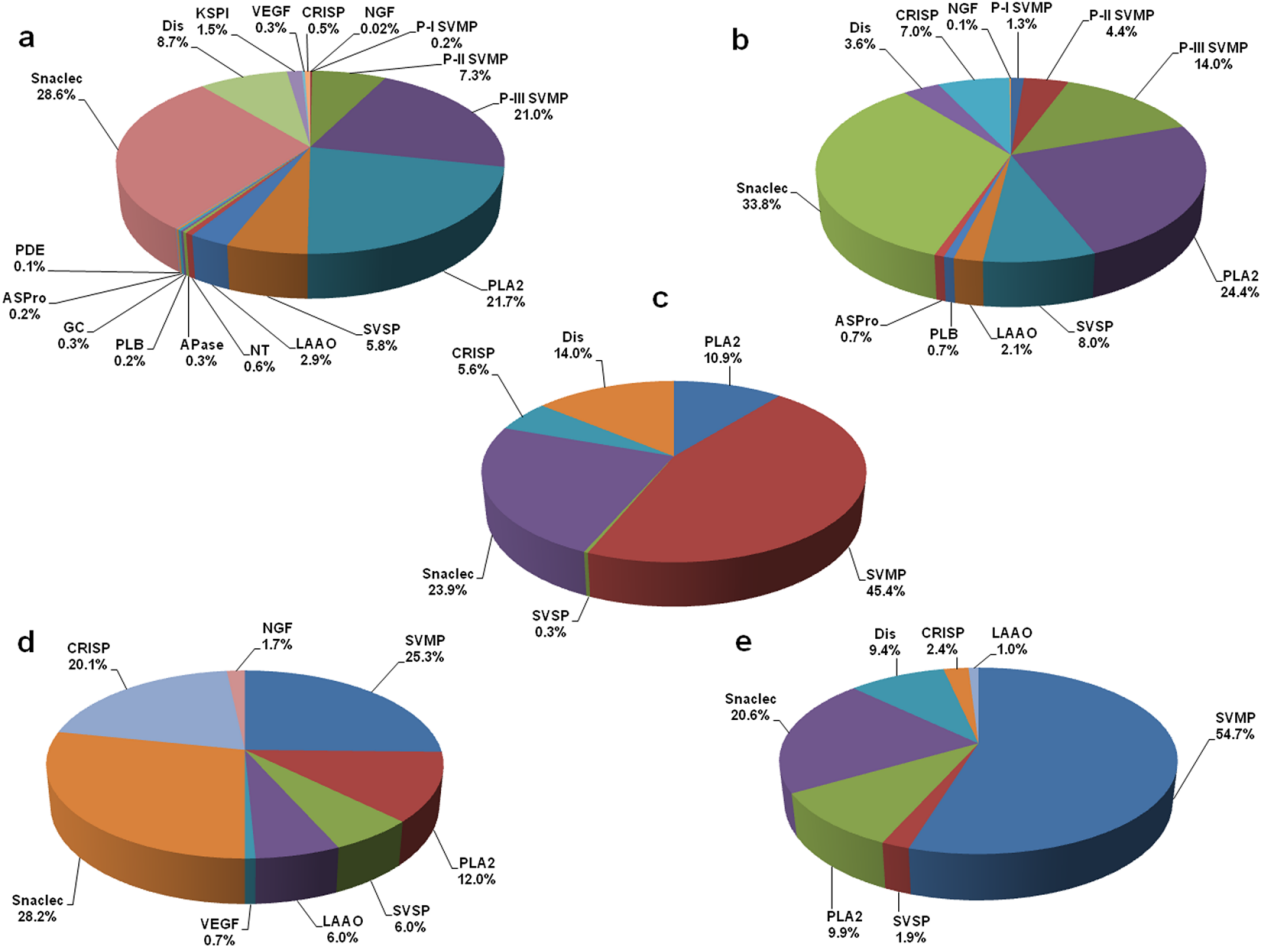
Protein family composition of ECV proteome. The pie chart represents the relative occurrence of different enzymatic and non-enzymatic protein families of ECV when the data was searched against NCBI protein entries with taxonomy set to (**a**) Viperidae (taxid 8689), (**b**) *Echis* (taxid 8699), (**c**) *E*. *carinatus* (taxid 40353), and (**d**) Transcriptome Shotgun Assembly (TSA) sequences of *E*. *coloratus* (BioProject: PRJEB2884). (**e**) Composition of *E*. *c*. *sochurecki* (UAE) venom as predicted from venom gland transcriptomic analysis^5^.

In the present study, approximately 10% of the collected MS/MS spectra were matched to peptide sequences with high confidence. The collected MS/MS spectra usually show 10–30% high confidence matching to peptide sequences and several factors, for example existence of MS/MS spectra with non-canonical fragment ions that are excluded by database search engines, absence of the peptide sequences in the target database, and unanticipated post-translational modifications can be attributed to this effect^28^. The ESI-LC-MS/MS analyses of tryptic peptides generated from the gel filtration fractions of ECV showed sequence similarities with more than 1500 proteins deposited in Viperidae venom protein databases. Our stringent protein identification process; however, eliminated redundancies and led to a final recognition of 90 distinct enzymatic and non-enzymatic proteins belonging to 15 snake venom protein families of ECV (Table 1, Fig. 2a, Supplementary Table S2). Conversely, analysis of LC-MS/MS data against the genus (*Echis*) database entries in the NCBI returned with 665 protein entries; however, only 47 distinct proteins belonging to 10 snake venom families were identified in ECV proteome (Fig. 2b, Supplementary Table S3a,b). Further, only 22 distinct proteins distributed across 6 venom protein families were identified in the target *Echis carinatus* database (Fig. 2c, Supplementary Table S4a,b). Noteworthy, searching the LC-MS/MS data against the Transcriptome Shotgun Assembly (TSA) sequences of congeneric *Echis coloratus* venom resulted in identification of only 14 proteins belonging to 8 venom protein families (Fig. 2d, Supplementary Table S5a,b). Because mass-spectrometry-based proteomic analysis is database-dependent^29^; therefore, it is quite reasonable to understand that the difference in number of proteins identified in ECV proteome by searching the different protein databases was dependent on the database entry of genus or species-specific snake venom proteins. Consequently, several protein families such as vascular endothelial growth factor (VEGF), Kunitz-type protease inhibitor (KSPI), aminopeptidase (APase), NT, and glutaminyl cyclase (GC) of ECV identified by searching the Viperidae databases were not identified in *Echis* as well as *E*. *carinatus* venom protein databases due to limitations in genus and species-specific database entries.

**Table 1:** Summary of different proteins identified in Indian Saw Scaled Viper (*E*. *c*. *carinatus*) venom by tandem mass spectrometry analysis of gel filtration peaks against Viperidae family of proteins.

S. No.	Accession No.	Description	Source organism	−10lgP	Coverage (%)	Distinct Peptides	Avg. Mass (Da)	GF peak(s)
**Enzymatic proteins**								
**P- I Metalloprotease** (**1 protein**)								
1	gi|727360735	Snake venom metalloproteinase K, partial	*Echis coloratus*	102.69	8	1	48851	1,3–7
**P- II Metalloprotease** (**5 proteins**)								
1	gi|297594086	Metalloproteinase	*Echis carinatus sochureki*	114.32	34	2	12578	3–8,10
2	gi|297593820	Metalloproteinase, partial	*Echis carinatus sochureki*	113.74	15	3	28319	1,3–7
3	gi|297594078	Metalloproteinase	*Echis carinatus sochureki*	100.95	9	2	53640	3,7–10
4	gi|297594068	Metalloproteinase	*Echis coloratus*	98.47	31	2	13935	3,4–8
5	gi|297594070	Metalloproteinase, partial	*Echis carinatus sochureki*	77.02	8	1	27679	5,7–10
**P- III Metalloprotease** (**18 proteins**)								
1	gi|297593950	Metalloproteinase	*Echis coloratus*	165.72	13	3	68942	1–3,7
2	gi|300079900	Factor X activator heavy chain	*Daboia russellii russellii*	153.11	11	2	69521	1–7,9,10
3	gi|297593790	Metalloproteinase, partial	*Echis carinatus sochureki*	149.47	17	3	63999	1–8,9
4	gi|297593830	Metalloproteinase, partial	*Echis carinatus sochureki*	147.78	22	3	35963	1,3–8
5	gi|297593798	Metalloproteinase, partial	*Echis carinatus sochureki*	145.07	25	5	39004	3,7.8,10
6	gi|297593862	Metalloproteinase, partial	*Echis coloratus*	130.71	14	1	57183	1,3,7
7	gi|162329887	Chain A, Crystal Structure Of Russell’s Viper Venom Metalloproteinase	*Daboia russellii russellii*	124.48	13	1	47646	1–3,4–8
8	gi|83523634	Group III snake venom metalloproteinase	*Echis ocellatus*	119.25	15	2	69598	1–3,7–8
9	gi|297593842	Metalloproteinase, partial	*Echis carinatus sochureki*	116.46	26	2	27821	1,3,7–8
10	gi|297593794	Metalloproteinase	*Echis carinatus sochureki*	115.08	7	1	68205	1,3–7
11	gi|297593836	Metalloproteinase, partial	*Echis carinatus sochureki*	107.73	9	3	41105	1,7–8
12	gi|297593852	Metalloproteinase, partial	*Echis coloratus*	103.55	10	1	56407	3,7,8,10
13	gi|320579375	Group III snake venom metalloproteinase, partial	*Echis ocellatus*	103.03	9	1	63254	3,8
14	gi|297593822	Metalloproteinase, partial	*Echis carinatus sochureki*	99.09	11	2	35935	1,2
15	gi|297593854	Metalloproteinase	*Echis coloratus*	91.01	5	1	69286	1,3,7,8,10
16	gi|297593858	Metalloproteinase	*Echis coloratus*	72.99	5	1	68727	3
17	gi|52000738	Snake venom metalloproteinase HT-1	*Crotalus ruber ruber*	62.04	7	1	23601	3,7
18	gi|297593984	Metalloproteinase, partial	*Echis pyramidum leakeyi*	53.13	5	1	49052	3,7
**Phospholipase A**_**2**_ (**12 proteins**)								
1	gi|298351762	Basic phospholipase A_2_ 3	*Daboia russellii russellii*	198.21	69	2	13687	7,8,10
2	gi|81174981	Basic phospholipase A_2_ VRV-PL-V	*Daboia russellii russellii*	183.48	46	1	13587	8,10
3	gi|71912229	Phospholipase A_2_	*Daboia russellii limitis*	183.45	75	4	15353	7,8,9,10
4	gi|82096307	Acidic phospholipase A_2_ EC-I	*Echis carinatus*	178.05	59	3	15523	6,7
5	gi|149243451	Chain A, Crystal Structure Of A New Isoform Of Phospholipase A_2_ From Russells Viper	*Daboia russellii russellii*	121.82	60	2	13452	3,10
6	gi|87130858	Phospholipase A_2_-III	*Daboia russellii russellii*	83.27	27	2	13441	7
7	gi|163311140	Ecarpholin S	*Echis carinatus*	69.3	25	2	13819	10
8	gi|59727030	D1E6b Phospholipase A_2_	*Cerrophidion godmani*	68.37	10	1	15970	7
9	gi|1041577114	Phospholipase A_2_ 1 h	*Agkistrodon contortrix contortrix*	63.46	10	1	15961	7,10
10	gi|1041577423	Phospholipase A_2_ 1 h	*Agkistrodon piscivorus*	60.88	6	1	15748	7
11	gi|71912223	Basic phospholipase A_2_	*Daboia russelii*	43.99	13	1	15461	10
12	gi|157834128	Chain A, Anticoagulant Class Ii Phospholipase A_2_	*Vipera russelli russelli*	33.79	25	1	13626	10
**Serine protease** (**11 proteins**)								
1	gi|134129	Factor V activator RVV-V alpha	*Daboia siamensis*	115.68	34	6	26182	3–7,9,10
2	gi|311223824	Serine beta-fibrinogenase-like protein precursor	*Daboia siamensis*	91.84	11	2	28035	2–7
3	gi|297593766	Serine protease, partial	*Echis coloratus*	76.47	8	2	28863	2–3,6–8
4	gi|293491172	Serine protease, partial	*Echis ocellatus*	73.07	14	1	28236	2,3,6,7
5	gi|306756038	Serine protease VLSP-3 precursor	*Macrovipera lebetina*	66.96	14	1	28352	3
6	gi|297593764	Serine protease, partial	*Echis carinatus sochureki*	63.79	10	2	26218	7,8,10
7	gi|13959655	Venom serine proteinase-like protein 2	*Macrovipera lebetina*	57.74	6	1	28894	3,7
8	gi|297593786	Serine protease, partial	*Echis coloratus*	56.82	5	1	28184	3,7,8
9	gi|306756034	Serine protease VLSP-1 precursor	*Macrovipera lebetina*	54.18	6	1	28702	3
10	gi|298351881	Snake venom serine protease rhinocerase	*Bitis rhinoceros*	53.95	28	2	9978	3,7
11	gi|297593736	Serine protease, partial	*Echis coloratus*	51.71	8	2	26001	3
**L-amino acid oxidase** (**3 proteins**)								
1	gi|194400545	Secreted L-amino acid oxidase precursor	*Daboia russelii*	212.00	29	10	56888	1–10
2	gi|727360693	L-amino acid oxidase B variant 2	*Echis coloratus*	115.20	15	1	35024	1
3	gi|1002590400	PREDICTED: L-amino-acid oxidase isoform × 1	*Protobothrops mucrosquamatus*	104.80	8	1	57159	1,3,7,10
**Phosphodiesterase** (**1 protein**)								
1	gi|586829527	Phosphodiesterase	*Macrovipera lebetina*	124.14	8	1	96181	1,3
**Nucleotidase** (**1 protein**)								
1	gi|211926756	Ecto-5’-nucleotidase	*Gloydius brevicaudus*	102.06	9	1	64434	1,3,6
**Glutaminyl cyclase** (**1 protein**)								
1	gi|380846517	Glutaminyl-peptide cyclotransferases	*Daboia russelii*	84.26	6	2	42116	6,9
**Aspartic proteases** (**1 protein**)								
1	gi|109287596	Renin-like aspartic protease	*Echis ocellatus*	66.41	5	2	43872	2,3
**Aminopeptidase** (**1 protein**)								
1	gi|743538216	Endoplasmic reticulum aminopeptidase 1-like protein	*Crotalus horridus*	86.52	5	4	107397	1–2,4,6
**Phospholipase B** (**1 protein**)								
1	gi|727360709	Phospholipase B	*Echis coloratus*	80.51	6	3	64541	1,2,3
**Non enzymatic proteins**								
**Snaclec** (**20 proteins**)								
1	gi|55670410	Chain B, Structure Of Ems16-Alpha2-I Domain Complex	*Echis multisquamatus*	123.97	45	6	15099	1,2–9
2	gi|300490474	P68 alpha subunit	*Daboia russellii limitis*	122.92	23	1	18108	1
3	gi|727360769	C-type lectin J	*Echis coloratus*	116.83	23	2	18279	1–8,10
4	gi|758377456	C-type lectin-like protein 3A	*Macrovipera lebetina*	115.87	37	2	18079	1–3,7–8
5	gi|38493055	Chain A, Crystal Structure Of Ems16	*Echis multisquamatus*	114.95	49	4	15874	1,2,3,7,8
6	gi|300490472	P68 beta subunit	*Daboia siamensis*	110.56	32	1	16910	1–3,5–8
7	gi|802148	Echicetin beta subunit	*Echis carinatus*	104.7	31	1	14869	3–7,9
8	gi|32452854	Echicetin A-chain	*Echis carinatus*	103.17	31	5	15363	1,2–9
9	gi|40889261	Chain B, Echicetin	*Echis carinatus*	101.88	29	1	14922	3,4,6–9
10	gi|758377450	C-type lectin-like protein 3B	*Macrovipera lebetina*	94.95	26	1	17043	1,5–8
11	gi|998226344	C-type lectin 2	*Bitis arietans*	93.67	32	1	17891	1,3,4,6,7
12	gi|300490462	Dabocetin alpha subunit	*Daboia russellii russellii*	86.53	27	2	17493	3,4,7
13	gi|218526484	Snaclec A13	*Macrovipera lebetina*	77.52	15	2	15308	3,5–8
14	gi|82175557	Snaclec salmorin subunit A	*Gloydius brevicaudus*	53.46	6	1	17293	1,3,7,8
15	gi|300490478	P31 alpha subunit	*Daboia russellii limitis*	52.92	14	1	18179	3,4
16	gi|578004418	Lebecin B, partial	*Macrovipera lebetina*	52.69	18	1	17553	4–6,8
17	gi|538259843	C-type lectin factor IX/X binding protein B subunit, partial	*Protobothrops flavoviridis*	43.47	8	1	12795	3,4–7
18	gi|205275155	C-type lectin	*Echis ocellatus*	36.75	9	1	16882	3
19	gi|2829697	Snaclec echicetin subunit alpha	*Echis carinatus sochureki*	36.05	14	1	15803	3,7
20	gi|82174836	Snaclec coagulation factor IX/factor X-binding protein subunit B	*Echis carinatus*	34.43	7	1	14372	1,7,9
**Disintegrin** (**6 proteins**)								
1	gi|82194569	Disintegrin	*Echis carinatus*	151.29	89	1	7127	3–8,10
2	gi|544584743	Disintegrin EC6 subunit alpha	*Echis carinatus sochureki*	114.32	34	2	12578	3–8,10
3	gi|182705265	Disintegrin schistatin-like subunit B	*Echis carinatus*	104.43	52	2	6961	1,3,6–8,10
4	gi|82203514	Disintegrin gabonin-1	*Bitis gabonica*	94.79	18	2	13792	4–5,7
5	gi|182705262	Disintegrin VLO4	*Macrovipera lebetina obtusa*	91.67	46	1	7108	7
6	gi|110346540	RTS-containing short disintegrin, partial	*Echis ocellatus*	46.91	23	1	4588	10
**Vascular endothelial growth factor** (**1 protein**)								
1	gi|48429241	Snake venom vascular endothelial growth factor toxin ICPP	*Macrovipera lebetina*	57.74	10	1	12574	3,5–6
**Cystine rich secretory proteins** (**3 proteins**)								
1	gi|190195321	Cysteine-rich seceretory protein Dr-CRPK	*Daboia russelii*	125.59	22	4	26688	7
2	gi|803374854	Cysteine-rich venom protein	*Echis coloratus*	116.84	19	1	24699	7
3	gi|1041577503	Cysteine-rich secretory protein 1	*Agkistrodon piscivorus*	84.05	12	1	26681	7
**Kunitz type serine protease inhibitor** (**3 proteins**)								
1	gi|159883522	Trypsin inhibitor-3 precursor	*Daboia siamensis*	118.27	51	2	9443	10
2	gi|239977245	Kunitz-type serine protease inhibitor B1	*Daboia siamensis*	69.75	31	1	9318	10
3	gi|123913154	Kunitz-type serine protease inhibitor 4	*Daboia russellii russellii*	67.17	14	1	9145	7–8,10
**Nerve growth factor** (**1 protein**)								
1	gi|400499	Venom nerve growth factor	*Daboia russellii russellii*	61.53	21	2	13283	3

The proteomic composition of *E*. *c*. *carinatus* venom, when searched against species-specific protein databases (Fig. 2c) corroborates well with the published transcriptomic data of *E*. *c*. *sochureki* venom gland (Fig. 2e)^5^; nevertheless, the minor variation in the relative abundances of venom proteins in these two congeneric snakes reflects the sub-species-specific difference and/or geographical variation in snake venom composition. Notably, the LAAO identified in *E*. *c*. *sochureki* venom by transcriptomic approach^5^ was not identified by species-specific database analysis of ECV, although this enzyme was identified in the venom under study by biochemical analysis as well as by genus-specific database search (Fig. 2b, Supplementary Table S3a,b). This result suggests the absolute necessity of species-specific transcriptomic database in proteomic study^29,30^. Further, identification of few homologous protein isoforms in ECV by searching the TSA sequences of *E*. *coloratus* may presumably be explained on the basis of venom variability between the two species of *Echis*.

Due to lack of species-specific (*E*. *c*. *carinatus*) transcriptomic database we have applied both MS1 and MS2-based quantitative approaches for determining the relative protein abundance in ECV and compared their results with each-other. The relative abundance of each protein class calculated by both MS1 (area based) and MS2 (spectral count)-based methods did not show significant deviation (Supplementary Fig. S1a–h); therefore, the relative abundance of ECV proteins is presented as an average data determined from MS1 and MS2-based methods (Fig. 2a–d). The alignments of MS/MS-derived peptide sequences with the homologous proteins from Viperidae, *Echis*, *E*. *carinatus* and translated *E*. *coloratus* TSA sequence databases are shown in Supplementary Fig. S2a–d. Some of the MS/MS spectra corresponding to mass (Da) 1250.5966 (2^+^), 118.5608 (3^+^), 733.3646 (2^+^), 798.3912 (2^+^), 929.4719 (2^+^), 1741.8192 (2^+^), 2942.252 (2^+^), 907.4837 (2^+^) and 1103.5645 (2^+^) are shown in Supplementary Fig. S3.

It is noteworthy to mention that among the LC-MS/MS-identified protein families, GC, aspartic protease (ASPro), APase, phospholipase B (PLB), VEGF, and nerve growth factor (NGF) were shown to be present in ECV for the first time. By proteomic analysis, the enzymatic proteins identified in ECV include SVMP, PLA_2_, snake venom serine proteases (SVSP), LAAO, NT, APase, PLB, GC, ASPro, and phosphodiesterase (PDE) (Fig. 2c).

Proteases of snake venoms are broadly classified as SVMP and SVSP^31^. The proteomic analysis suggested the presence of enzymes from both groups of proteases in ECV; nevertheless, the species-specific database search demonstrated predominance of SVMP group in ECV (Fig. 2c) which corroborates well with the venom proteins composition of *E*. *c*. *sochureki* determined by transcriptomic analysis (Fig. 2e). Remarkably, the relative abundance of SVMP in ECV (Fig. 2c) was found to be lower as compared to SVMP content determined by proteomic analysis in the venoms of its congeneric species such as *E*. *coloratus*, *E*. *ocellatus*, *E*. *pyramidum leakeyi* and *E*. *c*. *sochureki* ^5^. Interestingly, the relative abundance of SVMP in ECV varies significantly depending on the target databases (Fig. 2a–d) owing to different number of entries of venom proteins in these databases. The biochemical analyses suggested that GF 1, followed by GF 2, exhibited the highest metalloprotease activity (Table 2), which was supported by LC-MS/MS analysis of the gel filtration peaks (Table 1). Further, SVMPs are grouped to PI, PII and PIII classes based on their size and domain structure^32^. Analysis of LC-MS/MS data against Viperidae venoms showed that ECV was predominated by PIII-SVMPs (17.9% of the ECV proteome) (Table 1; Fig. 2c). Because SVMP is the most abundant component in *Echis* venom, therefore the significant variation in SVMP content among *Echis* spp. may lead to differences in severity of clinical manifestations and pharmacological activity post *Echis spp*. bite.

**Table 2:** *In vitro* pharmacological properties and associated enzymatic activities of crude ECV and its GF fractions. Values are mean ± SD of triplicate determinations. Average PT and APTT of control PPP was determined at 18 ± 0.0 s and 35.0 ± 0.9 s, respectively. Platelet count of control (untreated) sample was 2.5 ± 0.1 × 10^6^ cells/ml. Significance of difference with respect to control (^a^p < 0.05). The ( + ) sign indicates presence whereas (−) sign indicates absence of fibrinogen clotting activity.

Venom fraction	Protein content (% yield)	Pharmacological Properties						Enzyme activity			
		Plasma clotting activity (Ca^2+^-clotting time) U/mg (x10^4^)	Coagulant (C) or Anticoagulant (AC)	PT (s)	APTT (s)	Percent decrease in platelet count (%)	Fibrinogen clotting activity	PLA_2_ (U/mg × 10^3^)	SVMP (U/mg)	TAME (U/mg)	BAEE (U/mg × 10^3^)
ECV	100.0 ± 0.4	4.3 ± 0.0004	C	15.5 ± 0.7^a^	15.5 ± 2.12^a^	62.1 ± 3.1^a^	+	7.2 ± 0.6	0.6 ± 0.01	5.0 ± 0.2	0.8 ± 0.04
GF-1	6.9 ± 0.4	32.3 ± 0.0006	C	17.0 ± 0.0^a^	17.0 ± 1.41^a^	56.7 ± 2.5^a^	−	0.4 ± 0.02	2.2 ± 0.06	−	1.6 ± 0.006
GF-2	9.0 ± 0.2	26.7 ± 0.0007	C	17.5 ± 0.4	24.5 ± 2.12^a^	66.3 ± 2.9^a^	+ +	0.3 ± 0.01	1.4 ± 0.04	103.3 ± 5.2	4.1 ± 0.3
GF-3	4.7 ± 0.3	26.3 ± 0.0010	C	16.0 ± 0.0^a^	21.5 ± 0.7^a^	31.8 ± 1.1^a^	+	0.5 ± 0.03	0.9 ± 0.04	140.0 ± 7.0	3.5 ± 0.1
GF-4	6.9 ± 0.3	20.3 ± 0.0010	C	16.5 ± 0.1^a^	24.5 ± 0.7^a^	35.7 ± 1.6^a^	+	0.1 ± 0.05	0.7 ± 0.01	61.7 ± 3.1	2.6 ± 0.09
GF-5	15.2 ± 0.5	18.3 ± 0.0005	C	18.5 ± 0.7	30.5 ± 0.7	25.7 ± 0.8^a^	+	0.3 ± 0.2	0.3 ± 0.02	80 ± 3.6	1.9 ± 0.07
GF-6	5.1 ± 0.3	14.3 ± 0.0008	C	15.5 ± 0.7^a^	26.0 ± 1.4^a^	26.4 ± 0.7^a^	−	9.9 ± 0.4	0.5 ± 0.09	−	1.2 ± 0.04
GF-7	11.3 ± 0.8	15.3 ± 0.0008	AC	18.0 ± 0.1	32.0 ± 0.2	34.5 ± 1.2^a^	−	12.1 ± 0.7	0.4 ± 0.02	−	0.8 ± 0.01
GF-8	15.1 ± 0.3	134.3 ± 0.0014	AC	18.0 ± 0.2	30.5 ± 0.7	51.1 ± 1.9^a^	−	18.1 ± 0.9	0.5 ± 0.03	−	0.5 ± 0.03
GF-9	7.0 ± 0.2	128.0 ± 0.0014	AC	18.0 ± 0.2	38.5 ± 0.7	12.7 ± 0.3	−	12.1 ± 0.7	0.2 ± 0.02	−	0.2 ± 0.01
GF-10	4.4 ± 0.1	11.7 ± 0.0007	AC	18.0 ± 0.1	42.5 ± 2.12	50.3 ± 2.1^a^	−	5.9 ± 0.2	0.5 ± 0.02	−	0.04 ± 0.01

Significant esterolytic activity exhibited by proteins was eluted in GF 2 to GF 5 (Table 2), which may be correlated with the presence of quantitatively higher amounts of pro-coagulant serine proteases, in these fractions (Table 1). Depending on target database search the relative abundance of SVSPs was found to vary in ECV proteome (Fig. 2a–d, Table 1). The relative abundance of SVSP (determined by searching the species-specific protein databases) in ECV was found to be comparable with the relative abundance of this class of protease determined by proteomic analysis in the venom of other species of *Echis* ^5^.

The molecular mass of PLA_2_ from snake venoms is reported to be in the range of 10–15 kDa^33^. In accordance with its molecular weight, it was mostly eluted in the GF 7–9 fractions (Table 1). In the case of Russell’s viper venom (RVV), PLA_2_s have been shown to interact with high molecular weight components of venom leading to their elution in the initial gel filtration fractions of RVV^11,12^. Interestingly, the present study also found the elution of trace quantities of PLA_2_ in GF 3 (Table 1). The relative abundance of PLA_2_ determined by analyzing data against Viperidae and *Echis* venoms (2a-b) was found to be two-fold then its relative abundance determined by species-specific database search (2c) which may presumably due to less entry of this important class of venom protein in latter database. The relative abundance of PLA_2_ in ECV (10.9%) determined by species-specific database search (Fig. 2c) was comparable to the relative abundance of the same enzyme in venoms of *E*. *coloratus*, *E*. *ocellatus*, and *E*. *c*. *sochureki* albeit PLA_2_ content of *E*. *pyramidum leakeyi* venom was found to be higher^5^ which may have an evolutionary significance^34^.

ATPase, ADPase, AMPase, and PDE are poorly characterized, high molecular mass (>50 kDa) proteins of snake venom^20,35^. Our biochemical assays showed that ECV contains ATPase (6.3 ± 0.006 × 10^4^ U/mg), ADPase (3.6 ± 0.300 × 10^4^ U/mg), AMPase/nucleotidase (8.2 ± 0.4 × 10^3^ U/mg), and PDE (18.6 ± 0.08 U/mg) enzyme activities. Nevertheless, ATPase and ADPase from ECV could not be identified by proteomic analysis (in the present study) owing to a limited protein database deposition^11,12^ or by transcriptomic analysis of venom glands of *Echis spp* ^5,30^. Occurrence of PDE in ECV could not be identified when the LC-MS/MS data were searched against the genus and species-specific databases (Fig. 2b,c) or by the transcriptomic analysis of *E*. *c*. *sochureki* venom gland for the reason stated above; however, searching the MS/MS data against Viperidae venom proteins resulted in identification of PDE as a minor component of the ECV (Table 1, Fig. 2a)^11,12,36^.

LAAOs are high molecular weight (60–150 kDa) snake venom enzymes^19,37^. ECV demonstrated an LAAO specific activity of 0.3 ± 0.01 U/mg. Depending upon the venom protein / transcriptomic database search the relative abundance of LAAO isoenzymes in ECV proteome demonstrated significant variation (2.1–6%) (Fig. 2a,b,d, Table 1, Supplementary Tables 2–5). However, due to limited database entry LAAO could not be identified in ECV proteome by species-specific database search (Fig. 2c, Supplementary Table S4a,b). The LAAO was also reported to be a minor component in venom of other species of *Echis* ^5,30^.

Hyaluronidase activity, at the tested dose of 3 µg/ml, was not found by the biochemical assay or by the LC-MS/MS analysis of ECV or by proteomic and transcriptomic analyses of venoms of *Echis spp* ^5^. Nevertheless, Urs *et al*.^38^ and Girish *et al*.^39^ demonstrated the presence of hyaluronidase in ECV, albeit they used a much higher concentration of venom (200 µg/ml) in their enzyme assay.

Proteomic analysis identified non-enzymatic proteins in ECV, including snaclec, disintegrin, Kunitz-type protease inhibitor (KSPI), VEGF, cysteine-rich secretory protein (CRISP), and NGF (Fig. 2a–d).

C-type lectin (snaclec), the Ca^2+^-dependent non-enzymatic proteins^9,40^ of snake venom, are found to be the most abundant non-enzymatic group of proteins in the ECV proteome; however, depending on non-redundant protein / TSA sequence database search their relative occurrence in ECV was demonstrated to vary between 24–34% (Fig. 2a–d, Table 1, Supplementary Tables 2–5). A comparison shows that the snaclec content of ECV proteome (Fig. 2c) was 3–4 folds higher as compared to the snaclec content in other species of *Echis* ^5^. Because snaclec are one of the major components of *Echis* venom responsible for adverse pharmacological effects in bite victims (see below) therefore, this species-specific variation in snaclec content in different species of *Echis* may have a profound clinical significance.

The disintegrins are cysteine rich non-enzymatic proteins characterized with a conserved arginine-glycine-aspartic acid (RGD) motif^41^. They were the second most abundant non-enzymatic proteins in ECV (Fig. 2c) and among the identified disintegrins, 5 isoforms (gi|82194569, gi|544584743, gi|182705265, gi|82203514, and gi|182705262) belong to dimeric disintegrin sub-family. The disintegrin content of ECV (14%), determined by species-specific protein database search (Fig. 2c), was found to be slightly higher than the disintegrin content determined by proteomic as well as transcriptomic analysis of *E*. *c*. *sochureki* venom^5^.

Apart from the above mentioned proteins, single isoforms of actin and myosin were also identified by proteomic analysis against Viperidae family of venom proteins (data not shown). Nevertheless, they are not venom components and may be contamination in the ECV from the venom extraction. Considering their likely origin and low abundance (0.3%), they were not considered in calculating the relative abundance of proteins in ECV.

### The proteome composition of ECV is well correlated with its *in vitro* pharmacological properties (coagulopathy) and clinical manifestations after envenomation

Venoms of the Viperidae family of snakes are reported to influence the hemostatic system of the victim, a leading cause of death among viper bite patients^7,11,42^. Nevertheless, blood clotting effects are more pronounced in cases of *Echis* bite, compared to Russell’s viper bite^43^. Local symptoms of envenomation including persistent local swelling, edema, bleeding/haemorrhage, and pain at the bite site and systemic envenoming symptoms such as hemotoxicity and coagulopathy have been observed as severe clinical symptoms in *E*. *carinatus* bite patients in southern India and other parts of the Indian subcontinent^43–45^ (personal communication from Dr. A. Zachariah, Christian Medical College, Vellore, and R. Whitaker, herpetologist, Madras Snake Park, Chennai).

The yield of venom per milking from an adult saw-scaled viper (0.8–1.0 feet in length) is approximately 12 mg^43^. Therefore, the concentration of ECV in the blood of an adult human after a full bite would be expected to be in the range of 2.5–3.0 µg/ml. The assays of blood coagulation activity suggest that crude ECV (3.0 µg/ml) and GF 1–6 fractions were strongly pro-coagulant, whereas the proteins from GF 8 and 9 demonstrated anticoagulant activity (Table 2). The pro-coagulant activity of ECV is also more pronounced than the activity displayed by crude RVV^12,43^. SVMPs and SVSPs have been shown to influence the hemostatic system by activation of blood coagulation factor Xa, factor V, prothrombin, and by virtue of thrombin-like activity^11,46^. The abundance of SVMPs and SVSPs in the ECV proteome (Fig. 2c), compared to the RVV proteome^12^, makes this venom more pro-coagulant. The SVMPs and SVSPs are usually mid- and high-molecular mass proteins (>33 kDa) of Viperidae venoms^18,47,48^, and they were separated in the GF 1–6 fractions (Table 2), which is supported by previous reports^11,12^. Subsequently, the low molecular mass proteins (<30–10 kDa) that are eluted in GF 7–10 gel filtration fractions, exhibited anticoagulant activity (Table 2). These fractions are predominated by PLA_2_s, KSPIs, and disintegrins (Table 1), and their anticoagulant properties have been well characterized^23,49,50^.

**Figure 3:**
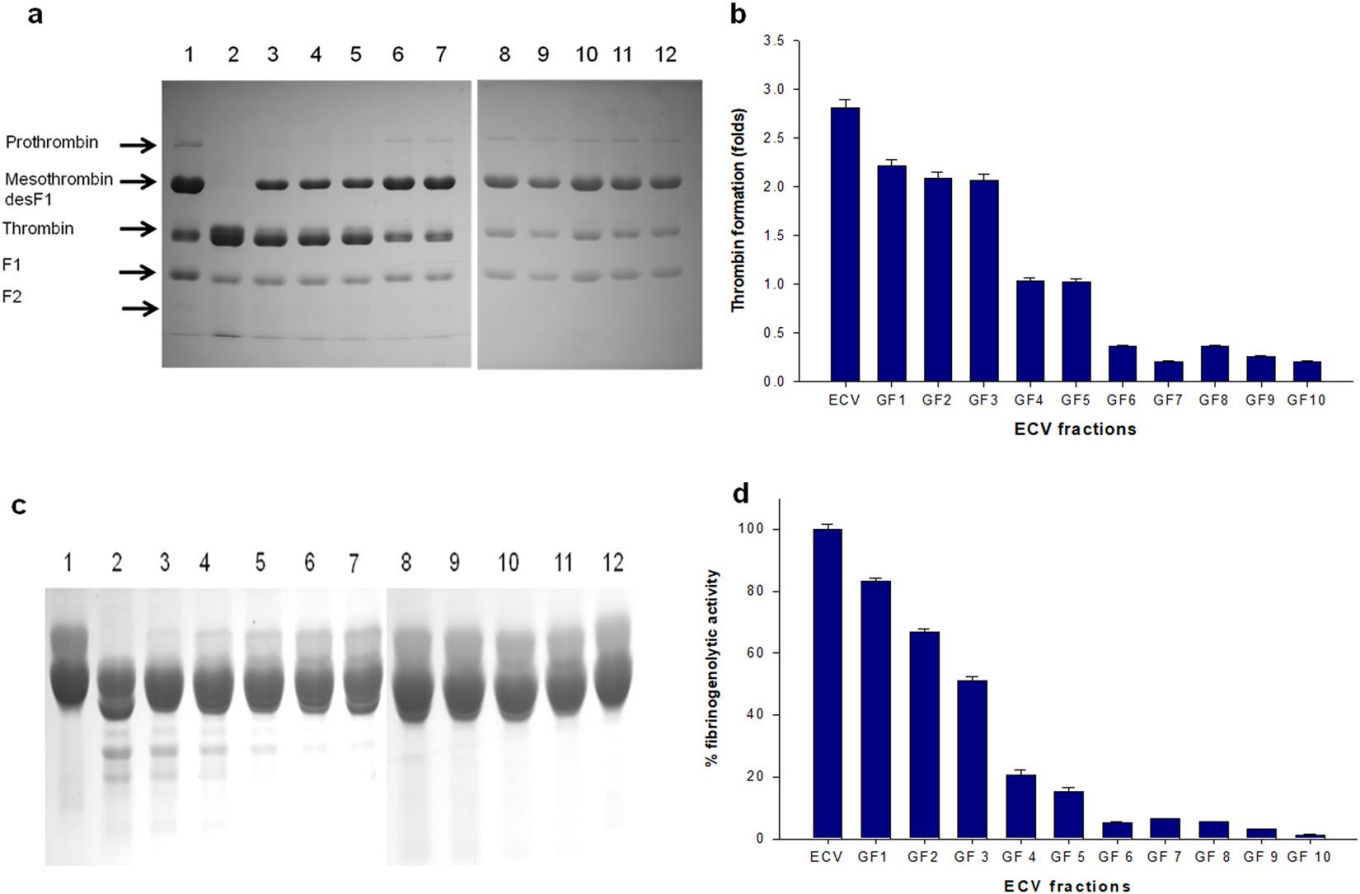
Prothrombin activation (thrombin formation) and fibrinogenolytic activity by ECV and GF fractions. (**a**) 12.5% SDS-PAGE analysis of prothrombin activation by ECV (3 µg/ml) and gel filtration fractions (0.5 µg/ml) under non-reducing conditions. Lane 1, prothrombin incubated with 1X PBS for 3 h at 37 °C (control); lanes 2–12, products of prothrombin hydrolysis by crude ECV (3 µg/ml) and GF 1–10 (0.5 µg/ml), respectively. Two 12.5% SDS-PAGE gels run under identical conditions were cropped and fused together. The full length unedited gels images are shown in Supplementary Figure S4. (**b**) The trace quantity of thrombin formed by auto degradation of control prothrombin was considered as 1 and other values (fold increase in thrombin formation determined by densitometry scanning of gel using Image Quant TL software 8.1) were compared to that. Values are mean ± SD of triplicate determinations. (**c**) 12.5% SDS-PAGE analysis of fibrinogen degradation by ECV and its gel filtration fractions. Lane 1 contains fibrinogen incubated with 1X PBS for 3 h at 37 °C (control), lanes 2–12 contain fibrinogen incubated with ECV (3 µg/ml) and GF 1–10 (0.5 µg/ml), respectively. Two 12.5% SDS-PAGE gels run under identical conditions were cropped and fused together. The full length unedited gels images are shown in Supplementary Figure S5. (**d**) Percentage of fibrinogenolytic activity was calculated by measuring the degradation of the Aα band of fibrinogen. The disappearance of Aα band of fibrinogen by crude ECV was considered as 100% activity and the band intensities of the treated samples were compared to that. Values are mean ± SD of triplicate determinations.

SVMPs and SVSPs primarily interfere with the blood coagulation cascade of victim/prey^42,46^ that ultimately leads to consumption coagulopathy and the prolongation of whole blood clotting time of *E*. *carinatus* bite patients in southern India^45^ (personal communication from Dr. A. Zachariah, CMC, Vellore). Interestingly, SVMPs were detected in all gel filtration fractions by proteomic analysis (Table 1) and by biochemical assay (Table 2). Carinactivase^18^ and Ecarin^50^ possessing prothrombin activation property have been well characterized as high molecular weight (>50 kDa) SVMPs from ECV. Although our stringent protein identification process did not identify the above proteases in ECV, the remarkable prothrombin activation exhibited by ECV and its gel filtration fractions, GF 1–3 (Fig. 3a,b), may have been due to the presence of several pro-coagulant SVMPs and RVV-X activator-like proteins in ECV (Tables 1 and 2).

Fibrinogenolytic activity was prominent in ECV, and especially, the GF 1–5 fractions due to elution of proteases (SVMPs and SVSPs) in these fractions (Fig. 3c,d). The occurrence of these enzymes was also evident from biochemical and proteomic analyses (Tables 1 and 2). Furthermore, the above fractions are rich in esterase activities, predominantly BAEE-esterase activity (Table 2), which is often exhibited by SVSPs that possess fibrinogenolytic activity^47,48^. The crude venom (or GF 1–5) proteins degraded the Aα chain of fibrinogen leaving β and γ chains intact (Fig. 3c), which suggests that ECV is predominated by α-fibrinogenase. Further, it has been well documented that prothrombin-activating SVMPs and procoagulant SVSPs together play a predominant role in inducing coagulation and in *in vivo* defibrinogenating activities^42,47,48^. Interestingly, tryptic peptides VIGGDECNIN and TSTYIAPLSLPSSPPR of Factor V activator pro-coagulant serine protease isoenzymes^47^ and a thrombin-like serine protease (Russelobin)^48^, detected in this ECV proteome, have also been shown to cause *in vivo* defibrinogenation in mice^47,48^. Further, crude ECV and its gel filtration fractions (GF 2–5) exhibited remarkable fibrinogen clotting activity leading to the formation of tenuous fibrin clots (Table 2). This pharmacological property can be correlated to the presence of Russelobin-like serine proteases (gi|311223824)^48^ in these fractions (Table 1). Taken together, defibrinogenation (consumption of fibrinogen) and prothrombin as well as factor V (FV) activation, induced by the abundant SVMPs and SVSPs in ECV, may be responsible for the prolongation of blood coagulation in bite victims, which is a major clinical symptom of *E*. *c*. *carinatus* envenomation^46^.

SVMPs are often associated with additional cysteine-rich, and disintegrin domains that are cleaved off by proteolysis or autolysis during venom secretion^32^. Further, in some cases, C-type lectin domains (snaclec) are found to be linked with PIII-SVMPs by disulfide linkage^32^. Among them, the CRISP and snaclec domains are reported to trigger inflammation by recruiting inflammatory cells at the bite site^51^. Further, Ecarpholin (accession no. gi|163311140), a basic PLA_2_ isolated and characterized from ECV was predicted to be myotoxic in nature^21^. It was also detected by LC-MS/MS analysis in the current study (Table 1). Since this basic myotoxic PLA_2_ can induce edema in the footpad of experimental Swiss albino mice^52^, the persistent local swelling and edema observed in *Echis* bite patients^43,44^ can be correlated with the presence of significant amounts of CRISPs, snaclecs and PLA_2_s in the ECV proteome (Fig. 2a–c).

Further, other symptoms of envenomation, such as hemoptysis, haematemesis, and haematuria, observed in a few patients bitten by *E*. carinatus^43,44^ (personal communication from Dr. A. Zachariah, CMC, Vellore) may be correlated to the existence of PLA_2_s, SVMPs, and SVSPs in ECV (Table 1, Fig. 2c). Hemorrhage and local tissue damage is yet another important clinical symptom observed in *Echis* bite patients^43,44,53^. Among the SVMP classes, PII and PIII SVMPs are associated with non-metalloproteinase domains (disintegrin and cysteine-rich) that are reported to target extracellular matrix (ECM) and cause hemorrhage^54^. Therefore, this clinical feature can be correlated with the abundance of both the sub-classes of SVMPs in southern India ECV proteome. Hypotension and shock, the two other prominent clinical symptoms of *Echis* envenomation^43^ are probably attributed to venom ATPase and VEGF (Fig. 2c)^55^ as well as coagulopathy and bleeding.

A decrease in the circulatory platelet count (thrombocytopenia) after *Echis* bite is a very common clinical finding;^43,44^ and ECV and its gel filtration fractions induced loss of platelet integrity *in vitro*, albeit this effect was more pronounced by GF 1 and 2 proteins (Table 2) containing abundant SVMPs and snaclecs (Tables 1 and 2). Because the above two proteins collectively account for snake venom-induced thrombocytopenia^12,56,57^, it could be concluded that a high proportion of the SVMPs and snaclecs in ECV (Fig. 2c) are liable for the observed thrombocytopenia induced by *E*. *carinatus* venom in human (Table 2). Although ECV did not exhibit significant aggregation or deaggregation of washed platelets (data not shown), it rapidly (<60 s) induced agglutination of platelet rich plasma (PRP). A heterodimeric snaclec Echicetin, purified from venom of *E*. *carinatus* from unknown regions of India and identified in southern India ECV (accession nos. gi|802148, gi|32452854, gi|40889261, gi|2829697) (Table 1) binds specifically to platelet glycoprotein (GP)Ib and causes platelet agglutination^22^.

### Assessment of immuno-cross-reactivity of ECV with commercial polyvalent antivenom indicated poor recognition of low molecular weight venom proteins

**Figure 4:**
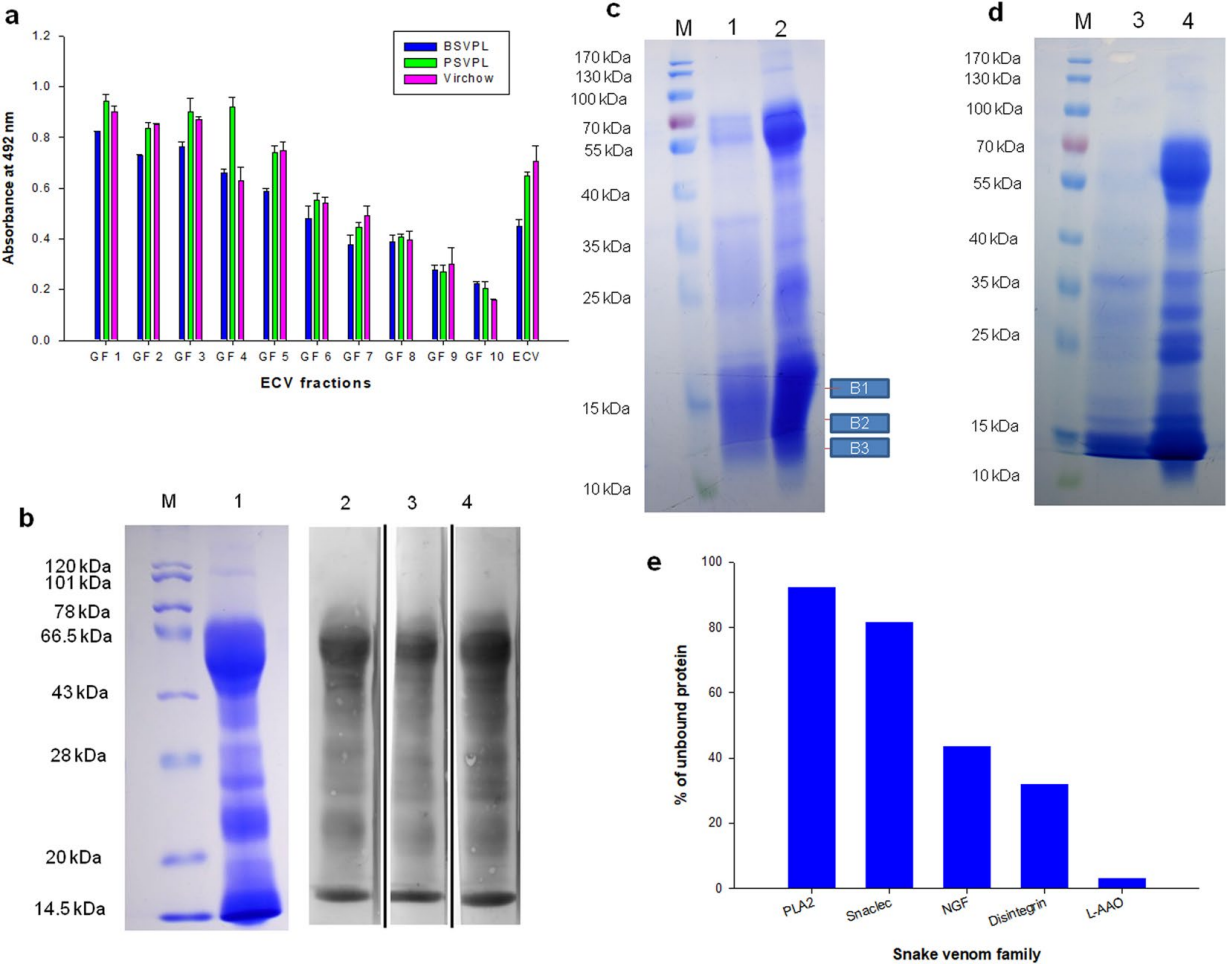
(**a**) Immunological cross-reactivity of ECV and GF fractions with commercial PAVs by ELISA. Values are mean ± SD of triplicate determinations. (**b**) Immunoblot analysis of ECV against commercial PAVs. Lanes M and 1 contain protein molecular markers and ECV (100 µg, reduced), respectively. Lanes 2, 3, and 4 represent blots immuno-detected by PAVs – BSVPL, PSVPL and Virchow, respectively. (**c**) 12.5% SDS-PAGE analysis of affinity column unbound fraction. Lanes 1 and 2 represent unbound proteins and crude ECV (500 µg, reduced), respectively. Lane M contains protein molecular markers. (**d**) 12.5% SDS-PAGE analysis of immunoaffinity column bound ECV proteins. Lanes 3 and 4 represent immunoaffinity column bound ECV proteins and crude ECV, respectively. Lane M contains protein molecular markers. (**e**) Percentage of unbound protein less recognized by PAV (PSVPL) coupled with immunoaffinity column.

To date, antivenom remains the only choice for the treatment of snakebite. Nevertheless, the safety and efficacy of antivenoms are major concerns for efficient antivenom treatment. Therefore, the immuno-recognition of different commercial polyvalent antivenoms towards ECV and its gel filtration fractions was studied by ELISA, Western blot, and immuno-chromatographic (second generation antivenomics) analyses. Results from the ELISA experiment indicated that immuno-recognition of high (>45 kDa) and mid molecular weight (20–45 kDa) proteins was significantly higher (p < 0.05) than the recognition of low molecular mass proteins (<20 kDa) of ECV for all tested PAVs (Fig. 4a). This finding essentially reflects the poor immunogenicity of low molecular weight ECV proteins, or alternatively, the geographical source of ECV used for raising antivenom was different than the locale of the ECV used in this study. The corroboration of results from ELISA and Western blotting analysis also showed that ECV proteins ranging in mass from 10–20 kDa were less recognized by the tested antivenoms (Fig. 4b). The immuno-chromatographic approach also showed that mostly the low molecular weight (<20 kDa) venom proteins were not efficiently captured by PAV (Fig. 4c,d). Subsequently, by LC-MS/MS analysis (antivenomics study) these low molecular mass ECV proteins (Fig. 4c) were identified as PLA_2_s, α- and β-subunits of snaclecs, disintegrins, and NGFs (Table 3), and the percent of these proteins did not bind to PAV-immuno-affinity column is shown in Fig. 4e. Because PLA_2_s as well as snaclecs play a significant role in ECV-induced toxicity and adverse pharmacological effects in bite victim;^44,58,59^ therefore, least neutralization and/or recognition of these components of ECV by commercial PAV is a serious concern for effective antivenom therapy.

**Table 3:** List of proteins identified by in-gel trypsin digestion and subsequent ESI-LC-MS/MS analysis of the ECV proteins poorly recognized by commercial PAVs. To enhance the ECV protein coverage, the data was searched against Viperidae family of proteins.

Sl no.	Accession	Description	Protein family	Source organism	Score	Coverage	Unique peptides	MW [kDa]	SDS-PAGE band
1	gi|149243451	Basic phospholipase A_2_	PLA_2_	*Daboia russelii*	1136.5	79.3	7	13.6	3
2	gi|82096307	Acidic phospholipase A_2_	PLA_2_	*Echis carinatus*	1050.4	61.7	10	15.5	1–3
3	gi|38493055	Snaclec EMS16 subunit alpha	Snaclec	*Echis multisquamatus*	428.8	49.6	5	18.2	1
4	gi|298351762	Basic phospholipase A_2_	PLA_2_	*Daboia russelii*	307.3	52.8	5	13.7	1–3
5	gi|802148	Snaclec echicetin subunit beta	Snaclec	*Echis carinatus*	305.9	24.3	2	14.9	3
6	gi|32452854	Snaclec echicetin subunit alpha (fragment)	Snaclec	*Echis carinatus*	292.8	20.0	2	15.7	1–3
7	gi|300490462	Snaclec dabocetin subunit alpha	Snaclec	*Daboia russellii russellii*	253.9	46.1	3	17.5	3
8	gi|400499	Venom nerve growth factor	NGF	*Daboia russelii*	236.4	21.3	2	13.3	1
9	gi|55670410	Snaclec EMS16 subunit beta	Snaclec	*Echis multisquamatus*	184.4	15.5	3	17.7	1
10	gi|82194569	Disintegrin (Fragment)	Disintegrin	*Echis carinatus*	91.7	43.7	2	7.1	1,3
11	gi|194400545	L-amino-acid oxidase (fragments)	LAAO	*Daboia russelii*	88.3	6.6	2	46.3	1–2
12	gi|163311140	Basic phospholipase A_2_ homolog ecarpholin	PLA_2_	*Echis carinatus*	67.6	25.4	2	13.8	3

The major challenge in managing *Echis* bite patients is the efficient treatment of local swelling, which does not subside even after several vials of antivenom treatment, thus leaving the victims partially crippled (personal communication from R. Whitaker, Chennai). PLA_2_s are responsible for edema induction or local swelling^44^, whereas snaclecs can induce thrombocytopenia^12,56^. Therefore, the poor immunogenicity of these ECV components may be one of the reasons that explain the persistent local swelling in bite victims even after several vials of antivenom therapy. Henceforth, strategies must be designed either by inclusion of antibodies specifically raised against these low molecular weight toxic venom components or improving their antigenicity so as to mitigate the deleterious effect of the proteins for better hospital management of *Echis* bite patients. Further, presence of hemorrhagic SVMPs in ECV contributes to the formation of stable neutrophil extracellular trap (NET) at the bite site which forms a barrier for the free flow of blood and prevents antivenom from reaching the damaged site. Therefore, although high molecular weight SVMPs are well recognized and neutralized, antivenom treatment fails in efficient reversal of ECV induce local toxicity and swelling^60^.

### Neutralization of enzymatic activity and pharmacological properties of ECV by commercial polyvalent antivenom highlights the requirement of a well-**d**esigned immunological protocol for antivenom production

**Figure 5:**
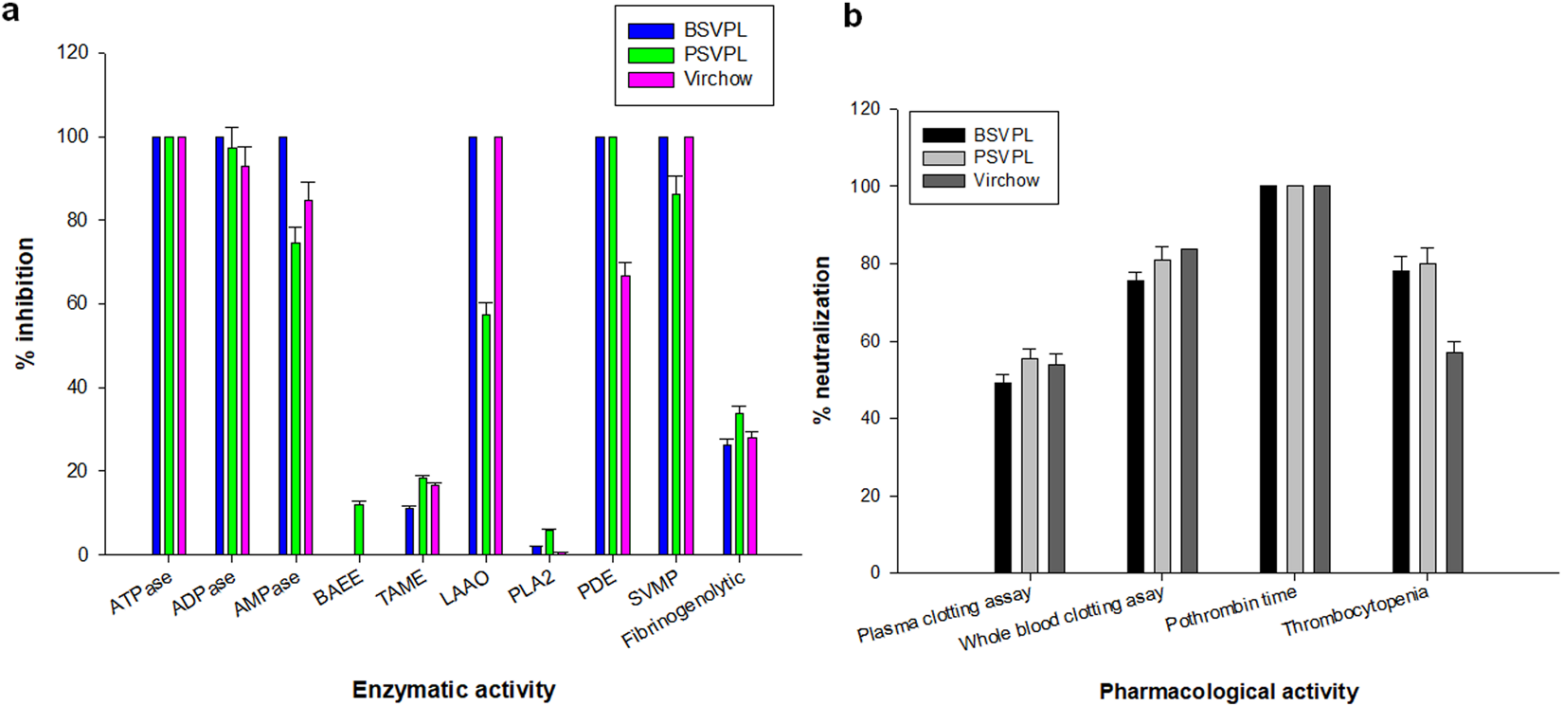
Neutralization of (**a**) enzyme activities and (**b**) pharmacological properties of ECV by PAVs at 1:10 (venom: antivenom) ratio. Values are mean ± SD of triplicate determinations.

Our previous studies have demonstrated a good correlation between enzyme function and the pathophysiology of snake envenomation^11–13^. The efficient treatment of ECV bite will depend on effective neutralization of enzymatic activities and pharmacological properties of this venom. Commercial PAVs showed variable inhibitions of the enzymatic and pharmacological properties of ECV, which again, may be correlated to the source of ECV used for antivenom production. The enzymatic activities exhibited by high molecular mass proteins (>50 kDa), such as LAAO, PDE, SVMP, ATPase, ADPase, AMPase were efficiently neutralized by the antivenom (Fig. 5a); however, the enzymatic activities of mid and low molecular weight proteins such as PLA_2,_ SVSP, Nα-p-Tosyl-L-arginine methyl ester hydrochloride (TAME), and Nα-Benzoyl-L-arginine ethyl ester hydrochloride (BAEE) exhibited mostly by SVSPs were least neutralized by antivenom (Fig. 5a). Interestingly, these proteins (except PLA_2_) were well recognized by PAV (Fig. 4b,c,d), suggesting that the catalytic sites of the enzymes may be poor immunogens. Further, the negligible neutralization of PLA_2_ activity suggests that the low molecular mass (~14–15 kDa) pharmacologically active proteins of ECV also serve as poor antigens (Fig. 5a).

Interference with the hemostatic system is the major pharmacological effect of ECV (Table 2); therefore, the potency of commercial antivenoms to neutralize this property of crude ECV was also investigated. All of the PAVs at 1:10 (venom:antivenom) efficiently neutralized the pro-coagulant activity and loss of platelet integrity induced by ECV (Fig. 5b). This may be correlated to the efficient neutralization of pro-coagulant SVMPs (Fig. 5b), indicating a good correlation between metalloprotease activity of Viperidae venom and the above pharmacological properties^12^.

## Materials and Methods

*E*. *c*. *carinatus* (saw-scaled viper) venom (ECV) from Irula Snake Catchers Industrial Cooperative Society, Chennai, South India, was a gift from Premium Serum and Vaccines Pvt. Ltd, India. Lyophilized powders of polyvalent snake antivenom raised in horses against venom cocktail of Big four Indian snakes (*Naja naja*, *Daboia russelli*, *Bungarus caeruleus* and *Echis carinatus*), vials were purchased from Bharat serum and Vaccines Ltd. (BSVPL) (Batch no. A05315029, expiry date: January, 2019), Premium Serum and Vaccines Pvt. Ltd. (PSVPL) (Batch no. 012015, expiry date: December, 2018) and Virchow Biotech Pvt. Ltd. (Virchow) (Batch no. 012005, expiry date: May, 2018). Prothrombin time (PT) and activated partial thromboplastin time (APTT) were determined using Liquiplastin and Liquicelin-E kits purchased from Tulip Diagonstics, Mumbai, India. All other chemicals used were analytical grade and procured from Sigma Aldrich, USA.

### Fractionation of ECV through gel filtration chromatography and SDS-PAGE analysis of crude ECV

2 mg of ECV (dry weight) was dissolved in 20 mM Tris-Cl, pH-7.4 buffer containing 150 mM NaCl (buffer A) and centrifuged at 10,000 rpm for 10 min. The supernatant was filtered through 0.2 µ membrane syringe filter and protein content was estimated by the Lowry method^61^. Then 0.1 ml filtrate containing 1.5 mg of protein was fractionated on a Shodex KW-803 gel filtration column (8 × 300 mm, 5 µm) equipped with Dionex Ultimate 3000 UHPLC system (Thermo Fisher Scientific, USA). The column was pre-equilibrated with the above buffer and the flow rate was adjusted to 15 ml/h at room temperature (~23 °C). The elution of protein was monitored at 280 nm and fractions of 0.25 ml were collected. The peaks were pooled for the enzymatic activity assay, pharmacological characterization, and LC-MS/MS analysis. Protein content of crude ECV and fractions was determined^61^ and from a standard curve of BSA, the concentration of unknown protein was determined at 660 nm.

The crude ECV was analyzed on 12.5% SDS-PAGE in both reduced and non-reduced conditions. Protein bands were visualized by PhastGel Blue R stain (GE Healthcare, Sweden). The molecular masses of the ECV proteins were also determined by MALDI-TOF-MS analysis (4800 MALDI TOF/TOF™ Analyzer, Applied Biosystems) as described earlier^12,48^. The masses were determined in the m/z ranges of 5–20, 21–40, 41–100, and >100 kDa.

### Proteomic identification of ECV proteome by LC-MS/MS analysis of GF fractions

To identify the venom proteome, 80 µg of each gel filtration fraction was subjected to ESI-LC-MS/MS analysis^11–13^. The venom proteins were digested with sequencing grade trypsin (13 ng/μL in 10 mM ammonium bicarbonate containing 10% acetonitrile) at an enzyme substrate ratio of 1:30 at 37 °C for 18 h. The tryptic peptides were desalted, concentrated using ZipTip C_18_ (Merck, USA) and reconstituted in 0.1% formic acid. They were separated on a Zorbax 300SB-C_18_ analytical column (75 μm × 150 mm, 3.5 μm, Agilent) at a flow rate of 300 nL/min applying the following mobile phase gradient: from 11% B for 5 min, 11 to 25% B in 20 min, 25 to 53% B in 16 min, 53 to 100% B in 5 min, 100% B for 4 min, and then 11% B for 4 min. Solvent A and B were 0.1% formic acid and 80% acetonitrile containing 0.1% formic acid, respectively. The eluted peptides were then analysed on an LTQ Orbitrap Discovery hybrid mass spectrometer (ThermoFisher Scientific, Bremen, Germany) interfaced to an Agilent 1200 HPLC via a Nanomate Triversa (Advion BioSciences, Ithaca, NY). The ionization voltage was set at 1.7 kV.

The raw data was acquired and processed by Xcalibur software (ThermoFisher Scientific, Bremen, Germany) in a data-dependent acquisition (DDA) mode with 1 MS survey scan followed by 5 MS/MS scans. The full-scan MS spectra were acquired in the FT mode in the scan range of m/z 300−2000 (lock mass was set to 445.12 corresponding to polysiloxane) with a resolution of 30, 000 (full width at half-maximum). MS/MS fragmentation was collision-induced dissociation (CID) in linear mode with the following triggering conditions: minimum signal intensity, 10,000; charge state, +2, +3; maximum injection time for MS/MS, 500 ms; and isolation width, 2 amu.

The LC-MS/MS data were searched independently against the entries in non-redundant NCBI database with taxonomy set to (a) Viperidae (taxid: 8689 with 56,902 protein entries), (b) *Echis* (taxid: 8699 with 788 protein entries) and (c) *Echis carinatus* (taxid: 40353 with 162 protein entries). The data were analyzed by PEAKS 8.5 software (Bioinformatics Solutions Inc., Ontario, Canada). Carbamidomethylation of cysteine, oxidation of methionine, deamidation of asparagine and glutamine and pyro-glu of N-terminal glutamine residues were set as variable modifications. Precursor and fragment mass tolerances were set to 10 ppm and 0.8 Da, respectively, up to two missed cleavages were allowed and non-specific cleavage at one end (semi-tryptic) was considered. The false discovery rate (FDR) was kept very stringent (0.1%). To exclude the contaminating proteins from identification, the contaminant database with 115 protein entries (ftp://ftp.thegpm.org/fasta/cRAP/crap.fasta) was included in the database search process. All the redundant peptides were removed from the data set and thereafter each protein entry was manually verified. The following criteria were set for the purposes of protein identification: (a) only matching proteins and peptides showing a −10 log P value ≥ 30 and 20, respectively, and (b) occurrence of at least one overlapping distinct peptide.

The relative abundance of the venom proteins was determined by MS1 (area under peptide) as well as MS2 (spectral count) based label-free methods. For both the methods, the sum of areas or the spectral count was normalized by number of theoretical peptides and the normalized values were calculated using equation 1. The number of theoretical peptides for each identified protein was determined using MassSorter v3.1 software.

1 $$\begin{array}{c}Mean\,area\,or\,spectral\,count\,for\,protein\,X\\ =\,\frac{\Sigma \,area\,or\,spectral\,count\,against\,MS1\,peptides\,of\,X}{number\,of\,theoretical\,peptides\,of\,X}\end{array}$$

Thereafter, the relative abundance of a protein in a particular GF fraction was calculated using equation 2:

2 $$\begin{array}{c}{Relative}\,{abundance}\,{of}\,{X}\,{in}\,{GF}\,{fraction}\,{Y}\\ =\,\frac{mean\,area\,or\,spectral\,count\,of\,X\,in\,Y}{total\,mean\,areas\,or\,spectral\,count\,of\,all\,proteins\,in\,Y}\times protein\,yield\,( \% )\,of\,Y\end{array}$$

The average of the relative protein abundance of ECV determined by two different methods (MS1 areas-based and MS2 spectral count) was considered to represent the relative abundance of ECV venom proteome.

In addition, the MS/MS data were searched against Transcriptome Shotgun Assembly (TSA) sequences of *Echis coloratus* venom, the only translated *Echis* protein sequence database in NCBI (BioProject: PRJEB2884, 87 translated protein entries) and analyzed using PEAKS 8.5 software. The search parameters were the same as stated under LC-MS/MS data analysis. After removing the redundant peptides the translated protein entries were manually verified. The matching proteins and peptides showing a −10 log P value ≥ 30 and 20, respectively and occurrence of at least one overlapping distinct peptide were the pre-requisite for protein identification^12,13^. The relative abundances of ECV proteins were determined by MS2 mean spectral count and MS1 area-based methods (equations 1 and 2). The data was presented as an average of the relative protein abundance of ECV determined by two different methods.

### Biochemical characterization

Esterolytic activity of crude ECV and its GF fractions were checked by the spectrophotometric method using Nα-Benzoyl-L-arginine ethyl ester hydrochloride (BAEE) and Nα-p-Tosyl-L-arginine methyl ester hydrochloride (TAME) as substrate^48^. One unit of TAME and BAEE-esterase activity is defined as an increase in absorbance of 0.01 at 254 nm and 244 nm, respectively, during the first 5 min of the reaction at 37 °C. PDE activity was assayed by the spectrophotometric method using bis-*p*-nitrophenyl phosphate as a substrate^62^. One unit of PDE activity is defined as micromoles of p-nitrophenol released per min. The protease activity of crude ECV and its GF fractions was determined by incubating 3.0 µg/ml of crude ECV or 0.5 µg/ml of GF fraction or 1X PBS (control) with fibrinogen (2.5 mg/ml) for 3 hours at 37 °C. The fibrinogen degradation products were separated by 12.5% SDS-PAGE (reduced) and percent fibrinogenolytic activity was calculated by measuring the degradation of the Aα chain of fibrinogen. The band intensity of Aα chain of fibrinogen molecule after crude ECV treatment was considered as 100% fibrinogenolytic activity and other values were compared to that. The protein band intensities were measured by Image Quant TL 8.1 software (GE Healthcare, Sweden).

PLA_2_ activity of crude ECV (3 µg/ml) and GF fractions (0.5 µg/ml) was assayed by the turbidometric method^63^. One unit of PLA_2_ activity is defined as a decrease in 0.01 absorbance at 740 nm after 10 min of incubation. L-kynurenine was used as a substrate for screening of L-amino acid oxidase (LAAO) activity of crude ECV (3 µg/ml) and GF peaks (0.5 µg/ml). The unit of LAAO activity was defined as nmol kynurenic acid produced/min under the assay conditions and specific LAAO activity was expressed as unit/mg protein^11,12^.

ATPase and ADPase activity of crude ECV (3 µg/ml) and GF fractions (0.5 µg/ml) was assayed by the method of Williams and Esnouf^64^ with slight modifications as described by Mukherjee *et al*.^11^ The 5′-nucleotidase (AMPase) activity was determined according to the protocol of Sinsheimer and Koerner^65^ with slight modifications described in our previous publication^11^. One unit of ATPase/ADPase/AMPase activity was defined as μM of Pi released per min at 37 °C.

Hyaluronidase activity of crude ECV (3 µg/ml) was assayed as described previously^12^. One unit of enzyme activity was defined as a decrease in turbidity by 1% as compared to the control and activity was expressed as U/mg protein^66^.

SVMP activity was assayed by using azocasein as a substrate. Crude ECV (3 µg/ml) or GF peaks (0.5 µg/ml) were added to reaction mixture and incubated at 37 °C for 10 min. The activity was checked by the spectrophotometric method and specific activity was expressed as ΔA_450nm_/min/mg protein^11^.

### Pharmacological characterization

Goat blood obtained from slaughter house was collected in 3.8% tri sodium citrate and the blood was centrifuged at 4300 rpm for 10 min at 4 °C^11,63^. The pellet was discarded and the yellowish supernatant was termed as platelet poor plasma (PPP) and it was used within 4 hours after its collection. The anticoagulant activity of crude venom or its fraction on PPP was determined by Ca-clotting time^11,63^. For the control, 1X PBS instead of venom was added to PPP. One unit of coagulant or anticoagulant activity was defined as a decrease or an increase of 1 second of clotting time of PPP incubated with crude ECV or GF fractions, compared to control PPP^63^. Prothrombin time (PT) and activated partial thromboplastin time (APTT) of crude ECV (3 µg/ml) and GF peaks (0.5 µg/ml) were determined using commercial diagnostic kits following the instructions of the manufacturer^63^.

The prothrombin activation (FXa-like activity) property of crude ECV and GF peaks, if any, was analyzed by 12.5% SDS-PAGE of human prothrombin incubated with ECV or fractions or FXa (control)^42^. Formation of thrombin from prothrombin was quantified by measuring the density/intensity of thrombin (36 kDa) band in Image Quant TL software 8.1 (GE Healthcare, Sweden)^49^. Presence of thrombin-like enzyme activity was ascertained by measuring the fibrinogen clotting activity of crude ECV (3 μg/ml) and the GF peaks (0.5 μg/ml)^48^. Loss of platelet integrity induced by crude ECV or GF peaks was determined by incubation of crude ECV (3 µg/ml) or GF peaks (0.5 µg/ml) with washed platelet or Tyrode’s solution (control) at 37 °C for 6 hour in a CO_2_ incubator. The platelets were stained with trypan blue and counted in the hemocytometer using Motic Images plus 3.0 ML software^12^.

### Immunological cross-reactivity, antivenomics, neutralization of enzymatic activities, and tested pharmacological properties of crude ECV by commercial polyvalent antivenom

The immunological cross-reactivity of commercial PAVs towards ECV and its GF fractions was studied by ELISA and western blot analysis, as described previously^11,12^. Briefly, for ELISA experiment, 100 ng protein of ECV or GF fractions was coated in 96 well microtiter ELISA plate (in triplicate) for overnight at 4 °C and washed three times with 1X PBS buffer containing 0.05% tween-20 (wash buffer). Two hundred ng of PAV was used as primary antibody and incubated for 2 h at room temperature followed by washing with wash buffer. Anti-horse IgG HRP conjugated secondary antibody (1:2000 dilution) was incubated for 2 h at room temperature to detect the primary antibody. Color was developed by adding substrate (1X TMB/H_2_O_2_) to the well for 30 minutes in dark condition and reaction was stopped by adding 50 µl of 2 M H_2_SO_4._ The absorbance was taken at 492 nm against blanks in Multiskan GO (Thermoscientific, USA) microplate reader.

Western blotting experiment was performed by running 100 µg protein of ECV (reduced) in 12.5% SDS-PAGE and transferred to PVDF membranes^12^. Non-specific bindings were blocked by 5% fat free skimmed milk for overnight at 4 °C. After that the membranes were washed with 1X TBS with 0.05% tween-20 (washing buffer). Primary antibodies (15 mg/ml PAVs) at a dilution of 1:1000 ratios were incubated for 1 h at room temperature. Thereafter the membranes were washed and ALP conjugated secondary antibody (1:15000 dilution) was incubated for 2 h at room temperature. Blots were developed using BCIP/NBT substrate kit (Sigma Aldrich, USA) and densitometry scanning (Epson America, Inc) was done.

Immunological cross-reactivity of crude ECV against PAV (PSVPL) was assessed by an immunoaffinity chromatographic approach^67^. Briefly, after coupling the NHS-activated Sepharose 4 Fast Flow column (GE Healthcare) with 15 mg PAV (PSVPL), the excess antivenom was washed with 1X PBS pH 7.4 buffer^67^. Then, 500 µg of crude ECV was loaded onto the column and incubated at 37 °C for 2 h in a shaker orbiter. Unbound venom proteins were eluted with 5 column volumes of 1X PBS, pH 7.4, desalted in PD 10 column (GE Healthcare, Sweden) and vacuum dried (Labconco, Model: 7670061, USA). Bound venom proteins were eluted by washing the column with 5 column volumes of 0.1 M glycine, pH 2.0. The pH of the eluted proteins was immediately neutralized by 1 M Tris-Cl, pH 9.0. The bound and unbound fractions, as well as 500 µg of crude ECV were separated by 12.5% SDS-PAGE. The poorly immunogenic, low molecular mass unbound proteins were excised from the gel and subsequently identified by LC-MS/MS analysis against Viperidae protein databases as described above. Percentage of unbound proteins was quantified by spectral count method; the total mean spectral count of PAV unbound proteins was compared with the total mean spectral count of corresponding ECV proteins (100%).

The neutralization potency of polyvalent antivenom (PAV) towards the pro-coagulant activity of crude ECV was determined by pre-incubating PAVs with crude ECV at 1:10 (protein: protein) for 30 min at 37 °C prior to the assay of Ca^2+^ clotting time of PPP, and enzymatic activities of venom^11,12,68^. The percent inhibition was calculated by comparing the pharmacological/enzymatic activity of crude ECV in the absence of PAVs (100% activity).

All the experiments were performed according to Tezpur University ethical committee and bio-safety committee guidelines.

### Data accessibility

The mass spectrometry proteomics data have been deposited to the ProteomeXchange Consortium via the PRIDE^69^ partner repository with Project Name “*Echis carinatus carinatus* (India) venom proteomics” and the dataset identifier PXD007980.

### Statistical Analysis

Student’s t test using Sigma Plot 11.0 for Windows (version 10.0) was used to test the significance of difference in enzymatic activities and pharmacological properties of ECV and its GF fractions. A value of p ≤ 0.05 was considered significant.

## Electronic supplementary material

Supplementary information
