# Supplementary material for: Proteomics and antivenomics of Echis carinatus carinatus venom: Correlation with pharmacological properties and pathophysiology of envenomation
Source: Sci Rep. 2017 Dec 7;7:17119. doi: 10.1038/s41598-017-17227-y (PMC5719401; doi:10.1038/s41598-017-17227-y)
Supplement: Supplementary file 1 — Supplementary information [file 41598_2017_17227_MOESM1_ESM.docx]

**Supporting info**

**Proteomics and antivenomics of *Echis carinatus carinatus* venom: Correlation with pharmacological properties and pathophysiology of envenomation**

**Aparup Patra, Bhargab Kalita, Abhishek Chanda, Ashis K. Mukherjee***

Microbial Biotechnology and Protein Research Laboratory, Department of Molecular Biology and Biotechnology, Tezpur University, Tezpur 784028, Assam, India

*Corresponding author: Dr. A. K. Mukherjee, Department of Molecular Biology and Biotechnology, Tezpur University, Tezpur 784028, Assam, India. Ph: +913712275405; E-mail: [akm@tezu.ernet.in](mailto:akm@tezu.ernet.in)

**Contents:**

1. Supplementary figures S1a-h, S2a-d, S3, S4, S5
2. Supplementary table S1, S2a,b, S3a,b, S4a,b

**Supplementary Fig S1.** Relative abundance of ECV protein families when the LC-MS/MS data was searched against **a & b.** Viperidae database, **c & d.** *Echis* database, **e & f.** *Echis carinatus* database and **g & h.** Transcriptome Shotgun Assembly (TSA) sequences of *Echis coloratus*. The relative abundances in figures **a, c, e** and **g** are derived from MS1 area based method, whereas, figures **b, d, f** and **h** are derived from MS2 spectral count method.

**S1b**

**S1a**

**S1d**

**S1c**

**S1f**

**S1e**

**S1h**

**S1g**

**Supplementary Fig S2a.** Alignment of tryptic and semi-tryptic peptide sequences with Viperidae proteins from NCBI database. The protein alignment was done using Clustal Omega programme (<https://www.ebi.ac.uk/Tools/msa/clustalo/>). The number of proteins in each protein classes is shown in parenthesis. The distinct peptides obtained for each of the following proteins has been highlighted in black or blue (two colours have been used in case of adjacent distinct/unique peptides) or green (for overlapping regions of distinct/unique peptides). The amino acid substitutions within the unique/distinct peptides obtained from MS/MS are highlighted in yellow colour. The LC-MS/MS identified peptides other than unique/distinct peptides are shown in blue colour.

**SVMP (24 proteins)**

gi|297593950 IILESGNVNDYEVVYPQKVTALPKGGIQQPEQKYEDAMQYEFKVKGEPVVLHLEKNKELFSEDYSETHYSPDDREITTNPSVEDH

gi|300079900 IILESGNVNDYEVVYPQKVTAMPKGAVKQPEQKYEDTMQYEFEVNGEPVVLHLEKNKILFSEDYSETHYYPDGREITTNPPVEDH

gi|297593790 ---------------------------KQPEQKYEDTMQYEFKVKGEAVVLHLEKNKGLFSEDYTETHYAPDGREITTKPAVEDH

gi|162329887 -------------------------------------------------------------------------------------

gi|297593842 -------------------------------------------------------------------------------------

gi|297593820 -------------------------------------------------------------------------------------

gi|297593794 ITLESGKINDYEVVYPEKVTALPKGAIQQTEQKYEDAMQYEVKVKGEPVVLHLKKNKDLFSEDYSETHYTPDGREITTNPPIEDH

gi|297593836 -------------------------------------------------------------------------------------

gi|727360735 -------------------------------------------------------------------------------------

gi|297593822 -------------------------------------------------------------------------------------

gi|83523634 IILESGNVNDYEIVYPQKVTAMPIEAILQPEQKYEDVMQYEFEVNGEPVVLHLEKNKDLFSEDYSETYYTPDGREITTNLPVEDH

gi|320579375 ------------------------------------AMQYEFEVNGEPVVLHLEKNKNLFTKDYSETHYSPDGREITTKPLIEDH

gi|297593984 -------------------------------------------------------------------------------------

gi|297594086 -------------------------------------------------------------------------------------

gi|297594068 ------------------------------------------------------------------------------------

gi|297594078 KTLESWNVNDYKVVNPQRVTGL------QPEKKYEDTMQYEFEVNGEPVVLHLEKNKGLFSEDYSETHYSPDGREITTNPPVEDH

gi|297593854 IILESGSVNDYEVVYPQKIIALPKGAVQQPQQKYEDAMQYEFKVKGEPVVLHLEKNKYLFSEDYSETHYSPDGREITTNPPVEDH

gi|297593858 IILESGNVNDYEVVYPQKVTALAKGAIQQPEQKYEDTMQYEFKVNGEPVVLHLEKNKGLFSEDYSETHYSPDGREITTNPAVEDH

gi|297594070 ------------------------------------------------------------------------------------

gi|297593852 ------------------------------------------------------------------------------------

gi|297593830 -------------------------------------------------------------------------------------

gi|297593862 ------------------------------------------------------------------------------------H

gi|52000738 -------------------------------------------------------------------------------------

gi|297593798 --------------------------------------------KGEPVVLHLEKNKDLFSEDYSETHYSPDGREITTNPLVKDH

gi|297593950 CYYHGRIQNDADSTASISACNGLKGHFKLRGETYLIEPLKIPDSEAHAVYKYENVEKEDEAPKMCGVTQTNWE----SDEPIKKAS----------------GLI

gi|300079900 CYYHGHIQNDGHSSASISACNGLKGHFKLRGEMYFIEPLKLSNNEAHAVYKYENIEKEDETPKMCGVTQTNWE----SDKPIKKAS----------------QLV

gi|297593790 CYYHGRIQNDADSSASISACNGLKGHFKLRGEMYFIEPLKIPDSEAHAVYKYENIEKEDEAPKICGVKKTNWE----SDKSIQEAS----------------QLN

gi|162329887 -------------------------------------------------------------------------------------------------------LV

gi|297593842 ---------------------------------------------------------------------------------------------------------

gi|297593820 ---------------------------------------------------------------------------------------------------------

gi|297593794 CYYHGSIQNDAHSSASISACNGLKGHFRLRGETYFIEPLKIPDSEAHAVYKYENIEKEDEAPKMCGVTHTNWE----SDEPIEKAS----------------QLV

gi|297593836 ---------------------------------------------------------------------------------------------------------

gi|727360735 --------------------------------------------------------------------QTNWE----SDEPFKAS-----------------QLN

gi|297593822 ---------------------------------------------------------------------------------------------------------

gi|83523634 CYYHGRLQNDAHSSAIISACNGLKGHFKLRGETYLIEPLKVPDSEAHAVYKYENIEKEDEAPKMCGVTQTNWE----SDEP-----------I-K-E-A-S-Q-L

gi|320579375 CYYHGRIQNDAQSTASISACNGLKGHFKLQGEMYLIEPLKIPDSEAHAVYKYENIEKEDEALKMCGVKHTNWE----SDEPIKEAS----------------QLF

gi|297593984 -----------------------------------------------------------------------WE----SDEPIKKAS----------------QLV

gi|297594086 ---------------------------------------------------------------------------------------------------------

gi|297594068 ---------------------------------------------------------------------------------------------------------

gi|297594078 CYYRGRIQNDADSTASISTCNGLKGFFMLRGETYLIEPLKIPDSEAHAVYKYENVEXEDEAPKMCGVTHTNWE----SDEPIEKAS----------------QLG

gi|297593854 CYYHGHIQNDADSTASISACNGLKGHFKLRGETYFIEPLKVPDSEAHAVYKYENIEKEDEAPKMCGVTQTNWE----SDEPIKKAS----------------QLV

gi|297593858 CYYHGRIQNDADSTASISACNGLKGHFMLRGETYLIEPLKIPDSEAHAVYKYENIEKEDEAPKMCGVTHTNWE----SDEPIKKAS----------------QLV

gi|297594070 ---------------------------------------------------------------------------------------------------------

gi|297593852 ----GRIQNDADSTASISACNGLKGHFKLRGETYFIEPLKIPDSEAHAVYKYENIEKEDEPPKMCGAIHSNWE----SDESIKMAS----------------QLV

gi|297593830 ---------------------------------------------------------------------------------------------------------

gi|297593862 CYYHGHIQNDADSFASISACNGLKGHFKLRGEMYFIEPLKIPDSEAHAVYKYENVEKEDEAPKMCGVTQTNWE----SDEPIKEAS----------------QLN

gi|52000738 ---------------------------------------------------------------------------------------------------------

gi|297593798 CYYHGRIQNDADSTASISACNGLKGHFKLRGETYFIEPLKIPNSEAHAVYKYENIEKEDEAPKMCGVTQDNWE----SDEP-----------I-E-K-A-S-Q-L

gi|297593950 V----------------PPRKRKFRK--LFIELVIVVDHRMVKKHXNDLTAIRTWIYEMVNTVNEMYLPLNIRVPLIGIEFWSNTDLINVTSSADDTLDSFGEWR

gi|300079900 S----------------T--SAQFNK--AFIELIIIVDHSMAKKCNS--TATNTKIYEIVNSANEIFNPLNIHVTLIGVEFWCDRDLINVTSSADETLDSFGEWR

gi|297593790 L----------------TPEQQRYLNSEKHIKVAIIADYLIYRKYGRNLFTIRTRIYEIINILNAIYRAFHMHVALVFLEIWSNGDKINVLPAANVTLDLFGKWR

gi|162329887 S----------------T--SAQFNK--IFIELVIIVDHSMAKKCNS--TATNTKIYEIVNSANEIFNPLNIHVTLIGVEFWCDRDLINVTSSADETLNSFGEWR

gi|297593842 ---------------------------------------------------------------------------------------------------------

gi|297593820 ---------------------------------------------------------------------------------------------------------

gi|297593794 A----------------TSAQHSYYDHFRYVKVIVVVDHSMCTKYNNDLTAMRTRVYEMINTVNEIYIYMHIRVPLVGIEFWCNGDLINVTSSAEYTLNSFGNWR

gi|297593836 ------------------------------------------------------------------------HIALIYLEIWSEGDLINVQSVVDITLDSFGEWR

gi|727360735 L----------------TPEQRTYLKSKKYIELVIVADYIMFWKYDHDLSTIRTRIYEIVNTLNVIYRVLNIYVALVGLEIWCKGNLINVTSSAYDTLDSFGEWR

gi|297593822 -----------------------------------------------------------------------LRVPLVGMEFWNQRDLINVTSSPKVTLDLFGEWR

gi|83523634 -F-A-T------------SEQKSYYDRFRHIELVIVVDHRMVEKYNGNLRKIRRRIYQIVNILNQIYIFMNIRVPLVGVEFWTNGDLINVTSEAEATLNSFGEWR

gi|320579375 A----------------TSEQRRFRE--RYVEFIIVVDQRMYNKHNNDSAAIRTWIFEMLNTVNEIYLPWNIHVPLVGLEFWTQGDLINVVSSADKTLDSFGEWR

gi|297593984 ----------------ATSEQQRYYDRYRYFKIVIVADYRMVTKYNGNLEKIRTRIYQIVNILNEIYRYLYIRVALVGLEIWSSGDLIDVKSASNVTLASFGKWR

gi|297594086 ---------------------------------------------------------------------------------------------------------

gi|297594068 ---------------------------------------------------------------------------------------------------------

gi|297594078 A----------------SSEQQRFDP--RYIGLVIIADHTMYEKYNRNLTAIISRIHQIVNDMIVMYEDLNIYITLAALEIWSKRDLINVVSSSRDTLMSFAEWR

gi|297593854 -V-A-T------------SEQQRYYDRFRHIKLVIVADYRMFRRYNGNLRKIRRKIYQIVNILNEMYLPWNIRVPLVGLEIWNQRDLINVTSSPKVTLNLFGEWR

gi|297593858 A----------------TSEQQRSYK--KYIELVVVADYIMFRKYDRNSTAIKTRIYEIVNTLNLAYIVFSIHVALTHIEIWSKKDQIKVQSAADVTLHLFGDWR

gi|297594070 --------------------------------------------------------------------DLNIYITLAALEIWTERDLINVVSSSSDTLRSFAEWR

gi|297593852 TT------------SEASLIQQTYLDAKKYIELVVVADHEMFRKYNNDSTSVRTRIYEIANIINEMYIPLNIRVALVLLEIWSNRDLIPVQSATTVTLESFGKWR

gi|297593830 -------------------------------------------------------------------------------------------FTADDTMDSFGEWR

gi|297593862 L----------------TPEQQRYLNSQKYIKVAIVADYIMFRKYGRNLFTIRARIYEIVNILNEIYRAFNIHVALVFLEIWSNGDKINVLPEAKVTLDLFGEWR

gi|52000738 ---------------------------------------------------------------------------------------------------------

gi|297593798 -V-A-T------------SEQQTYL--------------------------------------------------------------------------------

gi|297593950 GSDLLNRKRHDHAQLLTDMAFDLNTLGITFFAGMCQAYRSVGLVQDHSTTNLRIAVAMAHEMGHNLGMSHDKKYCTCEDYPCIMSAVLS-----PSRLFSNCSYQ

gi|300079900 ASDLMTRKSHDNALLFTDMRFDLNTLGITFLAGMCQAYRSVGIVQVQGNRNFKTAVIMAHELSHNLGMYHDGKNCICNDSSCVMSPVLS---DQPSKLFSNCSIH

gi|297593790 LSDLLNRREHDNAQLLTGINFDGPTAGLGYVGSMCEPQYSAAIVQDHNKINILVAMAMAHELGHNLGMNHDEKFCTCGAKSCIMSGTLS---CEGSFRFSNCSQE

gi|162329887 ASDLMTRKSHDNALLFTDMRFDLNTLGITFLAGMCQAYRSVGIVQEQGNRNFKTAVIMAHELSHNLGMYHDGKNCICNDSSCVMSPVLS---DQPSKLFSNCSIH

gi|297593842 ---------------------------------------------------------------------------------CIMYPYLS---EPLPMFFSNCSKD

gi|297593820 -------------------------------------------------------------MGHSLGMLHDTKSCTCGANPCIMFSEVS---EPTPKEFSRCSYD

gi|297593794 ASGLLSHKTHDNAQLLTAIDISGSVIGKAHISSMCQATRSVGVVEDHSPIVREVAVTMAHEMGHNLGMYHDGNQCTCGASSCIMSRFLT---DPPPMYFSNCSWN

gi|297593836 KRYLLNRKDHDNAQLLTGINLNGNTIGYGYVGSMCMPKESVGIVQDHSKTYLSVAITMAHELGHNLGINHDKDSCTCQASSCIMAATIS---DQPSYQFSDCSKN

gi|727360735 EKDLLNRKRHDNAQLLTGIDFSGAAAGRGYVGRMCQPKYSVGIVQDHNKIYLLVASAMAHEMGHNLGMDHDGIHCTCGAKSCIMSGILR---CETSYLFSDCSRE

gi|297593822 ESDLLNRKEHDYAQLLTAIDFDGPTIGMAHIATMCHSKLSVGIVEDYRPAESVVAAIMAHEMGHNLGLIHDENQCNCDADSCIMYHQIN---NPPPMYFSNCSWN

gi|83523634 ASYLLSRKNHDHAQLFTAIDLEGLTIGMAPMAGMCQSERSVGIFEDYSPVDHVVAVIIAHEMGHNLGIFHDVHQCNCGANSCIMYAKIS---NPPPMYFSDCSQE

gi|320579375 RKDLLNRKAHDNAHLITAMHFDAQTLGLAYTGSMCHPKYSTGVFQDSSEINIFVAITLAHELGHNLGISHDVPSCTCQTKACIMSPYLS---DQPTKLFSNCSEI

gi|297593984 ESDLLNRIKHDNTQLLTAIHFDGSTIGRAYIYGICDSKLSVGVVEDYSSIERAVATTMAHEMGHNLGMRHDGNQCNCGANSCIMASEMS---DPPPMYFSDCSWN

gi|297594086 -----------------------------------------------------------------------------------MIQVLLVIICLAVFPYQGSSII

gi|297594068 -----------------------------------------------------------------------------------MIQVLLVIICLAVFPYQGSSII

gi|297594078 ERDLLNRRPHDNAQFLTNINFDDNVIGRAYERSMCDPKMSVGIVKDHSTVHLYVASTMAHEMGHNLGMDHDGSQCNCGTAGCVMSATIT---LSRSYQFSDCSKN

gi|297593854 ESDLLSRKRHDNAQLLTAIDFDGLTIGMAHIATMCHSKLSVGIVEDYRPIERVVAAIMAHEMGHNLGIHHDGNQCNCGANSCIMAPQIS---DPPPVYFSNCSWD

gi|297593858 EKNLLTRKKHDNAQLLTGINFKGQTLGRAPVSGMCSPKSSVGVIQDYCKNYFLVAFTMAHELGHNLGMDHDNGSCNCPDKSCIMSAVAG---PEPFFSFSDCSWN

gi|297594070 ERDLLNRRPHDHAQFLTNINFDDDVIGIAYNRGMCDPKKSVGTVMDHSTEHLSVAVAMAHEMGHNLGMDHDGNQCNCGGAGCVMSEELI---ESRSYKFSDCSKN

gi|297593852 ETVLLNRKNHDNAQLLTGIGFNGLTIGKGYVSRMCDPKYSVGIVQDHSTVNLLVASTMAHELGHNLGMSHDTSSCTCYANSCIMSAVLR---NPASRLFSDCSQA

gi|297593830 VSDLLNRKKHDYVQLLTNITLDFKSLGMAFVDGMCKPYRSVGLIKDNS-TTFKTAVIMAHEMGHSLGMHHDSRSCNCAAYPCIMSPVLG---KKPSNKFSSCSYD

gi|297593862 QRDLLNRKKHDNAQLLTGINFDGPTAGLGYVGSLCHPQYSAAIVQDHNKINFLVALAMAHELGHNLGMTHDEXFCTCGAKSCIMSGTLS---CEGSYRFSNCSQE

gi|52000738 ---------------------------------------------------------------------------------------------------------

gi|297593798 ---------------------------------------------------------------------------------------------------------

gi|297593950 DYQKYLLKYKPICMLDPPFRKDVVSPPVC-----GN-ELW-EEG-EECD-CGSP-ADCQN-PCCDA----ATCKLKPGAECGNGMCCDKCKIMTAGTVCRKARHE

gi|300079900 DYQRYLTRYKPKCILYPPLRKDIVSPPVC-----GN-EIW-EEG-EECD-CGSP-ADCQN-PCCDA----ATCKLKPGAECGNGLCCYQCKIKTAGTVCRRARNE

gi|297593790 ENRKYLIRKMPQCILKKPLKTDIVSPPVC-----GN-YLV-ELG-EDCD-CGTP-TFCQN-PCCNA----ATCKLTPGSQCADGECCDQCRFRRAGTECRPAKDE

gi|162329887 DYQRYLTRYKPKCIFNPPLRKDIVSPPVC-----GN-EIW-EEG-EECD-CGSP-ANCQN-PCCDA----ATCKLKPGAECGNGLCCYQCKIKTAGTVCRRARDE

gi|297593842 QYQKFLTNFKPDCTLIRPSRTDIVSPPVC-----GN-DLL-ERG-EECD-CGSP-ENCEN-PCCDA----ASCKLHSWIECEFGECCEQCRLKPAGTECRGIRNE

gi|297593820 QYRDYLPKYNPKCIFDPPLRNDIVSPAVC-----GN-EIW-EEG-EECD-CGSP-ADCEN-SCCDA----ATCKLKPGAECGNGECCDKCKIRTAGTECRAARDD

gi|297593794 YYQNFLTNDKPDCTRIRPSRTDIVSPQVC-----GN-GLL-EKG-EECD-CGSP-ANCRY-PCCDA----ASCKLHSWVECESGECCDQCRFKPAGTECRAIRSE

gi|297593836 ELWGYFISHTPRCILNEPLRTDVVSPAVC-----GN-YVV-EEG-EECD-CGSL-WYCRN-PCCDA----TTCKLKPGAECGEGMCCHQCRFATAETVCRPAKSE

gi|727360735 AHRKYLINNMPQCILNKPLKTDIVSPPVC-----GN-YFV-EVG-EECD-CGSP-RNCQD-QCCDA----ATCKLRPGAQCGEGVCCYQCKFKRAGTVCRPANGE

gi|297593822 YYQNFLTNYKPDCTLIRPSRTDIVSPPVC-----GN-ALL-EKG-EECD-CGSP-ANCQD-PCCDA----ASCKLHSWVECEIGECCDQCRFKPAGTECRAIRSE

gi|83523634 QYQFFLDNYKPDCTLIRPPRTDIVSPPVC-----GN-DLL-EKG-EECD-CGSP-ENCQN-PCCDA----ASCKLHSWIECEFGECCEQCRFKPAGTECRGIRNE

gi|320579375 QYERFLTQYNPKCMINKPLRTDIISPPVC-----GN-DLL-EKG-EECD-CGSP-ENCRY-PCCDA----ASCKLHSWVECESGECCDQCRFRPAGTECRGIRSE

gi|297593984 QYQDYITNYNPDCILIRPLTTDIVSPPVC-----GN-ELL-EEG-EECD-CGSR-ANCRN-PCCDA----ASCKLHSWAECESGECCQQCRFKPAGTECRWRRSE

gi|297594086 LESGNINDYE----IVYPKKVAV-----------LP-TGA---------------MNSVH-PCCDP----VTCEPREGEHCISGPCCRNCKFLNAGTICKKAMLD

gi|297594068 LESGNINDYE----IVYPKKVNV-----------LP-TGA---------------MNSAH-PCCDP----VTCQPREGEHCISGPCCRNCKFLNAGTICKRAVGD

gi|297594078 QYQNYLTNYNPQCILNQPLRTDTVSTPVS-----GN-ELL---------------QNSAN-PCCDP----ETCQPKRGKHCISGPCCRNCYFLRAGTVCKRAVGD

gi|297593854 QYQNFLTNFKPDCTLIRPSTTDIVSPPVC-----GN-GLL-EKR-EECD-CGSP-ENCQD-PCCDA----ALCKLHSWVECEIGECCDQCRFKPAGTECRGIRSE

gi|297593858 DYRRFRNSDQSKCIDNKPLKTDIVSPSVC-----GN-YFV-EVG-EECD-CGSP-TYCQN-PCCDA----ATCKLKPGAECGDGMCCDQCRFRPAGXECRGTSSD

gi|297594070 QYQNYLTIYKPQCILNQPLRTDTVSTPVS-----GN-ELL---------------QNSAN-PCYDP----LTCHPREGEECESGPCCRNCKFLKEGTICKRARGD

gi|297593852 QFRAYLINYTPQCILNEPLSTDIVSPPVC-----GN-ELL-EVG-EECD-CGSP-ANCQD-PLCDA----ASCKLHLWVECESGECCQQGRFKSAQTECRTARSE

gi|297593830 YYKEYLLKYKPKCILDPPLRKDIASPAVC-----GN-EIW-EEG-EECD-CGSP-EDCQN-PCCEA----ETCELYPGAVCEDGLCCDKCKFRTAGTECRKASDE

gi|297593862 ENRRYFINKMPQCILNKPLKTDIVSPAVC-----GN-YLV-EVG-EDCD-CGSP-ANCHN-PCCNA----ATCXLTPGSQCAEGECCDQCRFTRAGTECRPARDE

gi|52000738 ------------------LGTDIISPPVC-----GN-ELL-EVG-EECD-CGFP-RNCRD-PCCDA----TTCKLHSWVECESGECCGQCKFTSAGNECRPARSE

gi|297593798 ----------------EPLGTDIVSPAVC-----GN-ELL-EVG-EECD-CGSP-ANCRD-PHCDA----ALCKLHLGVECESGECCQQGRFMSAQTECRPTRSE

gi|297593950 CDLPEHCTGQSAECPIDEFQRNGEPCLNNLGYCYNGDCPIMTNQCISLFGS----RATVAEDSCFEENLKGSYYGYCKKEN---GRKIPCAPQDVKCGRLYCLDN

gi|300079900 CDVPEHCTGQSAECPRDQLQQNGQPCQNNRGYCYNGDCPIMRNQCISLFGS----RATVAKDSCFQENLKGSYYGYCRKEN---GRKIPCAPQDVKCGRLFCLNN

gi|297593790 CDMADLCNGQSDECPKDQFQRNGHPCQNNNGYCYNGKCPVMGNQCISLFGS----RATVAEDACFQFNRLGSDYGYCRKEN---GIKIPCAPEDVKCGRLYCFDN

gi|162329887 CDVPEHCTGQSAECPRDQLQQNGKPCQNNRGYCYNGDCPIMRNQCISLFGS----RANVAKDSCFQENLKGSYYGYCRKEN---GRKIPCAPQDVKCGRLFCLNN

gi|297593842 CDLPEYCTGQSGDCPTDHFHRNGKPCLYNNGYCYNGACPIMEYQCYDHFGP----DAVVSQDACFEKNKEGKGDFYCRNEN---YVKTPCAPEDVKCGRLFCDIG

gi|297593820 CDVPEHCTGQSAECPRNEFQRNGQPCLNNSGYCYNGDCPIMKNQCILLFSP----NATVDVDACFQWNLRGIFDGYCTKEIGSYGRRFPCAPQDVKCGRLYCLDK

gi|297593794 CDLPESCTGQSAECPTDHFHKNGKPCLNNYGYCYNGKCPIMEYQCYNHFGS----NATVGQDGCFEMNKRGERDFYCRKEN---DVPVPCAQEDVKCGRLFCETE

gi|297593836 CDMAEYCTGRSADCPTDYFHRNGQPCLLNHGYCYNGTCPIMIHQCIILWGT----GATVSPDICFQENNKGQGYFYCRREN---NKNIPCALRDVKCGRLFCKLP

gi|727360735 CDVSDHCTGQSAECPTDHFQKNGQPCLLNRGYCYNGRCPIMIHQCIILWGP----GTTVSPDICFQENNKGQGYFYCRREN---NKNIPCAPQDVKCGRLFCKLP

gi|297593822 CDLPEYCTGQSVDCPIDRFHRNGKPCQSNNGYCYNGACPIMQNQCYALFGP----DATVGQDACFEENKKGKSYFYCRKEN---DAKIPCAPEDVKCGRLLC---

gi|83523634 CDLPEYCTGQSAECPIDRSHRNGKPCLNNYGYCYNGTCPIMYHQCYALFGP----KAVVGQDVCFEENKRGESYFYCRKEN---DVKIPCAPEDIKCGRLFCRHD

gi|320579375 CDLPEYCTGQSADCPTDVFHRDGKPCLSNYGYCYNGTCPIMQYQCYAHFGP----NATVGEDVCFEWNKKGKSDFYCRKEN---DVKIPCAPEDVKCGRLFCETK

gi|297593984 CDLPENCTGQSAVCPIDRFHRNGKPCLQNHGYCYSGNCPIMYHQCYALFGP----NVTVGQDACFEKNKDGIGYYYCRKEN---DVKIPCEPEDIKCGRLFCKND

gi|297594086 -GLNDYCTGISSDCPRNRYKGKEDD--------------------------------------------------------------------------------

gi|297594068 -NMNDYCTGISSDCPRNPYKGKYDPMKWPAA--AKGS-VLM----------------------------------------------------------------

gi|297594078 -DMDDYCSGITPDCPRNRYKD------------------------------------------------------------------------------------

gi|297593854 CDLPEYCTGQSVECPIDHFHRNGKPCQNNNGYCYNGTCPIMQNQCYALFGP----DATVGQDACFEGNKKGESYFYCRKEN---DVKIPCAPEDIKCGRLFCEIV

gi|297593858 CDVPEYCTGQSAECPLDVFQRNGQPCQSNNGYCYNGKCPIMTNQCIHLWKP----GVNVAPDACFEYNLQGTYKHHCGSEN---GRYIKCARQDIKCGRLFCVEP

gi|297594070 -DMDDYCNGKTCDCPRNPHKG-------PAT--AKGS-VLM----------------------------------------------------------------

gi|297593852 CDLPEYCTGQSADCPTDHFHRNGQPCLNNHGYCYNGKCPIMYHQCYALFGP----DATVGQDGCFEWNKKAESYFYCRKEN---DVKIPCAPEDIKCGRLFCEII

gi|297593830 CDVPEYCTGQSADCPRNEFQRNGQPCLNNLGYCYNGDCPIMKNQCISLFGS----RATVAEDSCFQENLKGSTHGYCGKEN---GRKIPCAPQDVKCGRLYCLDN

gi|297593862 CDKADLCTGQSAECPADQFQRNGQPCQNNNGYCYNGICPIMRNQCILLFGS----RATVAEDACFQFNSLGSDYGYCRKEN---GRKIPCAPEDVKCGRLYCFDN

gi|52000738 CDIAESCTGQSADCPMDDFHRNGQPCLNNFGYCYNGNCPILYHQCYALFGS----NVYEAEDSCFERNQKGDDDGYCRKEN---GEKIPCAPEDVKCGRLYCKDN

gi|297593798 CDLPEYCTGQSAECPTDHFHRNGQPCLNNQGYCYNGKCPHMYHQCYSLFGS----IATVGQDGCFEWNKKAESYFYCRKEN---DVMIPCAPEDVKCGRLFCEII

gi|297593950 SPGNKNPCKMHYRCMDQHRGMVEPGTKCEDGKVCNSKRQCVDVNTV----------

gi|300079900 SPRNKNPCNMHYSCMDQHKGMVDPGTKCEDGKVCNNKRQCVDVNTAYQSTTGFSQI

gi|297593790 LPEHKNPCQIYYTLRDENKGMVEPGTKCENGKVCI-NGKCVDVNTAY---------

gi|162329887 SPRNKNPCNMHYSCMDQHKGMVDPGTKCEDGKVCNNKRQCVDVNTAYQSTTG----

gi|297593842 ----PNRCIFPY-GD---TGMVNPGTKCGDKKVCX-HRKCIDVNTTY---------

gi|297593820 SARKKKRCKTNYSPDDENKGMVD---------------------------------

gi|297593794 ----PNMCRHPY-GD---EGMVEPGTKCEDKKVCI-NGKCTDVNTAY---------

gi|297593836 ID-NTPLCNYRYSDVALDYGMVDPGTKCGDGMVCNRNRECVNVNTAY---------

gi|727360735 IH-NTHPCNYRYSDVALDYGMVDPGTKCGDGMVCNGNRECV---------------

gi|297593822 --------------------------------------------------------

gi|83523634 ----IYECRYDY-SENPNYGMVEEGTKCGDGKVCS-KRHCVDVTTAY---------

gi|320579375 PNE--------CKHPYGDEGMVDPGTKCEDGKVCS-NGKCVDVTTAYKSTSGFSQI

gi|297593984 ----KKECN--Y-SEDPNYGMVDEGTKCGDGKVCS-NRHCVNVTKAY---------

gi|297594086 --------------------------------------------------------

gi|297594068 --------------------------------------------------------

gi|297594078 --------------------------------------------------------

gi|297593854 ----KNICKYDY-SEDPNYGMVDEGTKCGDGKVCR-NRHCVDVTTAY---------

gi|297593858 STGNTITCQIFRSQDDPDYGMVDIGTKCADGKVCNSNRHCVDVNTAY---------

gi|297594070 --------------------------------------------------------

gi|297593852 ----KNTCKYDY-SEDPNYGMVDEGTKCGDGKVCS-NRHCVDVTTAY---------

gi|297593830 SSRKKESLQDALFRRGSTQG------------------------------------

gi|297593862 LPEHKNPCQIVYTPSDEDKGMVDPGTKCEDGKVCI-NGKCVDVNTAY---------

gi|52000738 SPGPNDSCKTFNSNEDDHKEMVLPGTKCADGKVCS-NGHCVDVASAY---------

gi|297593798 ----KDTCKYDY-SEDPNYGMVDEGTKCGDGKVCS-NRHCVDVNTAY---------

**PLA_2_ (12 proteins)**

gi|163311140 -SVVELGKMIIQETGKSPFPSYTSYGCFCGGGERGPPLDATDRCCLAHSCCYDTLPDCSPKTDRYKYKRENGEIICENSTSCKKRICECDKAVAVCLRKNLNTYN

gi|149243451 -SLIEFGKMILEETGKLAIPSYSSYGCYCGGGGKGTPKDATDRCCFVHDCCYGNLPDCNPKSDRYKYKRVNGAIVCEDGTSCQNRICECDKAAAICFRQNLTTYS

gi|71912229 GSLLEFGKMILEETGKLAIPSYSSYGCYCGWGGKGTPKDATDRCCFVHDCCYGNLPDCNPKSDRYKYKRVNGAIVCEKGTSCENRICECDKAAAICFRQNLNTYS

gi|82096307 GNLYQFGRMIWNRTGKLPILSYGSYGCYCGWGGQGPPKDATDRCCLVHDCCYTRVGDCSPKMTLYSYRFENGDIICDNKDPCKRAVCECDREAAICLGENVNTYD

gi|87130858 GNLFQFAEMIVKMTGKEAVHSYAIYGCYCGWGGQGKPQDATDRCCFVHDCCYGTVNDCNPKMATYSYSFENGDIVCGDNNLCLKTVCECDRAAAICLGQNVNTYD

gi|59727030 GDLIQFEMLIMKVAKRSGMFWYSAYGCYCGWGGQGKPQDPTDRCCFVHDCCYGKVTDCDPKLVSYTYNEENGEVVCGGDDPCKKQVCECDKAAAICFRDNKDTYD

gi|1041577423 GNLFQFEKLIKKMTGKSGMLWYSAYGCYCGWGGQGRPKDATDRCYFVHDCCYGKVTGCNPKMDIYTYSVENGNIVCGGTNPCKKQICECDGAAAICFRDNLKTYD

gi|298351762 -SLLEFGMMILEETGKLAVPFYSSYGCYCGWGGKATPKDATDRCCFVHDCCYGNLPDCNPKSDRYKYKRVNGAIVCEQGTSCENRICECDKAAAICFRRNLNTYS

gi|1041577114 GNLYQFEKLIQKIVGRSGVLWYSAYGCYCGWGGQGRPQDATDRCCFVHDCCYNKVTSCNPKLDIYTYSVKNRDVVCGGTNPCKKQICECNRAAAICFRDNKDTYD

gi|71912223 GNLLQFGRMIFRMTAKNPLSSYSNYGCYCGWGGKGKPQDATDRCCFVHDCCYEKVNDCNPKTATYSYSFENGGIVCGDRDPCKRAVCECDRVAATCFRDNLNTYD

gi|157834128 -NLFQFAEMIVKMTGKNPLSSYSDYGCYCGWGGKGKPQDATDRCCFVHDCCYEKVKSCKPKLSLYSYSFQNGGIVCGDNHSCKRAVCECDRVAATCFRDNLNTYD

gi|81174981 -SLLEFGMMILEETGKLAVPFYSSYGCYCGWGGKGTPKDATDRCCFVHDCCYGNLPDCTPKPDRYKYKRVNGAIVCEQGTSCENRICECDKAAAICFTKNLNTYS

gi|163311140 K-KYTYYPNFWCKGDIEKC

gi|149243451 E-KYELYPDFLCKGKIKC-

gi|71912229 K-KYMLYPDFLCKGELKC-

gi|82096307 K-KYKSYEDCTEEV-QE-C

gi|87130858 K-N----------------

gi|59727030 WKTYWRFPPENCREESEPC

gi|1041577423 SKTYWKYPKKNCKEESEPC

gi|298351762 K-IYMLYPDFLCKGELKC-

gi|1041577114 SKKYWKYPKKNCKEESEPC

gi|71912223 K-KYRKYPPSQCTGTEQC-

gi|157834128 K-KYHNYPPSQCTGTEQC-

gi|81174981 K-IYMLYPDFLCKGELKC-

**SVSP (11 proteins)**

gi|297593766 QKSSELIIGGAECNINDHRFLVALYNYRTRAFLCGGTLINQEWVLSAAHCNRKSLQIKIGMHSKTAPNDDLQTRVPKETYFCLPNKNYTMWDKDIMLIKLNRPVN

gi|13959655 QKSSELVIGGDECNINEHPFPVALHTARSKRFYCAGTLINQEWVLTAARCDRKNIRIILGVHSKNVPNEDQQIRVPKEKFFCLSSKTYTRWDKDIMLIRLKKPVN

gi|297593764 QKSSELVTGGDECNINEHPFLVALHTARSKRFHCTGTLINEQWVLTAARCNRKNIRIKLGVHNKNVRNENEEMRVPAEKVFCVSSKTYTRWDKDIMLIKMKRPVN

gi|298351881 ------VIGGAECDINEHPSLALIYS-TSMRFHCAGTLLNQEWV------------------------------------------SFTMWDKDIMLI-------

gi|293491172 QKSSELVIGGDECNINEHRSLVFLYNAS--GSACGGTLLNREWVLSAAHCDMENMEIYLGLHNLSLPNKDQQKREPRETHFCLPSRNYTLWDKDIMLIKLNRPVK

gi|306756038 QKSSELVIGGDECNINEHRSLVYLYNDS--NFQCGGTLINQEWVLSAAHCDMENMEIYLGVHNLSLPNKDQKRRDPKEKFFCLSSKNYTKWDKDIMLIKLNRPVK

gi|306756034 QKSSELVIGGDECNINEHPFLALMYNSTSMKFHCSGTLLNEEWVLTAAHCDMENMQIYLGVHDKKNPNKDQQTRVPKEMFFCLSNKSYTPWDKDIMLIRLNSPVT

gi|311223824 QKSSELVVGGDECNINEHRSLVFLYN-N--SFGCSGTLINQQWVLSAVHCDMENVRIYLGVHNLTLRNNA-EIRLPEERFFCLSNKNYTKWDKDIMLIKLDRPVK

gi|134129 ------VVGGDECNINEHPFLVALYTSTSSTIHCGGALINREWVLTAAHCDRRNIRIKLGMHSKNIRNEDEQIRVPRGKYFCLNTKFPNGLDKDIMLIRLRRPVT

gi|297593786 QKSSELVIGGAECNINGHRSLAVLHNSS--GLLCSGTLINEEWVLSAAHCDMENMQIYLGYHNFSVPNDDEEIRVPVEKYFCLPRRNYIVWGKDIMLIKLNRSVN

gi|297593736 QKSSELVIGGAECDINEHRSLVLVYNAS--GFFCGGTLINREWVLSAAHCYMKNMRIYLGLHNFSLPNNDQQRRGARETYFCLPSRNYTKWDKDIMLIKLNRPVN

gi|297593766 NSTHIAPISLPSNRPSLGSVCRIMGWGSIIPTNETYPDVPHCTNINLLHYSVCRAAYPWLPVNSRTFCAGVLQGGKDTCKGDSGGPLICNGQFQG---IL-----

gi|13959655 DSTHIVPLSLPSSPPSVGSVCRIMGWGTITTTKVTYPDVPHCANINMFDYSVCRKVYRKLPEKSRTLCAGILQGGIDSCKVDNGGPLICNGQIQG---IV-----

gi|297593764 NSTHIAPLSLPSNPASVGSVCRIMGWGTITTTKVTYPDVPHCANIKIFDYSVCRGAYRKLPEKSRTLCAGVLEGGIDSCKADTGGPLICNGQFQG---IA-----

gi|298351881 ----------------------------------------------------------------RTLCAGVLEGGKDTCLA------------------------

gi|293491172 SSPHIAPISLPSNPPRLRSVCHIMGWGAITSPNETYPDVPHCANINILRYSVCRAAFGRLPAQSRTLCAGILRGGIDTCLGDSGGPLICNGQIQG---IV-----

gi|306756038 TSTHIAPLSLPSSPPSVGSVCRIMGWGTVTSPNETLLDVPHCANINILNYTVCRAASPRLPTQSRTLCAGILQGGIDACKGDSGGPLICNGQIQG---IV-----

gi|306756034 YSTHIAPFSLPSSPPTVGSVCRIMGWGAITSPNETYPDVPHCANIEIYDYSVCRKAYGGLPEKSRTLCAGVLQGGIDTCLADSGGPLICNGQFQG---IV-----

gi|311223824 TSTYIAPLSLPSSPPRVGSVCRIMGWGAITSPNETFPGVTHCANINILPYSVCRAAYKGLPAQSRTLCGGILEGGIGSCMGDSGGPLICNGEMHG---IV-----

gi|134129 YSTHIAPVSLPSRSRGVGSRCRIMGWGKISTTEDTYPDVPHCTNIFIVKHKWCEPLYPWVPADSRTLCAGILKGGRDTCHGDSGGPLICNGQIQG---IV-----

gi|297593786 ISTHIAPLSLPSSPPSLDSVCRIMGWGAITSPNETYSNVPHCANINILHSSVCRAAYGRLPGLSRTLCAGILRGGIDSCKGDSGGPLICNGQFQG---IV-----

gi|297593736 YSTHIAPLHLPSNPPHLGSVCHIMGWGAITSPNETHPDVPHCANINILRYSICRAAYGGLPAQSRTLCAGILGRRIGSCKGDSGGPLICNGQIQG---IV-----

gi|297593766 SWGWTPCAQLLEPALYTKVFDYSDWIQSIIAGNTTASCPP----------------

gi|13959655 SWGGHPCAQPHKPALYTNVFDYTDWIQSIIAGNITATCPP----------------

gi|297593764 SWGGQPCAQPLKPALYT---------------------------------------

gi|298351881 ----HPCAQPLLPAFYTKVFDYIPWIK-----------------------------

gi|293491172 SWGAEVCAKPHAPGLYTKVSDYTDWIQSIIAGNTTATCPP----------------

gi|306756038 SWGNHPCAQPLKPGHYTHVFDYTDWIQSIIAGNTTATCPP----------------

gi|306756034 AWGRHPCAQPQLPAFYTKVFDYSDWIQSIIAGNTAATCPS----------------

gi|311223824 AWGDDTCAQPHKPVHYTKVYDYTDWIQSIIAGNTAATCPP----------------

gi|134129 AGGSEPCGQHLKPAVYTKVFDYNNWIQNIIAGNRTVTCPP----------------

gi|297593786 SWGDHPCGQRRKPGIYTKVFDYTDWVQSIIAGNTAVTCPP----------------

gi|297593736 SWGAKVCARPRAPGLYTK--------------------------------------

**LAAO (03 proteins)**

gi|194400545 ADDKNPLEECFREDDYEEFLEIAKNGLKKTSNPKHIVIVGAGMSGLSAAYVLAGAGHKVTVLEASERPGGRVRTHRNVKEGWYANLGPMRVPEKHRIIREYIRKF

gi|727360693 ADDKNPLEECFREADYEEFLEIARNGLKKTSNPKDIVVVGAGMSGLSAAYVLAGAGHKVTVLEASERVGGRVRTHRNTKEGWYANLGPMRIPEKHRIIREYIRKF

gi|1002590400 ADNKNPLEECFRETDYEEFLEIARNGLKATSNPKHVVIVGAGMSGLSAAYVLAGAGHQVTVLEASERAGGRVRTYRNDKEGWYANLGPMRLPEKHRIVREYIRKF

gi|194400545 GLKLNEFVQETENGWYFIKNIRKRVGEVKKDPGLLKYPVKPSEAGKSAGQLYQESLGKAVEELKRTNCSYILNKYDTYSTKEYLIKEGNLSPGAVDMIGDLLNED

gi|727360693 GLELNEFVQETDNGWYFIKNIRKRVGEVKKDPGLLKYPVKPSEAGKSAGQLYQASLKKAVKELKRTNCSYMLNKYDTYSTKEYLIKEANLSPGAVDMIGDLLNED

gi|1002590400 GLQLNEFSQENDNAWHFIKNIRKRVGEVKKDPGVLKYPVKPSEEGKSAGQLFEESLRKVEKELKRTNCSYILNKYDTYSTKEYLIKEGKLSPGAVDMIGDLLNED

gi|194400545 SGYYVSFIESLKHDDIFAYEKRFDEIVGGMDQLPTSMYRAIEES--VHFKARVIKIQQNAEKVTVTYQTTQKNLLLETADYVIVCTTSRAARRITFKPPLPPKKA

gi|727360693 SGYYVSFIESLKHDDIFAYEKRFDEIVGGMDQLPTSMYRAIEKS--VLFKARVTKIQQNAEKVRVTYQTAAKTLSYVTADYVC----------------------

gi|1002590400 SGFYMSFTESMKHDDIFAYEKRFDEIVGGMDQLPTSMYRAIEEK--VRFNTRVIKIQQNAKKVTVTYQNRAKDTSLVTADYVIVCTTRAARRITFRPPLPPKKAS

gi|194400545 HALRSVHYRSGTKIFLTCTKKFWEDDGIQGGKSTTDLPSRFIYYPNHNFTTGVGVIIAYGIGDDANFFQALNLNECADIVFNDLSSIHQLPKKDLQTFCYPSIIQ

gi|727360693 ---------------------------------------------------------------------------------------------------------

gi|1002590400 HALRSVHYRSGTKIFLTCTKKFWEDDGIHGGKSTTDLPSRFIYYPNHNFTSGVGVIIAYGIGDDANFFQALDFKSCADIVMNDLSLIHQLPKKDIQAFCYPSMIQ

gi|194400545 KWSLDKYAMGAITTFTPYQFQHFSEALTAPVGRIFFAGEYTANAHGWIDSTIKSGLTAARDVNRASEL------------

gi|727360693 --------------------------------------------------------------------------------

gi|1002590400 KWSLDKYAMGGITTFTPYQFQHFSEALTAPVGRIYFAGEYTANAHGWIDSTIKSGLTAARDVNRASENP-----------

**PDE (1 protein)**

gi|586829527 LKESVEPQVSCRYRCNETFSKMASGCSCDDKCTERQACCQDYEDTCVLPTQSWSCSKLRCSEKRMANVLCSCSEDCLEKKDCCTDYKSICKGETSWLKDQCASSS

AAQCPSGFEQSPLILFSMDGFRAGYLETWDSLMPNINKLKTCGTHAKYMRAVYPTKTFVNHYTIVTGLYPESHGIIDNNIYDVTLNLNFSLSAPTMTNPAWWGGQ

PIWHTVTYQGLKAATYFWPGSEVKINGSYPTIYKVYNKSIPFEARVTEVLKWLDLPKAERPDFVTLYIEEPDTTGHKFGPVSGEIIMALQMADRTLGMLMEGLKQ

RNLHNCVNLILLADHGMEQISCNRLEYMTDYFDKVDFFMYEGPAPRIRSKNVPKDFYTFDSEGIVRNLTCQKPKQYFKAYLAQWMAVRNKNYNRCNGGTHGYDNE

FKSMQAIFLAHGPGFKGKNEVTSFENIEVYNLMCDLLKLKPAPNNGTHGSLNHLLKNPFYNPSPAKEQTSPLSCPFGPVPSPDVSGCKCSSITDLGKVNERLNLN

NQAKTESEAHNLPYGRPQVLQNHSKYCLLHQAKYISAYSQDVLMPLWSSYTINKSPPTSVPPSASDCLRLDVRIPAAQSQTCSNYQPDLTITPGFLYPPNFGSSN

FEQYDALITSNLVPMFKGFTRLWNYFHGTLLPKYARERNGLNVISGPIFDYNYDGHFDSYDTIKEYVNDTKIPIPTHFFVVLTSCENQINTPLNCPGSLKVHRP

DNSESCADTSPDNLWVEERIQTHTARVRDVELLTGLNFYSGLKQPLPETLQLKTFLPIFVNPVN

**NT (1 protein)**

gi|586829529 AREKVGIIGYTTKETPVLSNPGPYLEFRDEVEELQIHANKLTTLGVNKIIALGHSGFFEDQRIARKVKGVDVVVGGHTNTFLYTGSPPSTEVPAGNYPFMVQSDD

GRQVPVVQAYAFGKYLGYLNVVFNDKGNVIKASGNPILLNKDIPEDQVVKAQVNKMKIQLQNYYSQEIGKTIVYLNGTTQACRFHECNLGNLICDAVIYNNLRHP

DDNEWNHVSMCIVNGGGIRSPIDERANNGIITLEELTSVLPFGGTFDLLQIKGSALKQAFEHSVHRHGQGTGELLQVSGIKVVYDLSQKPGSRVVSLNVLCTKCR

VPTYVPLEMEKTYKVLLPSFLATGGDGYHMLKGDSSNHNSGDLDISIVGDYIKRMEKVFPAVEGRVTFLDGTLFQAQLFLTWGLCISLLFFIL

**GC (1 protein)**

gi|380846517 REDRADWTQEKYSHRPTILNATSILQVTSQTNVSRMWQNDLHPIMIERYPGSPGSYAVRQHIKHRLQGLQAGWLVEEDTFQSHTPYGYRTFSNIISTLNPLAKRH

LVIACHYDSKYFPPQLDGKVFVGATDSAVPCAMMLELARSLDRQLSFLKQSSLPTKADLSLKLIFFDGEEAFVRWSPSDSLYGSRSLAQKMSSTPHPPGARNTYQ

TQGIDLFVLLDLIGARNPVFPVYFLNTARWFGRLEAIEQNLHDLGLLNNYSSERQYFRSNLRQHPVEDDHIPFLRRGVPILHLIPSPFPRVWHVMEDNEENLDKP

TIDNLSKILQIFVLEYLNLG

**ASPro (1 protein)**

gi|109287596 LQRISLKKMPSIRETLQEMGMKVADVLPSLKHRISYLDEGLHNKTASTILTNFRDTQYYGEISIGTPAQIFKVVFDTGSSNLWVPSRQCSPLYSACVSHNRYDSS

ESSTYKPKGTKITLTYAQGYIKGFFSQDIVRVADIPIIQFFTEAIALPSIPFIFARFDGVLGMGYPKQAIGGVIPVFDNIMSEKVLSENVFSVYYSRHSESNTGG

EIILGGSDPSHYTGDFHYVSTSREGYWHVDLKGVSIENKIVLCHDGCTATIDTGTSFISGPASSISVLMETIGATLSDGDYVIDCKKINLLPDITFHLGDMTYSL

SSSTYVLKFSDETECTVAFMAVDIPPPLGPLWLLGATFIKQYYIEFDRQNNRIGFATSF

**APase (1 protein)**

gi|743538216 RDESNPFPWDKIRLPKYVVPVHYDLLIHPNLTTLAFTGLSKIEILITEQTSSIILHSKYLQITKTTIHNTKDSADTAKEMMVLEHPPFEQIALVSAEPLQVGQSY

IISIEYSANLSDSFHGFYKSTYRTLEGETRVLASTQFEATAARMAFPCFDEPALKATFSVKIRRNPKHLALSNMPLVKSININEWLIEDQFDISVKMSTYLVAFI

ISDFKSVTKTTLRGIKVSVYTVPHKIKQAEYALDAAVKLLDFFEGYFGIAYPLPKQDLAAIPDFQSGAMENWGLTTYRESALLYDSEKSSVSRKLAITMVVAHEL

AHQWFGNLVTMEWWNDLWLNEGFAKFMEFVSVRKTHPELKVEDHFLNKYFNAMEVDALNSSHPISTPVEDPAQILEMFDDVSYDKGACILNMLQDYLSPEVFQAG

LVKYLLRFSYQNTKNKDLWDSLSDVCPDVDGSHIQNGVCIRNKERVLNSHWTKAVLHDVQAVMNTWTLQKGFPLVTVTIKGKNVHLQQEHYSKKATFSSSAGSLW

HIPLTYITNKCNDVRRFLLTTKTNDIVLPEEVEWIKFNVGMNGYYIVHYGDHGWDSLIRLLKDNHETISSNDRASLINNAFQLVSAGKLSIEKALDLTLYLKHES

EITPVYQGLNELVPLYKLLEKANISDTDQQLKAYIVNLFKNMIDKQSWDDEGTVSEQMLRTSLLTFACVRKYKPCVDKAQEYFMKWKNSDGTLKLPNNIKMAVYA

VGIQTDENWDFLFSKYQLPEFDTEKNQIEVVLCLSQNKEKLQWLMDQALQGDIIKTQELPSILFSVGRNPYGYQLAWKFLKQNWQKLVKKFDFGSHSLAHMITGI

TNQYSTKEQLAEVKNYFSSMDKKTSELRAVQQAIETIEENIQWMDKNLEKIKAWLQINSKV

**PLB (1 protein)**

gi|727360709 DIHYATVYWLEAEKSFQIQDVLDRNGDAYGYYNDTIQSTGWGILEIKAGYGNQHISNEILMYAAGFLEGYLTASHMSDHFANLFPLMIKNVIIEQKVKDFIQKQD

EWTRQQIKNNMDDPFWRNAGYVIAQLDGLYMGNVEWAKRQKRTPLNDFEINFLNALGDLLDLTTAFDSQLRKSDFLSMPDVSRIYQWDMGHCSALIKVLPGYENI

YFAHSSWFTYAATLRIYKHWDFKITDPQTKTGRASFSSYPGLLVSLDDFYILGSGLIMLQTTNSVFNLFLLKQVVPESLFAWERVRIANMMADSGKTWAQIFEKE

NSGTYNNQYMILDTKKIKLQRSLEDGTLYIIEQIPKLVKYSDQTEVLRHGYWPSYNIPFHKVIYNMSGYTEYVQKLGLEFSYEMAPRAKIFRRDQGKVTDMESMK

HIMRYNNYKEDPYTKHNPCNTICCRQDLSRKTPVPAGCYDSKISDISMAAKFTTYAINGPPVEKDLPVFSWVHFNQTKHQSLPESYNFDFVTMKPVL

**Snaclec (20 proteins)**

gi|758377450 AFCCPSGWSAYDQNCYKVFKEEMNWADAEKFCTEQHKGSHLVSLHNIAEADFVVKKTLAMLK----DGVIWMGLND--VWNECNWGWTDGAKLDYKAWNE--GTN

gi|55670410 ---CPLGWSSFDQHCYKVFEPVKNWTEAEEICMQQHKGSRLASIHSSEEEAFVSKLASKALK----FTSMWIGLNN--PWKDCKWEWSDNARFDYKAWKR--RPY

gi|300490462 --DCPSDWSSHEGHCYKVFKLLKTWEDAEKFCTQQANGWHLASIESVEEANFVAQLASETLTKS--KYHAWIGLRDQSQRQQCSSHWTDGSAVSYETVTK--YTK

gi|2829697 DQDCLSGWSFYEGHCYQLFR-LKTWDEAEKYCN-QWDGGHLVSIESNAKAEFVAQLISRKLPKSAIEDRVWIGLRDRSKREQCGHLWTDNSFVHYEHVVP--PTK

gi|32452854 --MCPPGWSSNGVYCYMLFKEPKTWDEAEKFCNKQGKDGHLLSIESKKEEILVDIVVSENIGKM---YKIWTGLSERSKEQHCSSRWSDGSFFRSYEIAI-RYSE

gi|218526484 DQDCLPGWSFYEGHCYKVFNVKKTWEDAEKFCQKQSNGKHLATIEWLGKANFVAELVT--LMKL--ETHVWIGLRVEDKRQQCSSHWTDGSAVSYENVVH--NTK

gi|38493055 DFDCPSDWTAYDQHCYLAIGEPQNWYEAERFCTEQAKDGHLVSIQSREEGNFVAQLVSGFMHRS--EIYVWIGLRDRREEQQCNPEWNDGSKIIYVNWKEGESKM

gi|300490474 DFDCPSGWSAHDQHCYKAFDEPKRSGDAETFCTEQANSGHLVSIESVEEAEFVAQLISENIKTP--ADYVWIGLRNQRKAQYCISKWTDGSSVIYKNVIERFIKN

gi|727360769 DFECPPEWSSYDLHCYKAFDKPKRSRDAEKFCTEQAKGGHLASIESSEEGDFVAKLISENIKSS--ADYVWIGLWNKRREQYCTSQWTDGSNVIYKNVIERFTKN

gi|40889261 --NCLPDWSVYEGYCYKVFKERMNWADAEKFCTKQHKDGHLVSFRNSKEVDFVISLAFPMLK----NDLVWIGLTD--YWRDCNWEWSDGAQLDYKAWDN--ERH

gi|802148 --NCLPDWSVYEGYCYKVFKERMNWADAEKFCMKQVKDGHLVSFRNSKEVDFMISLAFPMLK----MELVWIGLSD--YWRDCYWEWSDGAQLDYKAWDN--ERH

gi|758377456 DFDCPPDWSAYDQHCYKAFDEPKRSGDAEKFCTEQANGGHLVSIESVEEAEFVAQLVSENIKTS--ADYVWIGLWNQRKAQYCISKWTDGSSVIYKNVIERFIKN

gi|300490472 AFCCPSGWSAYDQNCYKVFTEEMNWADAEKFCTEQHKGSHLLSLHNIAEADFVLKKTLAMLK----DGVIWMGLND--VWNECNWGWTDGAKLDYKAWNE--GTN

gi|998226344 DFECPTEWCPYDQHCYRAFDEPKRSVDAEKFCVEQA--GHLASIESQEEADFVAQLVSENVKSS--PDYVWIGLWNQRKEQYCNKKWTDGSSVIYQNMVERFRKN

gi|300490478 DLDCPSGWSAYDQHCYQAVDEPKSWADAEKFCTEQANSGHLVSIKSVGEANFVAQLASGFMQKD--GIYVWIGLRDRRKEQQCRSEWTDGSKIIYVNWKEGESKM

gi|205275155 --DCLPDWSPYQGHCYRVFNQKMTWADAEKFCTEQANGGHLASFHSSKEVDFMVSLAFPMLK----VDFVWIGMSD--FWRDCEWKWSDGAKLDYKAWNN--ELN

gi|538259843 ------------GHCYKPFSEPKNWADAENFCTQQHAGGHLVSFQSSEEADFVVKLAFQTFG----HSIFWMGLSN--VWNQCNWQWSNAAMLRYKAWAE--ESY

gi|578004418 --DCPSDWSSDEEHCYYVFFLLFTWEEAAKFCTQQANGGHLVSIESVEEAEFVAQLISENIKTS--ADYVWIGLWNQRKAPYCVSKWTDGSSVIYKNVIERFIKN

gi|82174836 --DCSSGWTAYGKHCYKVFDEPKTWEDAEKFCSEQANGGHLVSFRSSKEADFVVTLTAQTKE----SEIVWMGLSK--IWNQCDWGWTNGAKLNYEAWAE-AESY

gi|82175557 DFFCPSGWGSNNGHCYQAFNQRMTWEDAERFCSAQAKGGHLVSIETRAEADFVAHVVAERIETS--FPHVWIGLRDEGKEQQCSSEWSDGSSVSYENWIEAESKT

gi|758377450 CFVFK--IAKNHWSRMDCSKTHNFVCKFRV----

gi|55670410 CTVMVVKPDRIFWFTRGCEKSVSFVCKFLTDPAV

gi|300490462 CFGLNKETKYHEWITLPCGDKNPFICKSWVLH--

gi|2829697 CFVLERQTEFRKWIAVNCEFKFPFVCKAKIPR--

gi|32452854 CFVLEKQSVFRTWVATPCENTFPFMCKYPVPR--

gi|218526484 CFGLDQKTGYRTWVALRCELAYHFICMSRVPR--

gi|38493055 CQGLTKWTNFHDWNNINCEDLYPFVCKFSAV---

gi|300490474 CFGLEKQTDYRTWFNLSCGDDYPFVCKFPPRC--

gi|727360769 CFGLEKKTEYRTWFNLRCGDDYPFVCKFPPQC--

gi|40889261 CFIYK--NTDNQWTRRDCTWTFSFVCKCPA----

gi|802148 CFAAK--TTDNQWMRRKCSGEFYFVCKCPA----

gi|758377456 CFGLEKQTNYRTWFNLSCGDDYPFVCKFPPRC--

gi|300490472 CFVFK--IAKNHWSHMDCSSTHNFVCKFRV----

gi|998226344 CFGLEKESGYRTWLNLCCGDDYPFVCKFPPRC--

gi|300490478 CQGLAKWTYFHKWDYVNCAEHYRFVCKFPPQY--

gi|205275155 CFVSK--TTDNQWLRWDCSRTNNVACKYPL----

gi|538259843 CVYFK--STNNKWRSRACRMMAQFVC--------

gi|578004418 CFGLEKETNYRTWFNLSCGDDYPFVCKSPA----

gi|82174836 CVWFS--STNKEWKSRPCSLFGHFVCKSPAW---

gi|82175557 CLGLELDSNYHKWVNVYCGQRNPFVCEA------

**Disintegrin (6 proteins)**

gi|182705265 ---------------------------NSVNPCCDPQTCKPIEGKHCISGPCCENCYFLRSGTICQRARGD-GNNDYCTGITPDCPRNRYN--------------

gi|82194569 ---------------------------NSVHPCCDPVKCEPREGEHCISGPCCRNCKFLNAGTICKRAMLD-GLHDYCTGVTSDCPRNRYNH-------------

gi|182705262 --------------------------MNSGNPCCDPVTCKPRRGEHCVSGPCCRNCKFLNAGTICKRARGD-DMNDYCTGISPDCPRNPWKG-------------

gi|82203514 IILESGNVNDYEIVYPKKVTVLPTGAMNSAHPCCDPVTCKPKRGEHCISGPCCRNCKFLNAGTICKRGRGD-SLHDYCTGVTPDCPRNPNKGESDELEWSAAATG

gi|544584743 IILESGNINDYEIVYPKKVAVLPTGAMNSVHPCCDPVTCEPREGEHCISGPCCRNCKFLNAGTICKKAMLD-GLNDYCTGISSDCPRNRYKGKEDD---------

gi|110346540 ----------------------------------------------CTTGPCCRQCKLKPAGTTCWRTSV---SSHYCTGRSCEC PSYPGNG------------

gi|182705265 ----

gi|82194569 ----

gi|182705262 ----

gi|82203514 SVLM

gi|544584743 ----

gi|110346540 ----

**VEGF (1 protein)**

gi|48429241 QVRPFPDVYQRSACQARETLVSILQEYPDEISDIFRPSCVAVLRCSGCCTDESLKCTPVGKHTVDMQIMRVNPRTQSSKMEVMKFTEHTACECRPRRKQGEPDGP

gi|48429241 KEKPR

**CRISP (03 proteins)**

gi|190195321 GSVDFDSESPRRPEIQNEIVDLHNSLRRSVTPTASNMLKMEWYPEAAANAERWAFRCILNHSPYNSRVIGGIKCGENIYMSPYPMKWTAIIHEWHKEKKDFVYGQ

gi|803374854 -NVDFDSESPRKPEIQNEIIDLHNSLRRSVNPTASNMLRMEWYPEAAANAERWAFRCTLNHSPRDSRVIDGIKCGENIYMSPYPIKWTAIIHKWHDEKKNFVYGI

gi|1041577503 GSVDFDSESPRKPEIQNQIVDLHNSLRRSVNPTASNMLKMEWYPEAAANAERWAYRCIESHSPRNSRVLGGIKCGENIYMSSIPIKWTEIIHAWHGENKNFKYGI

gi|190195321 GASPANAVVGHYTQIVWYKSYRSGCAAAYCPSSEYNYFYVCQYCPAGNIIGKIATPYTSGPPCGDCPSACDNGLCTNPCSHHDEFTNCKDLVKQ-GCHSNYLKTK

gi|803374854 GASPANAVIGHYTQIVWYKSYRGGCAAAYCPSSAYKYFYVCQYCPAGNIIGKTATPYKSGPPCGDCPSACDNGLCTNPCTREDEFINCNDLVKQ-GCQTDYLKSN

gi|1041577503 GADPPNAVIGHFTQIVWYKSYLVGCAATYCPSSEYSYFYVCQYCPAGNIIGKIATPYKSGPPCGDCPSACVNGLCTNPCKHEDVYSNCNDLMKQLSCQNNNMNSN

gi|190195321 CPASCFCHNEII

gi|803374854 CAASCFCHSEIK

gi|1041577503 CPASCFCHNEIK

**KSPI (3 proteins)**

gi|123913154 QDRPKFCHLPVDSGICRAHIPRFYYNPASNQCQGFIYGGCGGNANNFETRDQCRHTCGGK

gi|159883522 HDRPKFCYLPADPGECMAYIRSFYYDSESKKCKEFIYGGCHGNANNFPTRDKCRQTCRGK

gi|239977245 HDRPKFCYLPADPGECLAHMRSFYYDSESKKCKEFIYGGCHGNANKFPSRDKCRQTCGGK

**NGF (1 protein)**

gi|400499 HPVHNQGEFSVCDSVSVWVANKTTATDMRGNVVTVMVDVNLNNNVYKQYFFETKCKNPNPVPSGCRGIDAKHWNSYCTTTDTFVRALTMERNQASWRFIRINTAC

VCVISRKNDNFG

**Supplementary Fig S2b.** Alignment of tryptic, semi-tryptic and non-tryptic (*de novo*) peptide sequences with snake venom protein families reported in the *Echis* databases. The protein alignment was done using Clustal Omega programme (<https://www.ebi.ac.uk/Tools/msa/clustalo/>). The number of proteins in each protein classes is shown in parenthesis. The distinct peptides obtained for each of the following proteins has been highlighted in black or blue (two colours have been used in case of adjacent distinct/unique peptides) or green (for overlapping regions of distinct/unique peptides). The amino acid substitutions within the unique/distinct peptides obtained from MS/MS are highlighted in yellow colour. The LC-MS/MS identified peptides other than unique/distinct peptides are shown in blue colour

**P-I SVMP (1 protein)**

JAC96597.1 DSAFHPSEFRPAGTECRGTSSDCDVPEYCTGQSAECPADQFQRNGQPCQNNNGYCYNGICPIMRNQCILLFGSRATVAEDACFQFNSLGSDYGYCRKENGRKIPCAPEDVKCGRLYCFDNLPEH

KNPCQIVYTPSDEDKGMVDPGTKCEDGKVCINGKCVDVNTAY

**P-II SVMP (4 proteins)**

ADI47726.1 ------------------------------------------------------------------------------------------------------------------------

ADI47717.1 ------------------------------------------------------------------------------------------------------------------------

ADI47689.1 MIQVLLVTICLAVFPYQGSSKTLKSGNVNDYKVVNPQRVTGLPKGAVKQPEKKYEDGVQYEFEVNGEPVVLHLEKNKGLFSEDYSETHYSPDGREITTNPPVEDHCYYHGHIKNDADSTA

ADI47722.1 MIQVLLVTICLAVFPYQGSSKTLESWNVNDYKVVNPQRVTGL------QPEKKYEDTMQYEFEVNGEPVVLHLEKNKGLFSEDYSETHYSPDGREITTNPPVEDHCYYRGRIQNDADSTA

ADI47726.1 ------------------------------------------------------------------------------------------------------------------------

ADI47717.1 ------------------------------------------------------------------------------------------------------------------------

ADI47689.1 SISACNGLKGFFTLRGETYLIEPLRVXXXXXXXXXXXXXXXXXXXXXXXXXXXXXXXXXX XXXXXXXXXXXXXXXXXXXQHFDPRYIELVIVADHAMVTKNNGDLTAIRTWVHQIVNDMT

ADI47722.1 SISTCNGLKGFFMLRGETYLIEPLKIPDSEAHAVYKYENVEXEDEAPKMC----GVTHTN WESDEPIEKASQLGASSEQQRFDPRYIGLVIIADHTMYEKYNRNLTAIISRIHQIVNDMI

ADI47726.1 ------------------------------------------------------------------------------------------------------------------------

ADI47717.1 ------------------------------------------------------------------------------------------------------------------------

ADI47689.1 VMYRDLNIHITLAAVVIWNKRDLITVTSSAEDTLNLFGTWRETKYLKHRKHDNAQLLTGIILDDDTIGLAYVGGMCDPKKSVGIIEDHSTEHLLVAATMAHEMGHNLGMNHDANQCNCGA

ADI47722.1 VMYEDLNIYITLAALEIWSKRDLINVVSSSRDTLMSFAEWRERDLLNRRPHDNAQFLTNINFDDNVIGRAYERSMCDPKMSVGIVKDHSTVHLYVASTMAHEMGHNLGMDHDGSQCNCGT

ADI47726.1 ----MIQVLLVIICLAVFPYQGSSIILESGNINDYE----IVYPKKVAV------LPTGAMNSVHPCCDPVTCEPREGEHCISGPCCRNCKFLNAGTICKKAMLDGLNDYCTGISSDCPR

ADI47717.1 ----MIQVLLVIICLAVFPYQGSSIILESGNINDYE----IVYPKKVNV------LPTGAMNSAHPCCDPVTCQPREGEHCISGPCCRNCKFLNAGTICKRAVGDNMNDYCTGISSDCPR

ADI47689.1 NGCVMSATLT---EQTSYQFSDCSKDEYQNYLTDHNPQCILNQPLRTD---TPVSRNELLQNSAHPCCDPVTCQPKR--FCVSGPCCENCQFVRAGTVCNPAKGDWMDDYCTGISSDCPR

ADI47722.1 AGCVMSATIT---LSRSYQFSDCSKNQYQNYLTNYNPQCILNQPLRTDTVSTPVSGNELLQNSANPCCDPETCQPKRGKHCISGPCCRNCYFLRAGTVCKRAVGDDMDDYCSGITPDCPR

ADI47726.1 NRYKGKEDD-------------

ADI47717.1 NPYKGKYDPMKWPAAAKGSVLM

ADI47689.1 NPLNGF-------R--------

ADI47722.1 NRYKD-----------------

**P-III SVMP (19 proteins)**

ADI47593.1 ------------------------------------------------------------------------------------------------------------------------

ADI47643.1 MIQILLPTICLAVFPYQGCSIILESGNVNDYEVVYPQKFTALPKGGIQQPEQKYEDAMQYEFKVKGEPVVLHLEKNKELFSEDYSETHYSPDDREITTNPSVEDHCYYHGRIQNDADSTA

ADI47598.1 ------------------------------------------------------------------------------------------------------------------------

ADI47635.1 MIQILLVIICLAVFPYQGCSIILGSGNVNDYEVVYPQKVTALPKGAVQQPEQKYEDAIQYEFEVKGEPVVLHLEKNKGLFSEDYSETHYSSDDREITTKPSVEDHCYYHGRIQNDAESTA

ADI47601.1 ------------------------------------------------------------------------------------------------------------------------

ADW54341.1 MMQVLLVTICLAVFPYQGSSIILESGNVNDYEIVYPQKVTALPIEAILQPEQKYEDAMQYEFEVNGEPVVLHLEKNKNLFTKDYSETHYSPDGREITTKPLIEDHCYYHGRIQNDAQSTA

ADW54346.1 --------------------------------------------------------AMQYEFEVNGEPVVLHLEKNKNLFTKDYSETHYSPDGREITTKPLIEDHCYYHGRIQNDAQSTA

ADI47584.1 MIQPLLVVTCLVVFPYQGSSIILEFGDVNDYEVVYPQKITALSKEAIQQPEQKYEDTMQYEFKVNGEPVILHLEKNKGLFSDNYSETHYSPDGREITTNPPVKDHCYYHGHIQNDAPSSA

ADI47609.1 -------------------------------------------------------------------------------------------------------------GRIQNDADSTA

ADI47604.1 ------------------------------------------------------------------------------------------------------------------------

ANT80539.1 MIQVLLVTVCLAVFPYQGSAIILESGNVNDYEVVYPQKVTALPKGAIQQPQQKYEDTMQYEFKVNGEPVILHMEKNKYLFSEDYSETHYSPDGREITTNPPVEDHCYYHGRIQNDAHSSA

ADI47664.1 ------------------------------------------------------------------------------------------------------------------------

ADI47580.1 MMQVLLATICLAVFPYQGSSITLESGKINDYEVVYPEKVTALPKGAIQQTEQKYEDAMQYEVKVKGEPVVLHLKKNKDLFSEDYSETHYTPDGREITTNPPIEDHCYYHGSIQNDAHSSA

ADI47592.1 MMQVLLATICLAVFPYQGSSIILESGNINDYEVVYPEKVTALPKGAIQQTEQKYEDAMQYEFKVKGEPVVLHLKKNKDLFSEDYSETHYTPDGREITTNPPIEDHCYYHGSIQNDAHSSA

ADI47645.1 MIQVLLVIICLAVFPYQGSSIILESGNVNDYEVVYPQKVTALPKGAIQQPEQKYEDTMQYEFKVNGEPVIFHMEKNKYLFSEDYSETHYSPDGREITTNPPVEDHCYYHGRIKNDADSTA

CAJ01683.1 MMQVLLVTICLAVFPYEGSSIILESGNVNDYEIVYPQKVTAMPIEAILQPEQKYEDVMQYEFEVNGEPVVLHLEKNKDLFSEDYSETYYTPDGREITTNLPVEDHCYYHGRLQNDAHSSA

ADI47583.1 MIQVLLVTICLAVFPYQGGSIILESGNVNDYEVVYPQKVTALPKGAIQQPEQKYEDAMQYEFKVKGEPVVLHLEKNKDLFSEDYSETHYSPDGREITTNPLVKDHCYYHGRIQNDADSTA

ADI47590.1 MMQVLLATICLAVFPYQGSSIILESGNVNDYEVVYPQKVTALPQGAIQQPEQKYEDTMQYEIEVNGEPVVLHLEKNKDLFSEDYSETHYSPDGRKITTNPPVQDHCYYHGRIQNDAHSTA

ADW54348.1 MMQVLLVTICLAVFPYQGSSIILESGNVNDYEVVYPQKVTALPKGAIQQAEQKYEDAMQYEFEVNGQPVVLHLEKNKDLFSEDYSETHYSPDGKEITTNPPIEDHCYYHGRIQNDAHSTA

ADI47593.1 ------------------------------------------------------------------------------------------------------------------------

ADI47643.1 SISACNGLKGHFXXXXXXXXXXXXXXXXXXXXXXXXXXXXXXXXXXXX--XXXXXXXXXXXXXXXXXXXXXXXXXXXXXXXXRKFRKLFIELVIVVDHRMVKKHKNDLTAIRTWIYEMVN

ADI47598.1 ------------------------------------------------------------------------------------------------------------------------

ADI47635.1 SISACNGLKGHFTLRGETYFIEPLKIPDSEAHAVYKYENIEKEDEAPKMCGVXXXXXXXXXXXXXXXXXXXXXXXXXXXXXXXXXXXXXXXXXXXXXXXXXTKYNNDSTAIRTWIYEMLN

ADI47601.1 ------------------------------------------------------------------------------------------------------------------------

ADW54341.1 SISACNGLKGHFKLQGEMYLIEPLKIPDSEAHAVYKYENIEKEDEALKMCGVKHTNWESDEPIKEAS----QLVATS-EQRRFRE--RYVEFIIVVDQRMYNKHNNDSAAIRTWIFEMLN

ADW54346.1 SISACNGLKGHFKLQGEMYLIEPLKIPDSEAHAVYKYENIEKEDEALKMCGVKHTNWESDEPIKEAS----QLFATS-EQRRFRE--RYVEFIIVVDQRMYNKHNNDSAAIRTWIFEMLN

ADI47584.1 SISACNGLKGHFMLRGETYLIEPLKFPDNEAHAVYKYENIEXEDEAPKMCGVTHTNWESDEPIKKAS----PLVATS-EQQRSYE--KYIELALIADNLIYRKYTGNSTAIKTRMYEIIN

ADI47609.1 SISACNGLKGHFKLRGETYFIEPLKIPDSEAHAVYKYENIEKEDEPPKMCGAIHSNWESDESIKMASQLVTTSEASL-IQQTYLDAKKYIELVVVADHEMFRKYNNDSTSVRTRIYEIAN

ADI47604.1 ------------------------------------------------------------------------------------------------------------------------

ANT80539.1 SISACNGLKGHFKLQGETYFIESLKIPDSEAHAVYKYENIEKEDEAPKMCGVTQTNWEPDEPIKEAS----QLVATS-EQRSYYDNFRYVKFFIVVDHSMVTKYNNDLTAIRTRIYEMLN

ADI47664.1 ------------------------------------------------------------------------------------------------------------------------

ADI47580.1 SISACNGLKGHFRLRGETYFIEPLKIPDSEAHAVYKYENIEKEDEAPKMCGVTHTNWESDEPIEKAS----QLVATS-AQHSYYDHFRYVKVIVVVDHSMCTKYNNDLTAMRTRVYEMIN

ADI47592.1 SISVCNGLKGHFRLRGEMYFIEPLKIPDSEAHAVYKYENIEKEDEAPKMCGVTHTNWESDETIEKAS----QLVATS-EQHSYYDYFRYVKVIVVVDHSMCTKYNNDLTAMRTRVYEMFN

ADI47645.1 SISACNGLKGHFKLRGETYFIEPXEVPDSEAHAVYKYENIEKEDEAPKMCGVTQTNWXXXXXXXXXXXXXXXXXXXX-XXHSYYDRFRHIKLVIVVDHSLFRSYNNDSAAVRARIYQIVN

CAJ01683.1 IISACNGLKGHFKLRGETYLIEPLKVPDSEAHAVYKYENIEKEDEAPKMCGVTQTNWESDEPIKEAS----QLFATS-EQKSYYDRFRHIELVIVVDHRMVEKYNGNLRKIRRRIYQIVN

ADI47583.1 SISACNGLKGHFKLRGETYFIEPLKIPNSEAHAVYKYENIEXEDEAPKMCGVTQDNWESDEPIEXAS----QLVATS-EQQTYL------------------------------------

ADI47590.1 SMSACNGLKGYFKLRGETYFIEPLKIPDSEAHAVYKYENLEKEDETPKICGVTQTNWESDEPIKKAS----QLVATS-EQQRYYDRFRYVXXIVVVDHRMCKKYNGNLRRIRRRLYQLIN

ADW54348.1 SISACNGLKGHFKLRGETYLIEPLKIPDSEAHAVYKYENIEKEDDAPKMCGVTQTNWESDEPIKEAS----QLVATS-DQQRYYDHFRYIKYFIVVDHRMVEKYNGNLRTIRRRIYQLVN

ADI47593.1 ---------------------------------------------------------------------------------------------------------MGHSLGMLHDTKSCT

ADI47643.1 TVNEMYLPLNIRVPLIGIEFWSNTDLINVTSSADDTLDSFGEWRGSDLLNRKRHDHAQLLTDMAFDLNTLGITFFAGMCQAYRSVGLVQDHSTTNLRIAVAMAHEMGHNLGMSHDKKYCT

ADI47598.1 ------------------------------FTADDTMDSFGEWRVSDLLNRKKHDYVQLLTNITLDFKSLGMAFVDGMCKPYRSVGLIKDNS-TTFKTAVIMAHEMGHSLGMHHDSRSCN

ADI47635.1 TVNEIYLPLNIRVTLVGLEFWSNRDLINVTFTADDTMDSFGEWRVSDLLNRKRHDYAQLLTNITLDFDSLGMAFIDGMCKSYRSVGLIRDCSNTTFKTAVIMAHEMGHSLGMHHDSKSCK

ADI47601.1 -----------HIALIYLEIWSEGDLINVQSVVDITLDSFGEWRKRYLLNRKDHDNAQLLTGINLNGNTIGYGYVGSMCMPKESVGIVQDHSKTYLSVAITMAHELGHNLGINHDKDSCT

ADW54341.1 TVNEIYLPWNIHVPLVGLEFWTQGDLINVVSSADKTLDSFGEWRRKDLLNRKAHDNAHLITAMHFDAQTLGLAYTGSMCHPKYSTGVFQDSSEINIFVAITLAHELGHNLGISHDVPSCT

ADW54346.1 TVNEIYLPWNIHVPLVGLEFWTQGDLINVVSSADKTLDSFGEWRRKDLLNRKAHDNAHLITAMHFDAQTLGLAYTGSMCHPKYSTGVFQDSSEINIFVAITLAHELGHNLGISHDVPSCT

ADI47584.1 TLNLVYIVFSIHIALTHIEIWSETDQIKVQSAADDTLHLFGDWREKNLMKRKEHDNAQLVTGINFNGQTLGRAPVSGMCSPRRSVGVIQDYCKTHLLTAVVMAHELGHNLGMNHDKSHCK

ADI47609.1 IINEMYIPLNIRVALVLLEIWSNRDLIPVQSATTVTLESFGKWRETVLLNRKNHDNAQLLTGIGFNGLTIGKGYVSRMCDPKYSVGIVQDHSTVNLLVASTMAHELGHNLGMSHDTSSCT

ADI47604.1 ------------------------------------------------------------------------------------------------------------------------

ANT80539.1 TVNEIYLYLHIRVPLVGVEFWTNGDLINVTSKAEDTLYSFGDWRASNLMRRKRHDNAQLFTAIDISNSTIGIAHVSSMCQATRSVGVVEDYSPIVREVAVTMVHEMGHNLGIDHDGKQCH

ADI47664.1 --------------------------------------LFGVWRASDLLSRRRHDNAHLLTAIDLNGPTIGIAHVSRMCQATRSVGVVQDHSPTVRAVAVTMAHEMGHNLGMSHDGNHCN

ADI47580.1 TVNEIYIYMHIRVPLVGIEFWCNGDLINVTSSAEYTLNSFGNWRASGLLSHKTHDNAQLLTAIDISGSVIGKAHISSMCQATRSVGVVEDHSPIVREVAVTMAHEMGHNLGMYHDGNQCT

ADI47592.1 TVNEIYIYMHIHVPLVGIEFWCNGDLITVTSSAENTLYSFGDWRASYLLSHKPHDNAQLLTAIDISNSTIGIAHVSSMCQATRSVGVIEDHSPIVRAVAVTMAHEMGHNLGMYHDGKQCN

ADI47645.1 ILNEIYIYLHIRVALVGIEFWSNGDLINVTSSARDTLKSFGNWRASDLLNRKRHDNAQLLTATDISNSTIGIAYVSSMCQATHSVGVVEDYSPLVHAVAATMAHEMGHNLGMQHDENYCN

CAJ01683.1 ILNQIYIFMNIRVPLVGVEFWTNGDLINVTSEAEATLNSFGEWRASYLLSRKNHDHAQLFTAIDLEGLTIGMAPMAGMCQSERSVGIFEDYSPVDHVVAVIIAHEMGHNLGIFHDVHQCN

ADI47583.1 ------------------------------------------------------------------------------------------------------------------------

ADI47590.1 FLNEMYLPWNLRVPLVGMEFWNQRDLINVTSSPKVTLDLFGEWRESDLLNRKEHDYAQLLTAIDFDGPTIGMAHIATMCHSKLSVGIVEDYRPAESVVAAIMAHEMGHNLGLIHDENQCN

ADW54348.1 ILNEIYLPWNIRAPLVGIEFWNQRDLINVTSSAPYTLDLFGKWRASDLLNRKIHDYTHLLTAIVFVEQILGMAHIATMCHSELSVGLVQDYMPSEHVVAAIMVHEMGHNLGISHDEKYCN

ADI47593.1 CGANPCIMFSEVSEPTPKEFSRCSYDQYRDYLPKYNPKCIFDPPLRNDIVSPAVCGNEIWEEGEECDCGSPADCENSCCDAATCKLKPGAECGNGECCDKCKIRTAGTECRAARDDCDVP

ADI47643.1 CEDYPCIMSAVL--SPSRLFSNCSYQDYQKYLLKYKPKCMLDPPFRKDVVSPPVCGNELWEEGEECDCGSPADCQNPCCDAATCKLKPGAECGNGMCCDKCKIMTAGTVCRKARHECDLP

ADI47598.1 CAAYPCIMSPVLGKKPSNKFSSCSYDYYKEYLLKYKPKCILDPPLRKDIASPAVCGNEIWEEGEECDCGSPEDCQNPCCEAETCELYPGAVCEDGLCCDKCKFRTAGTECRKASDECDVP

ADI47635.1 CVASPCIMSKALGKQPSKVFSSCSYDDYNTYLLKYKPKCILDPPLRKDIASPAVCGNEIWEEGEECDCGSPEDCQNPCCDAETCELYPAAVCEDGPCCHKCKFKTAGTECRPARDECDVA

ADI47601.1 CQASSCIMAATISDQPSYQFSDCSKNELWGYFISHTPRCILNEPLRTDVVSPAVCGNYVVEEGEECDCGSLWYCRNPCCDATTCKLKPGAECGEGMCCHQCRFATAETVCRPAKSECDMA

ADW54341.1 CQTKACIMSPYLSDQPTKLFSNCSEIQYERFLTQYNPKCMINKPLRTDIISPPVCGNELLEREEECDCGSPENCRDPCCDAASCKLHSWVECESGECCDQCRFKRAGTLCRPARDDCDMA

ADW54346.1 CQTKACIMSPYLSDQPTKLFSNCSEIQYERFLTQYNPKCMINKPLRTDIISPPVCGNDLLEKGEECDCGSPENCRYPCCDAASCKLHSWVECESGECCDQCRFRPAGTECRGIRSECDLP

ADI47584.1 CPVKSCIMSPTARPEPVFSFSDCSWNDYRSFRDSDQSKCIDNKPLKTDIVSPSVCGNNFVEVGEECDCGSPKYCRNPCCDAATCKLKPGTECGDGMCCDQCRFKPAGTQCRGTRSDCDVP

ADI47609.1 CYANSCIMSAVLRNPASRLFSDCSQAQFRAYLINYTPQCILNEPLSTDIVSPPVCGNELLEVGEECDCGSPANCQDPLCDAASCKLHLWVECESGECCQQGRFKSAQTECRTARSECDLP

ADI47604.1 -----CIMYPYLSEPLPMFFSNCSKDQYQKFLTNFKPDCTLIRPSRTDIVSPPVCGNDLLERGEECDCGSPENCENPCCDAASCKLHSWIECEFGECCEQCRLKPAGTECRGIRNECDLP

ANT80539.1 CGANSCIMAPQISDPPPMYFSDCSQEQYQKFLTNYKPDCTLIRPSGTDIVSPPVCGNDFLEEGEECDCGSPANCQYPCCNATSCKLHSWVECESGACCDQCRFRPAGTECRRIRSECDLP

ADI47664.1 CGANSCIMAAVLRNPAPEYFSDCSRRYYQNFLTNYIPDCTLIRPSKTDIVSPQVCGNGLLEEGEECDCGSPANCQYPCCDAASCKLHSWVECEFGQCCDQCRFKPAGTECRGIRSECDLP

ADI47580.1 CGASSCIMSRFLTDPPPMYFSNCSWNYYQNFLTNDKPDCTRIRPSRTDIVSPQVCGNGLLEKGEECDCGSPANCRYPCCDAASCKLHSWVECESGECCDQCRFKPAGTECRAIRSECDLP

ADI47592.1 CGADSCIMYPYLSDPLPMYFSNCSWNYYQNFLTNYKPDCTRIKPSRTDIVSPPVCGNGLLEKGEECDCGSPANCQYPCCDAASCKLHSWVECESGECCDQCRFKAAGTECRAIRNECDLP

ADI47645.1 CGPNLCIMASYISDPPPMYFSNCSWNYYQNFLTNYQPDCTLIRPSRTDIVSPPVCGNGLLEQGEECDCGSPANCQYHCCDAASCKLHPWVECESGECCDQCRFRPAGTECRETRNECDLP

CAJ01683.1 CGANSCIMYAKISNPPPMYFSDCSQEQYQFFLDNYKPDCTLIRPPRTDIVSPPVCGNDLLEKGEECDCGSPENCQNPCCDAASCKLHSWIECEFGECCEQCRFKPAGTECRGIRNECDLP

ADI47583.1 ------------------------------------------EPLGTDIVSPAVCGNELLEVGEECDCGSPANCRDPHCDAALCKLHLGVECESGECCQQGRFMSAQTECRPTRSECDLP

ADI47590.1 CDADSCIMYHQINNPPPMYFSNCSWNYYQNFLTNYKPDCTLIRPSRTDIVSPPVCGNALLEKGEECDCGSPANCQDPCCDAASCKLHSWVECEIGECCDQCRFKPAGTECRAIRSECDLP

ADW54348.1 CGADSCIMYPQISIPPPVYFSNCSWEQYQNFLTIYKPDCTLIRPSRTDIVSPPVCGNDILEQGEECDCGSPEKCQDPCCDAASCKLHSWIECXFGECCDQCRFKPAGTECRGIRSECDLP

ADI47593.1 EHCTGQSAECPRNEFQRNGQPCLNNSGYCYNGDCPIMKNQCILLFSPNAT----VDVDACFQWNLRGIFDGYCTKEIGSYGRRFPCAPQDVKCGRLYCLDKSARKKKRCKTNYSPDDENK

ADI47643.1 EHCTGQSAECPIDEFQRNGEPCLNNLGYCYNGDCPIMTNQCISLFGSRAT----VAEDSCFEENLKGSYYGYCKKEN---GRKIPCAPQDVKCGRLYCLDNSPGNKNPCKMHYRCMDQHK

ADI47598.1 EYCTGQSADCPRNEFQRNGQPCLNNLGYCYNGDCPIMKNQCISLFGSRAT----VAEDSCFQENLKGSTHGYCGKEN---GRKIPCAPQDVKCGRLYCLDNSSRKKESLQDALFRRGSTQ

ADI47635.1 EHCTGQSSECPRNELQRNGQPCLKNSGYCYNGDCPIMTNQCISLFGSRAT----VAEDSCFQENLKGSKHGYCAKEN---GRKIPCAPQDVKCGRLYCLDNSSRKKNPCKMHYLDADQHK

ADI47601.1 EYCTGRSADCPTDYFHRNGQPCLLNHGYCYNGTCPIMIHQCIILWGTGAT----VSPDICFQENNKGQGYFYCRREN---NKNIPCALRDVKCGRLFCKLPIDNTPL-CNYRYSDVALDY

ADW54341.1 ESCSGHSADCPIDGFHANGQPCSHNLGYCYNGKCPLTLYQCRAFLGKDVV----GVQESCFQYNRLGNTYAYCRKEN---GRKIPCAPKDEKCGRLYCSYKSFGDYISCLPCYRANEEDK

ADW54346.1 EYCTGQSADCPTDVFHRDGKPCLSNYGYCYNGTCPIMQYQCYAHFGPNAT----VGEDVCFEWNKKGKSDFYCRKEN---DVKIPCAPEDVKCGRLFCETKPNE--------CKHPYGDE

ADI47584.1 EYCTGRSAECPLDVFQRNGQPCRSNNGYCYNGNCPIMTNQCIDLWKPAPLAGVNVAPDRCFDYNLQGTDKYHCGIKN---GRYIKCARQDIKCGRLFCVEPSTGNKITCQSFRSQDDPDY

ADI47609.1 EYCTGQSADCPTDHFHRNGQPCLNNHGYCYNGKCPIMYHQCYALFGPDAT----VGQDGCFEWNKKAESYFYCRKEN---DVKIPCAPEDIKCGRLFCEIIKNTC-----KYDYSEDPNY

ADI47604.1 EYCTGQSGDCPTDHFHRNGKPCLYNNGYCYNGACPIMEYQCYDHFGPDAV----VSQDACFEKNKEGKGDFYCRNEN---YVKTPCAPEDVKCGRLFCDIGPNRC-----IFPYGD---T

ANT80539.1 EYCTGQSAECPKDVFHRDGKPCLNNYGYCYNGKCPIMQYQCYVHFGPNAT----VGEDVCFEWNKKGKSDFYCRKEN---DVEIPCAPEDVKCGGLFCETKPNGC-----KHPYGD---E

ADI47664.1 EYCTGQSAECPTDVFHKDGKPCLNNYGYCYNGTCPIMQYQCYAHFGQNAV----VGQDACFEINKEGKGDFYCRKEN---DVPIPCAQEDVKCGRLFCETEPNMC-----RYPYGD---E

ADI47580.1 ESCTGQSAECPTDHFHKNGKPCLNNYGYCYNGKCPIMEYQCYNHFGSNAT----VGQDGCFEMNKRGERDFYCRKEN---DVPVPCAQEDVKCGRLFCETEPNMC-----RHPYGD---E

ADI47592.1 ESCTGQSGDCPIDQFHRNGQPCLHNYGYCYNGKCPIMEYQCYDHFGSNAV----VGQDACFKWNTKGKSDFYCRKEN---DVKIPCAPEDVKCGRLFCDIGPNRC-----RYPYGD---E

ADI47645.1 ENCTGQSAVCPIDRFHRNGKPCLNNYGYCYNGTCPIMYHQCYALFGPNAV----VGQDVCFEDNKRGESYFYCRKEN---DVPIPCAHEDIKCGRLFCKHDKYEC-----RYDYSENPNY

CAJ01683.1 EYCTGQSAECPIDRSHRNGKPCLNNYGYCYNGTCPIMYHQCYALFGPKAV----VGQDVCFEENKRGESYFYCRKEN---DVKIPCAPEDIKCGRLFCRHDIYEC-----RYDYSENPNY

ADI47583.1 EYCTGQSAECPTDHFHRNGQPCLNNQGYCYNGKCPHMYHQCYSLFGSIAT----VGQDGCFEWNKKAESYFYCRKEN---DVMIPCAPEDVKCGRLFCEIIKDTC-----KYDYSEDPNY

ADI47590.1 EYCTGQSVDCPIDRFHRNGKPCQSNNGYCYNGACPIMQNQCYALFGPDAT----VGQDACFEENKKGKSYFYCRKEN---DVKIPCAPEDVKCGRLLCEIVKNTC-----KYDYSEDPNY

ADW54348.1 EYCTGQSVDCPIDHFHRNGKPCLNNNGYCYNGTCPMMQNQCHALFGPNAA----VAQDACFEENKKGKSYFYCRKEN---DVKIPCAPEDIKCGRLFCEIVKNTC-----KYDYSEDPDY

ADI47593.1 GMVD---------------------------------

ADI47643.1 GMVEPGTKCEDGKVCNSKRQCVDVTTV----------

ADI47598.1 G------------------------------------

ADI47635.1 GMVEPGTKCEDGKVCIN-RKCVDVNTAYLSTTGFSQF

ADI47601.1 GMVDPGTKCGDGMVCNRNRECVNVNTAY---------

ADW54341.1 GMVDEGTKCGEGKVCS-NGYCVDLNVAY---------

ADW54346.1 GMVDPGTKCEDGKVCS-NGKCVDVTTAYKSTSGFSQI

ADI47584.1 GMVDIGTKCADGKVCNSNRHCVDVNTAY---------

ADI47609.1 GMVDEGTKCGDGKVCS-NRHCVDVTTAY---------

ADI47604.1 GMVNPGTKCGDKKVCX-HRKCIDVNTTY---------

ANT80539.1 GMVDPGTKCEDGKVCS-NGHCVDVTTAY---------

ADI47664.1 GMVDPGTKCEDKKVCI-NGKCIDVNTAY---------

ADI47580.1 GMVEPGTKCEDKKVCI-NGKCTDVNTAY---------

ADI47592.1 GMVEPGTKCGDKKVCI-NGKCTDVNTAY---------

ADI47645.1 GMVDEGTKCGDGKVCS-NRHCVDVTRAY---------

CAJ01683.1 GMVEEGTKCGDGKVCS-KRHCVDVTTAY---------

ADI47583.1 GMVDEGTKCGDGKVCS-NRHCVDVNTAY---------

ADI47590.1 GMVDEGTKCADGKVCR-NRHCVDVTTAY---------

ADW54348.1 GMVGQGTKCEDGKVCS-NRHCVDVTTAY---------

**PLA_2_ (2 proteins)**

1OZ6 NLYQFGRMIWNRTGKLPILSYGSYGCYCGWGGQGPPKDATDRCCLVHDCCYTRV--GDCSPKMTLYSYRFENGDIICDNKDPCKRAVCECDREAAICLGENVNTYDKKYKSYED--CTEE

JAC96563.1 ---------SKVSG--SLVAQGAVGE--DLGRTIVALFFASRCCRVHDCCYQALSRRHCKPKMEKYFYSVRKDTVTCGGETECQQQTCECDKAAALCFRHS--KFQRQYIGYRNRLCEGP

1OZ6 VQEC-----------

JAC96563.1 TPPCQGVCRAPTKAG

**SVSP (7 proteins)**

ADI47565.1 MVLIRMLANLLVLQLSYAQKSSELVTGGDECNINEHPFLVALHTARSKRFHCTGTLINEQWVLTAARCNRKNIRIKLGVHNKNVRNENEEMRVPAEKVFCVSSKTYTRWDKDIMLIKMKR

ADI47570.1 MVLIRVLANLLVLQLSYAQKSSELVIGGAECNINEHRSLALIYNSTSMWFHCSGTLLNQEWVLTAAHCEMENMQIYLGVHNKTKRNKDQQKRFPKKKYFCLKSKNFTLWDKDIMLIKLNR

ADI47576.1 MVLIRVLANLLVLHFSYAQKSSELVIGGAECNINGHRSLAVLHNSSG--LLCSGTLINEEWVLSAAHCDMENMQIYLGYHNFSVPNDDEEIRVPVEKYFCLPRRNYIVWGKDIMLIKLNR

ADC52861.1 MVLIRVLANLLVLQLSYAQKSSELVIGGAECDKNEHRSLALVFNSSG--FICGGTLIHEEWVLSAAHCDVENMQIYLGLHNFSPPNKEHKKRVAKEKLFCLSSKSYTLWDKDIMLIKLDR

JAC96575.1 MVLIRVLANLLVLQLSYAQKSSELVIGGAECDINEHRSLVLVYNASG--FFCGGTLINREWVLSAAHCYMKNMRIYLGLHNFSLPNNDQQRRGARETYFCLPSRNYTKWDKDIMLIKL--

JAC96574.1 MVLIRVLANLLVLQLSYAQKSSELIIGGAECNINDHRFLVALYNYRTRAFLCGGTLINQEWVLSAAHCNRKSLQIKIGMHSKTAPNDDLQTRVPKETYFCLPNKNYTMWDKDIMLIKLNR

ADI47567.1 MVLIRVLANLLVLQLSYAQKSSELIIGGAECDINEHRSLALVYH---GNYQCGGTLINQEWVLTAAHCDGKTMKIHFGVHSKKKQNKDKQIRVPKEKFFCLPSRNYTMWDKDIMLIKLNK

ADI47565.1 PVNNSTHIAPLSLPSNPASVGSVCRIMGWGTITTTKVTYPDVPHCANIKIFDYSVCRGAYRKLPEKSRTLCAGVLEGGIDSCKADTGGPLICNGQFQGIASWGGQPCAQPLKPALYT---

ADI47570.1 PVKSSTHIEPFSLPSSPPSVGSVCRIMGWGAINSPNETFPDVPHCANIKLYNYSVCREAYGGLPEKSRTLCAGVLEGGIDTCMADSGGPLICNGQFQGIVSWGRHPCAQPLLPAFYT---

ADI47576.1 SVNISTHIAPLSLPSSPPSLDSVCRIMGWGAITSPNETYSNVPHCANINILHSSVCRAAYGRLPGLSRTLCAGILRGGIDSCKGDSGGPLICNGQFQGIVSWGDHPCGQRRKPGIYTKVF

ADC52861.1 PVKNSAHIAPLSLPSSPPRLHSVCRIMGWGSITSPNEKMSYVPHCANINILRYSMCQAIYGGLPDKSRTLCAGILGRRIGSCKGDSGGPLICDGQIQGIVSWVSKPCAHHGAPGMYTKVF

JAC96575.1 ------------------------------------------------------------------------------------------------------------------------

JAC96574.1 PVNNSTHIAPISLPSNRPSLGSVCRIMGWGSIIATNETYPDVPHCTNINLLHYSVCRAAYPWLPVNSRTFCAGVLQGGKDTCKGDSGGPLICNGQFQGILSWGWTPCAQLLEPALYTKVF

ADI47567.1 PVKNSTHIAPLSLPSNPPRLGSVCRIMGWGTISTTKVILPDVPHCVNINLLNYSECQAVHPELPEKGRTLCAGVLEGGKDTCHGDSGGPLICNGQIQGISSWGGDPCAQPHEPAL-----

ADI47565.1 --------------------

ADI47570.1 --------------------

ADI47576.1 DYTDWVQSIIAGNTAVTCPP

ADC52861.1 DYTDWIQSIMAGNTTATCPP

JAC96575.1 --------------------

JAC96574.1 DYSDWIQSIIAGNTTASCPP

ADI47567.1 --------------------

**LAOO (1 protein)**

CAQ72894.1 MNIFFMFSLLFLATLGSCADDKNPLEECFREADYEEFLEIAKNGLKKTSNPKDIVVVGAGMSGLSAAYVLAGAGHKVTVLEASQLVGGRVRTHRNAKEGWYANLGPMRIPEKHRIVREYI

CAQ72894.1 RKFGLELNEFVQETDNGWYFVKNIRKRVGEVKKDPGLLKYPVKPSEAGKSAGQLYQEALGKAVEELKRTNCSYMLNKYDTYSTKEYLIKEGNLSTGAVDMIGDLMNEDSGYYVSFVESMK

CAQ72894.1 HDDIFAYEKRFDEIVGGMDQLPTSMYRAIEKSVLFKARVTKIQQNAEKVRVTYQTAAKTLSDVTADYVIVCTTSRAARRINFKPPLPPKKAHALRSVHYRSATKIFLTCTKKFWEDDGIQ

CAQ72894.1 GGKSTTDLPSRFIYYPNHNFTSGVGVIIAYGIGDDSNFFLSLTLNECADIVFSDLSSIHQLPKNDIQKFCNPSVIQKWSLDRYAMGAITTFTPYQFQDYSKALTAPAGRVYFAGEYTANA

**ASPro (1 protein)**

CAJ55260.1 MLRSWEFVLLISCFLCFSSDALQRISLKKMPSIRETLQEMGMKVADVLPSLKHRISYLDEGLHNKTASTILTNFRDTQYYGEISIGTPAQIFKVVFDTGSSNLWVPSRQCSPLYSACVSH

NRYDSSESSTYKPKGTKITLTYAQGYIKGFFSQDIVRVADIPIIQFFTEAIALPSIPFIFARFDGVLGMGYPKQAIGGVIPVFDNIMSEKVLSENVFSVYYSRHSESNTGGEIILGGSDP

SHYTGDFHYVSTSREGYWHVDLKGVSIENKIVLCHDGCTATIDTGTSFISGPASSISVLMETIGATLSDGDYVIDCKKINLLPDITFHLGDMTYSLSSSTYVLKFSDETE

CTVAFMAVDIPPPLGPLWLLGATFIKQYYIEFDRQNNRIGFATSF

**PLB (1 protein)**

JAC96587.1 MIRFGNRSSSDKRRQRCWSWYWVGLLLLWAVAETRADIHYATVYWLEAEKSFQIQDVLDRNGDAYGYYNDTIQSTGWGILEIKAGYGNQHISNEILMYAAGFLEGYLTASHMSDHFANLF

PLMIKNVIIEQKVKDFIQKQDEWTRQQIKNNMDDPFWRNAGYVIAQLDGLYMGNVEWAKRQKRTPLNDFEINFLNALGDLLDLTTAFDSQLRKSDFLSMPDVSRIYQWDMGHCSALIKVL

PGYENIYFAHSSWFTYAATLRIYKHWDFKITDPQTKTGRASFSSYPGLLVSLDDFYILGSGLIMLQTTNSVFNLFLLKQVVPESLFAWERVRIANMMADSGKTWAQIFEKENSGTYNNQY

MILDTKKIKLQRSLEDGTLYIIEQIPKLVKYSDQTEVLRHGYWPSYNIPFHKVIYNMSGYTEYVQKLGLEFSYEMAPRAKIFRRDQGKVTDMESMKHIMRYNNYKEDPYTKHNPCNTICC

RQDLSRKTPVPAGCYDSKISDISMAAKFTTYAINGPPVEKDLPVFSWVHFNQTKHQSLPESYNFDFVTMKPVL

**Snaclec (6 proteins)**

AAP41218.2 -------------------------MCPPGWSSNGVYCYMLFKEPKTWDEAEKFCNKQGKDGHLLSIESKKEEILVDIVVSENIGK---MYKIWTGLSERSKEQHCSSRWSDGSFFRSYE

JAC96620.1 MGRFISVSFGLLVVFLSLSETGA--DCLPGWSSHEGHCYKVFNEYKSWKDAEKFCKKQGKSGHLVSIESSEEGDFVAQLISENLEKSQGIDFVWTGLTYKGRRKQCSSEWSDGSTVTYEN

Q7T2Q1.1 MGRFISVSFGLLVVFLSLSGTGADFDCPSDWTAYDQHCYLAIGEPQNWYEAERFCTEQAKDGHLVSIQSREEGNFVAQLVSGFMHRS--EIYVWIGLRDRREEQQCNPEWNDGSKIIYVN

JAC96617.1 MGRFIPVSFSLLVLFLSLSGTGADFECPPEWSSYDLHCYKAFDKPKRSRDAEKFCTEQAKGGHLASIESSEEGDFVAKLISENIKSS--ADYVWIGLWNKRREQYCTSQWTDGSNVIYKN

Q6X5S4.1 MGRFIFISFGLLVVFLSLSGTEA--ECLPDWFHYEGHCYRVFDEPKTWADAEKFCSEQANGGHLVSVHSKKEAGLVGVLAYQTLE----SPIVWMGLSKIW--NQCDWTWTNGAKLKYEA

P81996.1 -------------------------NCLPDWSVYEGYCYKVFKERMNWADAEKFCMKQVKDGHLVSFRNSKEVDFMISLAFPMLK----MELVWIGLSDYW--RDCYWEWSDGAQLDYKA

AAP41218.2 IAI-RYSECFVLEKQSVFRTWVATPCENTFPFMCKYPVPR

JAC96620.1 WNKEEPRKCVGLE---------------------------

Q7T2Q1.1 WKEGESKMCQGLTKWTNFHDWNNINCEDLYPFVCKFSAV-

JAC96617.1 VIERFTKNCFGLEKKTEYRTWFNLRCGDDYPFVCKFPPQC

Q6X5S4.1 WAEES--YCIHI--TSKKKEWKSLPCRNYGHFVCKSPA--

P81996.1 WDNER--HCFAA--KTTDNQWMRRKCSGEFYFVCKCPA--

**CRISP (1 protein)**

P0DMT4.1 NVDFDSESPRKPEIQNEIIDLHNSLRRSVNPTASNMLRMEWYPEAAANAERWAFRCTLNHSPRDSRVIDGIKCGENIYMSPYPIKWTAIIHKWHDEKKNFVYGIGASPANAVIGHYTQIV

VWYKSYRGGCAAAYCPSSAYKYFYVCQYCPAGNIIGKTATPYKSGPPCGDCPSACDNGLCTNPCTREDEFINCNDLVKQGCQTDYLKSNCAASCFCHSEIK

**Disintegrin (3 proteins)**

ANJ00861.1 MIQVLLVTICLAVFPYQVSSKTLKSGSVNEYEVVNPGTVTGLPKGAVKQPEKKHEPMKGNTLQKLPLCTTGPCCRQCKLKPAGTTCWRTSVS--SHYCTGRSCECPSYPGNG----

P81631.1 -----------------------------------------------NSVHPCCDPVKC-EPREGEHCISGPCCRNCKFLNAGTICKRAMLDGLNDYCTGISTDCPRNRYKGKED-

P0C6A3.1 -----------------------------------------------NSAHPCCDPVKC-EPREGEHCISGPCCRNCKFLNAGTICKKAMLDGLNDYCTGISSDCPRNRYKGKEDD

**NGF (1 protein)**

JAC88974.1 MSMLCYTLIIAFLIGIWAAPKSEDNVSLGSPATPDISDTSCAKTHEALKTSQNTDQHSPAPKKAEDQEFGSAANIIVDPKLFQKRRFQSPRVLFSTQPPPLSRDEQSVEFLDNADSLNRN

IRAKRGTHPVHNQGEFSVCDSVSVWVANKTTATDIRGNEVTVMVDVNLNNNVYKQYFFETKCKNSNPVPSGCRGIDAKHWNSYCTTTDTFVRALTMEGNQASWRFIRINTACVCVISRKN

DNFG

**Supplementary Fig S2c.** Alignment of tryptic and semi-tryptic peptide sequences with *Echis carinatus* proteins from NCBI database. The protein alignment was done using Clustal Omega programme (<https://www.ebi.ac.uk/Tools/msa/clustalo/>). The number of proteins in each protein classes is shown in parenthesis. The distinct peptides obtained for each of the following proteins has been highlighted in yellow or green (two colours have been used in case of adjacent distinct/unique peptides). The amino acid substitutions within the distinct peptides obtained from MS/MS are highlighted in red colour. The LC-MS/MS identified peptides other than distinct peptides are shown in blue colour.

**SVMP (12)**

**CLASS P-II (1 protein)**

ADI47725.1 DLNIYITLAALEIWSKRDLINVVSSSIDTLRSFAEWRERDLLNRRPHDNAQFLTNINFDDNVIGRAYERSMCDPKTSVGIVKDHSTVHLYVASTMAHEMGHNLGMDHDGSQCNCGGAGCV

MSATITRSRSYKFSDCSKNQYQNYLTNYKPQCIINQPLRTDTVSTPVSGNELLQNSADPCYDPLTCHPREGEECESGPCCRNCKFLKEGTICKRARGDDMDDYCNGKTCDCPRNPHKGPA

TAKGSVLM

**CLASS P-III (11 proteins)**

ADI47581.1 MIQVLLVIICLAVFPYQGCSIVLGSGNVNDYEVVYPQKVTALPKGAVQQPEQKYEDAMQYEFEVNGEPVVLHLEKNKGLFSEDYSETHYSPDGREITTKPSVEDHCYYHGRIQNDADSSA

ADI47593.1 ------------------------------------------------------------------------------------------------------------------------

ADI47578.1 -----------------------------------------------KQPEQKYEDTMQYEFKVKGEAVVLHLEKNKGLFSEDYTETHYAPDGREITTKPAVEDHCYYHGRIQNDADSSA

ADI47590.1 MMQVLLATICLAVFPYQGSSIILESGNVNDYEVVYPQKVTALPQGAIQQPEQKYEDTMQYEIEVNGEPVVLHLEKNKDLFSEDYSETHYSPDGRKITTNPPVQDHCYYHGRIQNDAHSTA

ADI47585.1 MMQVLLVTICLAVFPYQGSSIILESGNVNDYEVVYPQKVTALPEGAIQQPEQKYEDAMQYEFEVNGEPVVLRLEKNKDLFSEDYSETHYSPDGREITTNPPVEDHCYYHGRIQTDAHSSA

ADI47582.1 ----------------------------------------------------------------KGEPVVLHLEKNKDLFSEDYSETHYSPDGREITTNPLVKDHCYYHGRIQNDADSTA

ADI47604.1 ------------------------------------------------------------------------------------------------------------------------

ADI47580.1 MMQVLLATICLAVFPYQGSSITLESGKINDYEVVYPEKVTALPKGAIQQTEQKYEDAMQYEVKVKGEPVVLHLKKNKDLFSEDYSETHYTPDGREITTNPPIEDHCYYHGSIQNDAHSSA

ADI47589.1 MMQVLLATICLAVFPYQGSSIILESGNINDYEVVYPEKVTALPKGAIQQTEQKYEDAMQYEFKVKGEPVVLHLKKNKDLFSEDYSETHYTPDGREITTNPPIEDHCYYHGSIQNDAHSSA

ADI47597.1 ------------------------------------------------------------------------------------------------------------------------

ADI47588.1 ------------------------------------------------------------------------------------------------------KDHCYYHGRIQNDDHSTA

ADI47581.1 SISACNGLKGHFKLRGEMYLIEPLKIPDSEAHAVYKYENIEKEDEAPKMCGVTQENWESHEPIKKTLGLIFPPHKGKFDK--KFIELAHNCGPQVVCTKYNDSTAMRTWIYEMVNTVNEI

ADI47593.1 ------------------------------------------------------------------------------------------------------------------------

ADI47578.1 SISACNGLKGHFKLRGEMYFIEPLKIPDSEAHAVYKYENIEKEDEAPKICGVKKTNWESDKSIQEASQLNLTPEQQRYLNSEKHIKVAIIADYLIYRKYGRNLFTIRTRIYEIINILNAI

ADI47590.1 SMSACNGLKGYFKLRGETYFIEPLKIPDSEAHAVYKYENLEKEDETPKICGVTQTNWESDEPIKKASQLVATSEQQRYYDRFRYVXXIVVVDHRMCKKYNGNLRRIRRRLYQLINFLNEM

ADI47585.1 SMSACNGLKGHFKLRGETYFIEPLKIPDSEAHAVYKYENVEXENEAPKMCGVTQTNWESDEPIKEASQLVATSEQQRYYDRFRYVKFIVIADHRMVEKYNHNLQKIRTKIYQIVNFLNEM

ADI47582.1 SISACNGLKGHFKLRGETYFIEPLKIPNSEAHAVYKYENIEKEDEAPKMCGVTQDNWESDEPIEKASQLVATSEQQTYL-----------------------------------------

ADI47604.1 ------------------------------------------------------------------------------------------------------------------------

ADI47580.1 SISACNGLKGHFRLRGETYFIEPLKIPDSEAHAVYKYENIEKEDEAPKMCGVTHTNWESDEPIEKASQLVATSAQHSYYDHFRYVKVIVVVDHSMCTKYNNDLTAMRTRVYEMINTVNEI

ADI47589.1 SISVCNGLKGHFRLRGEMYFIEPLKIPDSEAHAVYKYENIEKEDEAPKMCGVTHTNWESDETIEKASQLVATSEQHSYYDYFRYVKVIVVVDHSMCTKYNNDLTAMRTRVYEMFNTVNEI

ADI47597.1 -------------------------------------------------------------------------------------------------------------------TLNLV

ADI47588.1 SISTCNGLKGHFRLRGETYLIEPLKLPNSEAHAVYKYENIEKEDEAPKMCGVTQTNWKSDEPIK-APQFILTPEQRAYLNANKYIELSIVVDNVMYRKYTGDVTAIKTRIYEMVNTLNLI

ADI47581.1 YLPLNIRVPLCGLEFWCNGDLINVTFTADDTMDSFGEWRVSDLLNRKKHDYVQLLTNITLDFKSLGMAFVDGMCKPYRSVGLIKDNS-TTFKTAVIMAHEMGHSLGMHHDSRSCNCAAYP

ADI47593.1 ----------------------------------------------------------------------------------------------------MGHSLGMLHDTKSCTCGANP

ADI47578.1 YRAFHMHVALVFLEIWSNGDKINVLPAANVTLDLFGKWRLSDLLNRREHDNAQLLTGINFDGPTAGLGYVGSMCEPQYSAAIVQDHNKINILVAMAMAHELGHNLGMNHDEKFCTCGAKS

ADI47590.1 YLPWNLRVPLVGMEFWNQRDLINVTSSPKVTLDLFGEWRESDLLNRKEHDYAQLLTAIDFDGPTIGMAHIATMCHSKLSVGIVEDYRPAESVVAAIMAHEMGHNLGLIHDENQCNCDADS

ADI47585.1 YLPWNIRVPLVGLEIWNQRDLINVTSSPKVTLDLFGEWRESDLLSRKRHDNAQLLTAIDFDEATIGMAHIATMCHSKLSVGVVEDHSPIHLSVAVTMAHEMGHNLGINHDTSFCTCHANS

ADI47582.1 ------------------------------------------------------------------------------------------------------------------------

ADI47604.1 ------------------------------------------------------------------------------------------------------------------------

ADI47580.1 YIYMHIRVPLVGIEFWCNGDLINVTSSAEYTLNSFGNWRASGLLSHKTHDNAQLLTAIDISGSVIGKAHISSMCQATRSVGVVEDHSPIVREVAVTMAHEMGHNLGMYHDGNQCTCGASS

ADI47589.1 YIYMHIHVPLVGIEFWCNGDLITVTSSAENTLYSFGDWRASYLLSHKPHDNAQLLTAIDISNSTIGIAHVSSMCQATRSVGVIEDHSPIVRAVAVTMAHEMGHNLGMYHDGKQCNCGADS

ADI47597.1 YIVFSIHIALTHIEIWSETDQIKVQSAADDTLHLFGDWREKNLMKRKEHDNAQLVTGINFNGQTLGRAPVSGMCSPRRSVGVIQDYCKTHLLTAVVMAHELGHNLGMNHDKSHCKCPVKS

ADI47588.1 YTVLNIHIALIYLEIWSEGDLINVQSVVDITLDSFGEWRKRYLLNRKDHDNAQLLTGINLNGNTIGYGYVGSMCMPKESVGIVQDHSKTYLSVAITMAHELGHNLGINHDKDSCTCQASS

ADI47581.1 CIMSPVLGKKPSNKFSSCSYDYYKEYLLKYKPKCILDPPLRKDIASPAVCGNEIWEEGEECDCGSPEDCQNPCCEAETCELYPGAVCEDGLCCDKCKFRTAGTECRKASDECDVPEYCTG

ADI47593.1 CIMFSEVSEPTPKEFSRCSYDQYRDYLPKYNPKCIFDPPLRNDIVSPAVCGNEIWEEGEECDCGSPADCENSCCDAATCKLKPGAECGNGECCDKCKIRTAGTECRAARDDCDVPEHCTG

ADI47578.1 CIMSGTLSCEGSFRFSNCSQEENRKYLIRKMPQCILKKPLKTDIVSPPVCGNYLVELGEDCDCGTPTFCQNPCCNAATCKLTPGSQCADGECCDQCRFRRAGTECRPAKDECDMADLCNG

ADI47590.1 CIMYHQINNPPPMYFSNCSWNYYQNFLTNYKPDCTLIRPSRTDIVSPPVCGNALLEKGEECDCGSPANCQDPCCDAASCKLHSWVECEIGECCDQCRFKPAGTECRAIRSECDLPEYCTG

ADI47585.1 CIMETYLSDPPPVYFSNCSWDQYQKFLTNFKPDCTLIRPTKTDIVSPPVCGNELLEKGEECDCGSPENCQDPCCDAASCKLHSWVECEIGECCDQCRFKAAGTECRGLRSECDLPEYCTG

ADI47582.1 -------------------------------------EPLGTDIVSPAVCGNELLEVGEECDCGSPANCRDPHCDAALCKLHLGVECESGECCQQGRFMSAQTECRPTRSECDLPEYCTG

ADI47604.1 CIMYPYLSEPLPMFFSNCSKDQYQKFLTNFKPDCTLIRPSRTDIVSPPVCGNDLLERGEECDCGSPENCENPCCDAASCKLHSWIECEFGECCEQCRLKPAGTECRGIRNECDLPEYCTG

ADI47580.1 CIMSRFLTDPPPMYFSNCSWNYYQNFLTNDKPDCTRIRPSRTDIVSPQVCGNGLLEKGEECDCGSPANCRYPCCDAASCKLHSWVECESGECCDQCRFKPAGTECRAIRSECDLPESCTG

ADI47589.1 CIMYPYLSDPLPMYFSNCSWNYYQNFLTNYKPDCTRIKPSRTDIVLPPVCGNGLLEKGEECDCGSPANCQYPCCDAASCKLHSWVECESGECCDQCRFKAAGTECRAIRNECDLPESCTG

ADI47597.1 CIMSPTARPEPVFSFSDCSWNDYRSFRDSDQSKCIDNKPLKTDIVSPSVCGNNFVEVGEECDCGSPKYCRNPCCDAATCKLKPGTECGDGMCCDQCRFKPAGTQCRGTRSDCDVPEYCTG

ADI47588.1 CIMAATISDQPSYQFSDCSKNELWGYFISHTPRCILNEPLRTDVVSPAVCGNYVVEEGEECDCGSLWYCRNPCCDATTCKLKPGAECGEGMCCHQCRFATAETVCRPAKSECDMAEYCTG

ADI47581.1 QSADCPRNEFQRNGQPCLNNLGYCYNGDCPIMKNQCISLFGS----RATVAEDSCFQENLKGSTHGYCGKEN---GRKIPCAPQDVKCGRLYCLDNSSRKKNPCKMHYLDADQHKGMVEP

ADI47593.1 QSAECPRNEFQRNGQPCLNNSGYCYNGDCPIMKNQCILLFSP----NATVDVDACFQWNLRGIFDGYCTKEIGSYGRRFPCAPQDVKCGRLYCLDKSARKKKRCKTNYSPDDENKGMVD-

ADI47578.1 QSDECPKDQFQRNGHPCQNNNGYCYNGKCPVMGNQCISLFGS----RATVAEDACFQFNRLGSDYGYCRKEN---GIKIPCAPEDVKCGRLYCFDNLPEHKNPCQIYYTLRDENKGMVEP

ADI47590.1 QSVDCPIDRFHRNGKPCQSNNGYCYNGACPIMQNQCYALFGP----DATVGQDACFEENKKGKSYFYCRKEN---DVKIPCAPEDVKCGRLLCEIV----KNTCKYD-YSEDPNYGMVDE

ADI47585.1 QSVDCPIDHFHRNGKPCQNNNGYCYNGTCPIMQNQCYALFGP----DAVVGQDGCFEMNKKGEGDFYCRKEN---DVKIPCAPEDIKCGRLFCEIV----KNTCKYD-YSEDPNYGMVDE

ADI47582.1 QSAECPTDHFHRNGQPCLNNQGYCYNGKCPHMYHQCYSLFGS----IATVGQDGCFEWNKKAESYFYCRKEN---DVMIPCAPEDVKCGRLFCEII----KDTCKYD-YSEDPNYGMVDE

ADI47604.1 QSGDCPTDHFHRNGKPCLYNNGYCYNGACPIMEYQCYDHFGP----DAVVSQDACFEKNKEGKGDFYCRNEN---YVKTPCAPEDVKCGRLFCDIG----PNRCIFP-YGD---TGMVNP

ADI47580.1 QSAECPTDHFHKNGKPCLNNYGYCYNGKCPIMEYQCYNHFGS----NATVGQDGCFEMNKRGERDFYCRKEN---DVPVPCAQEDVKCGRLFCETE----PNMCRHP-YGD---EGMVEP

ADI47589.1 QSGDCPIDQFHRNGQPCLHNYGYCYNGKCPIMEYQCYDHFGS----NAVVGQDACFKWNTKGKSDFYCRKEN---DVKIPCAPEDVKCGRLFCDIG----PNRCRYP-YGD---EGMVEP

ADI47597.1 RSAECPLDVFQRNGQPCQSNNGYCYNGNCPIMTNQCIDLWKPAPLAGVNVAPDRCFDYNLQGTDKYHCGIKN---GRYIKCARQDIKCGRLFCVEPSTGNKITCQSFRSQDDPDYGMVDI

ADI47588.1 RSADCPTDYFHRNGQPCLLNHGYCYNGTCPIMIHQCIILWGT----GATVSPDICFQENNKGQGYFYCRREN---NKNIPCALRDVKCGRLFCKLPIDNTP-LCNYRYSDVSLDYGMVDP

ADI47581.1 GTKCEDGKVC-INRKCVDINTAY

ADI47593.1 -----------------------

ADI47578.1 GTKCENGKVC-INGKCVDVNTAY

ADI47590.1 GTKCADGKVC-RNRHCVDVTTAY

ADI47585.1 GTKCEDGKVC-SNRHCVDVTTAY

ADI47582.1 GTKCGDGKVC-SNRHCVDVNTAY

ADI47604.1 GTKCGDKKVC-XHRKCIDVNTTY

ADI47580.1 GTKCEDKKVC-INGKCTDVNTAY

ADI47589.1 GTKCGDKKVC-INGKCTDVNTAY

ADI47597.1 GTKCADGKVCNSNRHCVDVNTAY

ADI47588.1 GTKCGDGMVCNRNRECVNVNTAY

**PLA_2_ (1 protein)**

1OZ6 NLYQFGRMIWNRTGKLPILSYGSYGCYCGWGGQGPPKDATDRCCLVHDCCYTRVGDCSPKMTLYSYRFENGDIICDNKDPCKRAVCECDREAAICLGENVNTYDKKYKSYEDCTE

EVQEC

**SVSP (1 protein)**

ADI47565.1 MVLIRMLANLLVLQLSYAQKSSELVTGGDECNINEHPFLVALHTARSKRFHCTGTLINEQWVLTAARCNRKNIRIKLGVHNKNVRNENEEMRVPAEKVFCVSSKTYTRWDKDIML

IKMKRPVNNSTHIAPLSLPSNPASVGSVCRIMGWGTITTTKVTYPDVPHCANIKIFDYSVCRGAYRKLPEKSRTLCAGVLEGGIDSCKADTGGPLICNGQFQGIASWGGQPCAQP

LKPALYT

**Snaclec (5 proteins)**

Q6X5S4.1 MGRFIFISFGLLVVFLSLSGTEAECLPDWFHYEGHCYRVFDEPKTWADAEKFCSEQANGGHLVSVHSKKEAGLVGVLAYQTLE----SPIVWMGLSK--IWNQCDWTWTNGAKLKYEAWA

P81996.1 -----------------------NCLPDWSVYEGYCYKVFKERMNWADAEKFCMKQVKDGHLVSFRNSKEVDFMISLAFPMLK----MELVWIGLSD--YWRDCYWEWSDGAQLDYKAWD

P81017.1 ---------------------DQDCLSGWSFYEGHCYQLFR-LKTWDEAEKYCNQ-WDGGHLVSIESNAKAEFVAQLISRKLPKSAIEDRVWIGLRDRSKREQCGHLWTDNSFVHYEHV-

AAP41218.2 -----------------------MCPPGWSSNGVYCYMLFKEPKTWDEAEKFCNKQGKDGHLLSIESKKEEILVDIVVSENIGK---MYKIWTGLSERSKEQHCSSRWSDGSFFRSYEIA

Q9PSM9.1 -----------------------DCLPGWSSHEGHCYKVFNEYKTWKDAEKFCKKQGKSGHLVSVESSEEGDFVAKLISENLEKSHSIDFVWTGLTYKGRWKQCSSEWSDGSKIKYQKWG

Q6X5S4.1 --EESYCIHIT--SKKKEWKSLPCRNYGHFVCKSPA--

P81996.1 --NERHCFAAK--TTDNQWMRRKCSGEFYFVCKCPA--

P81017.1 -VPPTKCFVLERQTEFRKWIAVNCEFKFPFVCKAKIPR

AAP41218.2 -IRYSECFVLEKQSVFRTWVATPCENTFPFMCKYPVPR

Q9PSM9.1 KQQPRKCLGLEKQTEFRKWVNLYCEEPQRFTCEI----

**CRISP (1 protein)**

P0DMT4.1 NVDFDSESPRKPEIQNEIIDLHNSLRRSVNPTASNMLRMEWYPEAAANAERWAFRCTLNHSPRDSRVIDGIKCGENIYMSPYPIKWTAIIHKWHDEKKNFVYGIGASPANAVIGH

YTQIVWYKSYRGGCAAAYCPSSAYKYFYVCQYCPAGNIIGKTATPYKSGPPCGDCPSACDNGLCTNPCTREDEFINCNDLVKQGCQTDYLKSNCAASCFCHSEIK

**Disintegrin (2 proteins)**

P82465.2 MIQVLLVIICLAVFPYQGSSIILESGNINDYEIVYPKKVAVLPTGAMNSVHPCCDPVTCEPREGEHCISGPCCRNCKFLNAGTICKKAMLDGLNDYCTGISSDCPRNRYKGKEDD

Q5EE07.1 -----------------------------------------------NSVHPCCDPVKCEPREGEHCISGPCCRNCKFLNAGTICKRAMLDGLHDYCTGVTSDCPRNRYNH----

**Supplementary Fig S2d.** Alignment of tryptic and semi-tryptic peptide sequences with Transcriptome Shotgun Assembly (TSA) sequences of *Echis coloratus* from NCBI database. The protein alignment was done using Clustal Omega programme (<https://www.ebi.ac.uk/Tools/msa/clustalo/>). The number of proteins in each protein classes is shown in parenthesis. The distinct peptides obtained for each of the following proteins has been highlighted in black or blue (two colours have been used in case of adjacent distinct/unique peptides) or green (for overlapping regions of distinct/unique peptides). The amino acid substitutions within the unique/distinct peptides obtained from MS/MS are highlighted in yellow colour. The LC-MS/MS identified peptides other than unique/distinct peptides are shown in blue colour.

**SVMP (6 proteins)**

JAC96602.1 ------------------------------------------------------------------------------------------------------------------------

JAC96600.1 ------------------------------------------------------------------------------------------------------------------------

JAC96593.1 ------------------------------------------------------------------------------------------------------------------------

JAC96608.1 ------------------------------------------------------------------------------------------------------------------------

JAC96604.1 FPYQGSSKTLKSGNVNDYEVVNPQKITGLPVGAFKQPEKKHEDAVQYEFEVNGEPVVLHLEKNKGLFSEDYSETHYSPDGREITTNPPVEDHCYYHGRIKNDADSTASISTCNGLKGFFT

JAC96597.1 ------------------------------------------------------------------------------------------------------------------------

JAC96602.1 --------------------------------------------ESDEPIKKTSQLAATSEQQRF-DPRHIQLIIVADHAMFVKYNSDSTAITTWVHQIVNDMIVMYRDLNIHITLAALE

JAC96600.1 ----------------------------------------QTNWESDEPFKASQLNLTPEQRTYLKSKKYIELVIVADYIMFWKYDHDLSTIRTRIYEIVNTLNVIYRVLNIYVALVGLE

JAC96593.1 ------------------------------------------------------------------------------------------------------------------------

JAC96608.1 ------------------------------------------------------------------------------------------------------------------------

JAC96604.1 LRGETYLIEPLKVPDSEAHAVYKYEDGKKKDEAPKMCGVTQTNWESDEPIKKASGLIVPSQKRKL-DKKFIELVIVVDHSVVTKYNNDSTAIRTWIYEMLNTVNEIYLPLNIRVTLVGLE

JAC96597.1 ------------------------------------------------------------------------------------------------------------------------

JAC96602.1 IWSNGDLITVTSSAPTTLRSFGEWRERDLLNRRTHDNAQLLTAVHLD-NLIGYGFVGTMCDPKWSVGITEDHSTIHLWVAATMAHEMGHNLGINHDGNQCNCGAAGCIMSAIISE-----

JAC96600.1 IWCKGNLINVTSSAYDTLDSFGEWREKDLLNRKRHDNAQLLTGIDFSGAAAGRGYVGRMCQPKYSVGIVQDHNKIYLLVASAMAHEMGHNLGMDHDGIHCTCGAKSCIMSGILRC-----

JAC96593.1 ------------------------------------------------------------------------------------------------------------------------

JAC96608.1 ------------------------------------------------------------------------------------------------------------------------

JAC96604.1 FWSNRDLINVTFTADDTMDSFGEWRVSDLLNRKRHDYAQLLTNITLDFDSLGMAFIDGMCKSYRSVGLIRDCSNTTFKTAVIMAHEMGHSLGMHHDSKSCKCVASPCIMSKALGK-----

JAC96597.1 ------------------------------------------------------------------------------------------------------------------------

JAC96602.1 HPSYQFSDCSMNDYQSYLTNHNPQCILNQPLRTDTVSTPVSGNELL-----------QNSANPCYDPATCQPTEGADCASGPCCHDCKFLKEGTICKRARGD-NMHDYCNGKTCDCPRNP

JAC96600.1 ETSYLFSDCSREAHRKYLINNMPQCILNKPLKTDIVSPPVCGNYFVEVGEECDCGSPRNCQDQCCDAATCKLRPGAQCGEGVCCYQCKFKRAGTVCRPANGECDVSDHCTGQSAECPTDH

JAC96593.1 -------------------------------------------------------------------------------------------------------------TGQSAECPLDV

JAC96608.1 --------------------------------------------------------------------------------------------GTECRPAKDDCDMAESCTGQSSVCPVDS

JAC96604.1 QPSKVFSSCSYDDYNTYLLKYKPKCILDPPLRKDIASPAVCGNEIWEEGEECDCGSPEDCQNPCCDAETCELYPAAVCEDGPCCHKCKFKTAGTECRPARDECDVAEHCTGQSSECPRNE

JAC96597.1 --------------------------------------------------------------------------------DSAFHPSEFRPAGTECRGTSSDCDVPEYCTGQSAECPADQ

JAC96602.1 HKGEHDPMEWPAPA--KGS-VLM-------------------------------------------------------------------------------------------------

JAC96600.1 FQKNGQPCLLNRGYCYNGRCPIMIHQCIILWGPGTTVSPDICFQENNKGQGYFYCRRENNKNIPCAPQDVKCGRLFCKLPI-HNTHPCNYRYSDVALDYGMVDPGTKCGDGMVCNGNREC

JAC96593.1 FQRNGQPCQSNNGYCYNGKCPIMTNQCIHLWKPGVNVAPDACFEYNLQGTYKHHCGSENGRYIKCARQDIKCGRLFCVEPSTGNTITCQIFRSQDDPDYGMVDIGTKCADGKVCNSNRHC

JAC96608.1 FHENGQPCLHNLGYCYNGKCPITLYQCRAFLGNNAVGVDESCFQYNRLGNSYAYCRKENGIKIPCAPKDEKCGRLYCSYNSFGNHISCLPCYRADEEDKGMVDEGTKCGDGKVCS-NRHC

JAC96604.1 LQRNGQPCLKNSGYCYNGDCPIMTNQCISLFGSRATVAEDSCFQENLKGSKHGYCAKENGRKIPCAPQDVKCGRLYCLDNSSRKKNPCKMHYLDADQHKGMVEPGTKCEDGKVCI-NRKC

JAC96597.1 FQRNGQPCQNNNGYCYNGICPIMRNQCILLFGSRATVAEDACFQFNSLGSDYGYCRKENGRKIPCAPEDVKCGRLYCFDNLPEHKNPCQIVYTPSDEDKGMVDPGTKCEDGKVCI-NGKC

JAC96602.1 ----------------

JAC96600.1 V---------------

JAC96593.1 VDVNTAY---------

JAC96608.1 VDVTTAY---------

JAC96604.1 VDVNTAYLSTTGFSQF

JAC96597.1 VDVNTAY---------

**PLA_2_ (1 protein)**

JAC96563.1 -----------SKVSGSLVA----------------QGAVG--EDLGRTIVALFFASRCCRVHDCCYQALSRRHCKPKMEKYFYSVRKDTVTCGGETECQQQTCECDKAAALCFRHSKF-

- QRQYIGYRNRLCEGPTPPCQGVCRAPTKAG

**LAAO (1 protein)**

JAC96580.1 MNVFFMFSLLFLATLGSCADDKNPLEECFREADYEEFLEIARNGLKKTSNPKDIVVVGAGMSGLSAAYVLAGAGHKVTVLEASERVGGRVRTHRNTKEGWYANLGPMRIPEKHRIIREYIRKFGLELN

EFVQETDNGWYFIKNIRKRVGEVKKDPGLLKYPVKPSEAGKSAGQLYQASLKKAVKELKRTNCSYMLNKYDTYSTKEYLIKEANLSPGAVDMIGDLLNEDSGYYVSFIESLKHDDIFAYEKRFDEIVG

GMDRLPTSMYRAIEKSVLFKARVTKIQQNAEKVRVTYQTAAKTLSYVTADYVIVCTTSRAARRINFKPPLPPKKAHALRSVHYRSGTKIFLTCTKKFWEDDGIHGGKSTTDLPSRFIYYPNHNFTSGV

GVIIAYGIGDDANFFQALSLNECADIVFNDLSSIHQLPKSDIQKFCCPSMIQKWSLDKYAMGAITTFTPYQFQHFSEALTAPAGRIYFAGEYTANAHGWIDSTIKSGLTAARDVNRASEL

**SVSP (2 proteins)**

JAC96577.1 --------------------------GGAECNINEHRSLALIYNSTSMWFHCSGTLLNQEWVLTAAHCEMENMQIYLGVHNKTKRNKDQQKRFPKKKYFCLKSKNFTLWDKDIMLI----

JAC96575.1 MVLIRVLANLLVLQLSYAQKSSELVIGGAECDINEHRSLVLVYN--ASGFFCGGTLINREWVLSAAHCYMKNMRIYLGLHNFSLPNNDQQRRGARETYFCLPSRNYTKWDKDIMLIKL--

JAC96577.1 ------------------------------------------------------------------------------------------------------------------------

JAC96575.1 ------------------------------------------------------------------------------------------------------------------------

JAC96577.1 --------------------

JAC96575.1 --------------------

**Snaclec (1 protein)**

JAC96617.1 MGRFIPVSFSLLVLFLSLSGTGADFECPPEWSSYDLHCYKAFDKPKRSRDAEKFCTEQAKGGHLASIESSEEGDFVAKLISENIKSS--ADYVWIGLWNKRREQYCTSQWTDGSNVIYKN

VIERFTKNCFGLEKKTEYRTWFNLRCGDDYPFVCKFPPQC

**CRISP (1 protein)**

JAC96631.1 MIAFIVFPILAAVLQQSSGNVDFDSESPRKPEIQNEIIDLHNSLRRSVNPTASNMLRMEWYPEAAANAERWAFRCTLNHSPRDSRVIDGIKCGENIYMSPYPIKWTAIIHKWHDEKKNFVYGIGA

SPNAVIGHYTQIVWYKSYRGGCAAAYCPSSAYKYFYVCQYCPAGNIIGKTATPYKSGPPCGDCPSACDNGLCTNPCTREDEFINCNDLVKQGCQTDYLKSNCAASCFCHSEIK

**VEGF (1 protein)**

JAC96562.1 MAAYLLAVAILFCIQGWPSVRVQGQVRPFLEVYQRSTCQAREMLVSLLEEYPDEISDIFRPSCVAVLRCGGCCSDESFTCTPVEERTVDLQIMRVNPHTQSSKMEAMKFTEHTACECRPQLGSGV

NIGKRNRSPEEGEREPSSL

**NGF (1 protein)**

JAC88974.1 MSMLCYTLIIAFLIGIWAAPKSEDNVSLGSPATPDISDTSCAKTHEALKTSQNTDQHSPAPKKAEDQEFGSAANIIVDPKLFQKRRFQSPRVLFSTQPPPLSRDEQSVEFLDNADSLNRNIRAKRG

THPVHNQGEFSVCDSVSVWVANKTTATDIRGNEVTVMVDVNLNNNVYKQYFFETKCKNSNPVPSGCRGIDAKHWNSYCTTTDTFVRALTMEGNQASWRFIRINTACVCVISRKNDNFG

**Supplementary figure S3:** Representative annotated MS/MS spectra of the ions with mass **(a)** 1250.5966 (^2+^), **(b)** 1118.5608 (^3+^), **(c)** 733.3646 (^2+^), **(d)** 798.3912 (^2+^), **(e)** 929.4719 (^2+^), **(f)** 1741.8192 (^2+^), **(g)** 2942.252 (^2+^), **(h)** 907.4837 (^2+^), and **(i)** 1103.5645 (^2+^). The MS/MS spectra were annotated by PEAKS 8.5 software.

**a) ADI47597.1, metalloproteinase, partial**


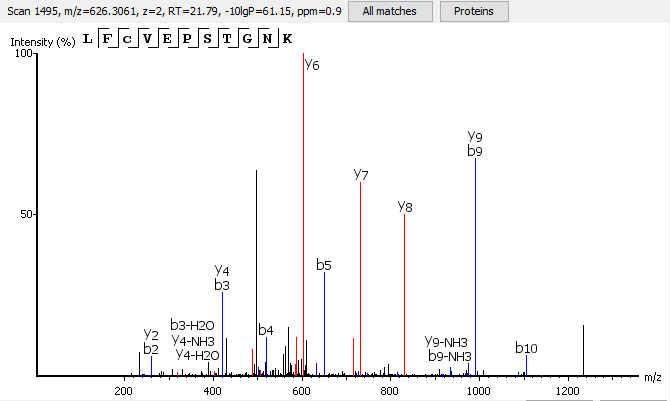


**
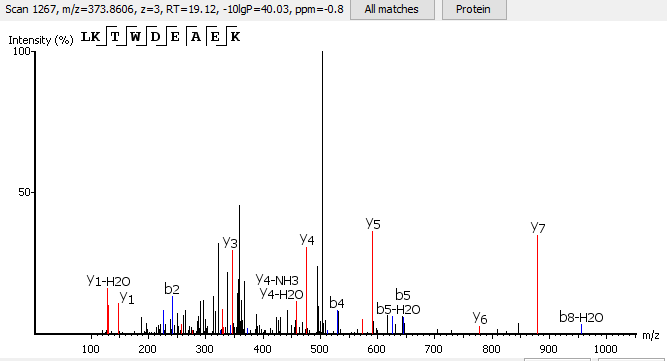
**

**b) P81017.1, Snaclec echicetin subunit alpha**

**c) Q6X5S4.1, Snaclec 9**

**
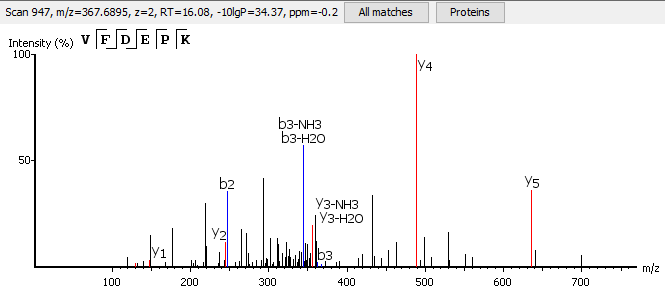
**

**d) Q9PSM9.1, ECLV IX/X-BP subunit A**

**
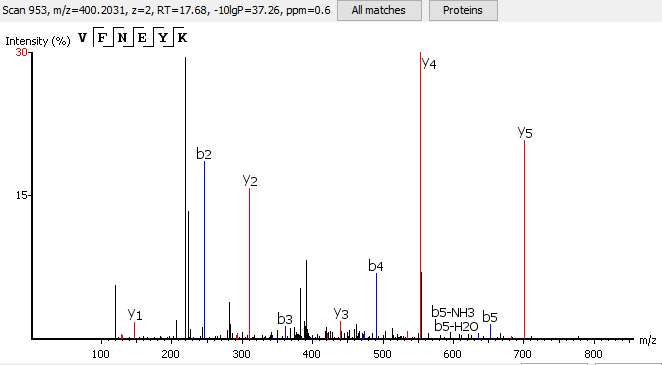
**

**e) AAP41219.2, echicetin B-chain**

**
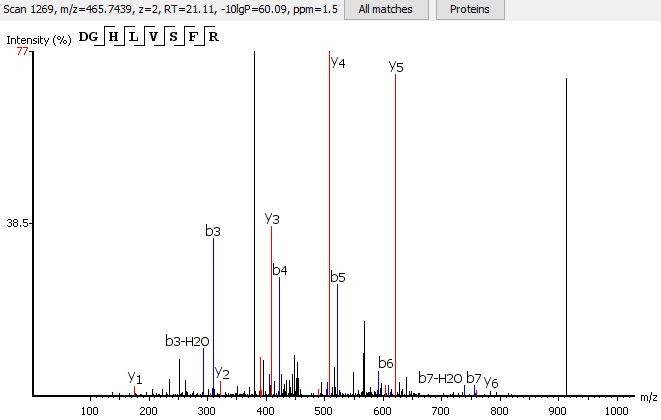
**

**f) ANT80539.1, venom metalloprotease PIII-SVMP**

**
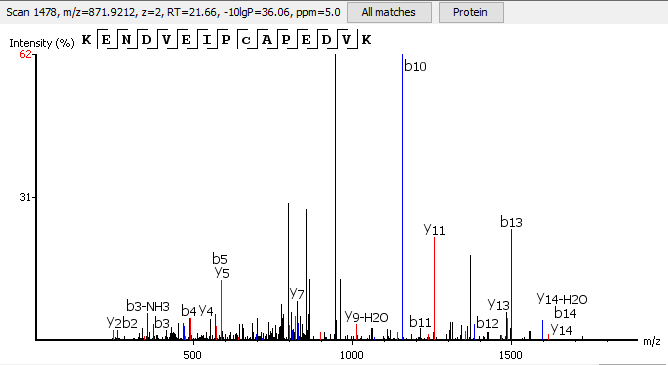
**

**g) JAC96601.1, Snake venom metalloproteinase J, partial**

**
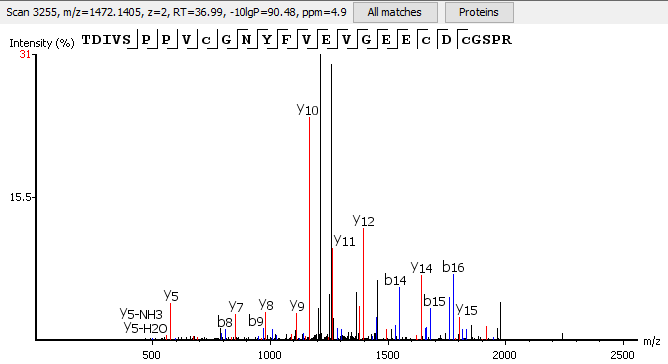
**

**h) ADW54348.1, group III snake venom metalloproteinase**

**
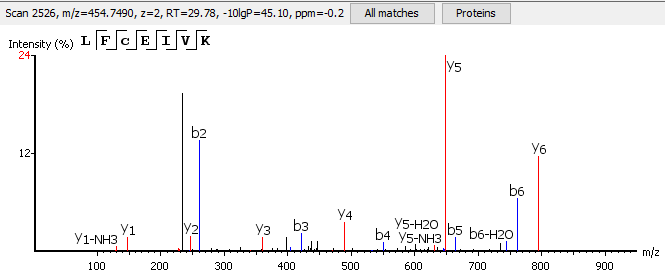
**

**i) ADI47567.1, serine protease, partial**

**
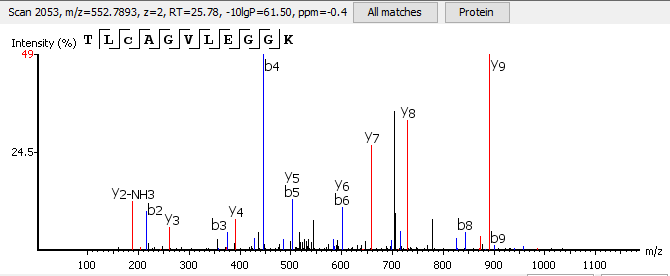
**

**Supplementary figure S4:** Full length un-edited gel image of **figure 3a**. Lanes 1 and 8 contain prothrombin incubated with 1X PBS for 3 h at 37 °C (control). Lanes 2-7 contain products of prothrombin hydrolysis by crude ECV (3 µg/ml) and GF 1-5 (0.5 µg/ml), respectively. Lanes 9-14 contain products of prothrombin hydrolysis by crude ECV (3 µg/ml) and GF 6-10 (0.5 µg/ml), respectively.


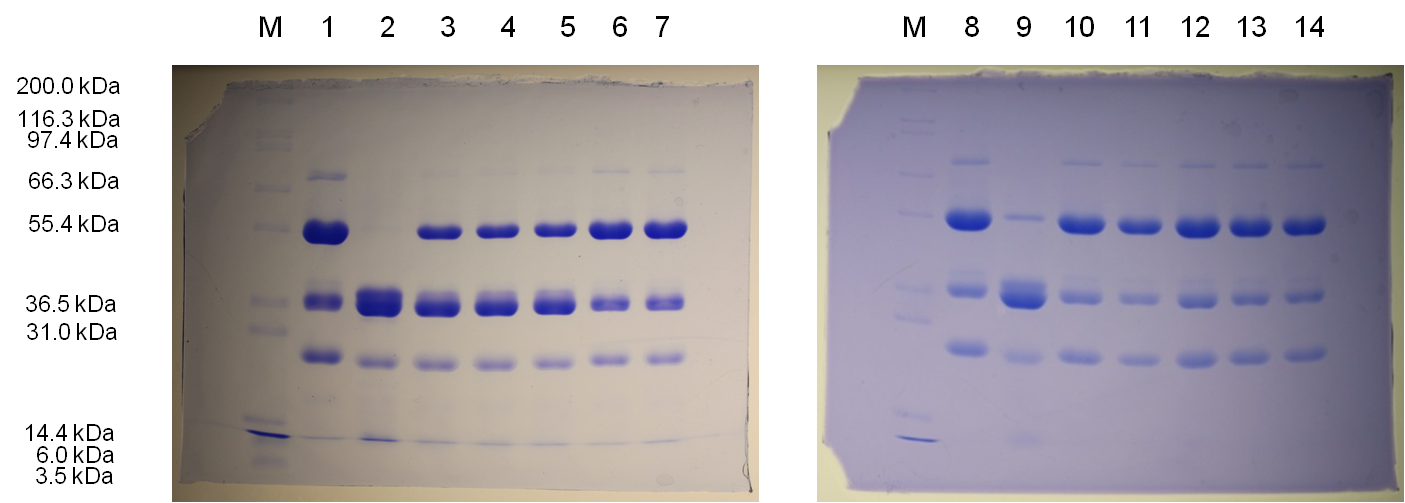


**Supplementary figure S5:** Full length un-edited gel image of **figure 3c**. Lanes 1 and 8 contain fibrinogen incubated with 1X PBS for 3 h at 37 °C (control). Lanes 2-7 contain fibrinogen incubated with ECV (3 µg/ml) and GF 1-5 (0.5 µg/ml), respectively; lanes 9-14 contain fibrinogen incubated with ECV (3 µg/ml) and GF 6-10 (0.5 µg/ml), respectively.

**
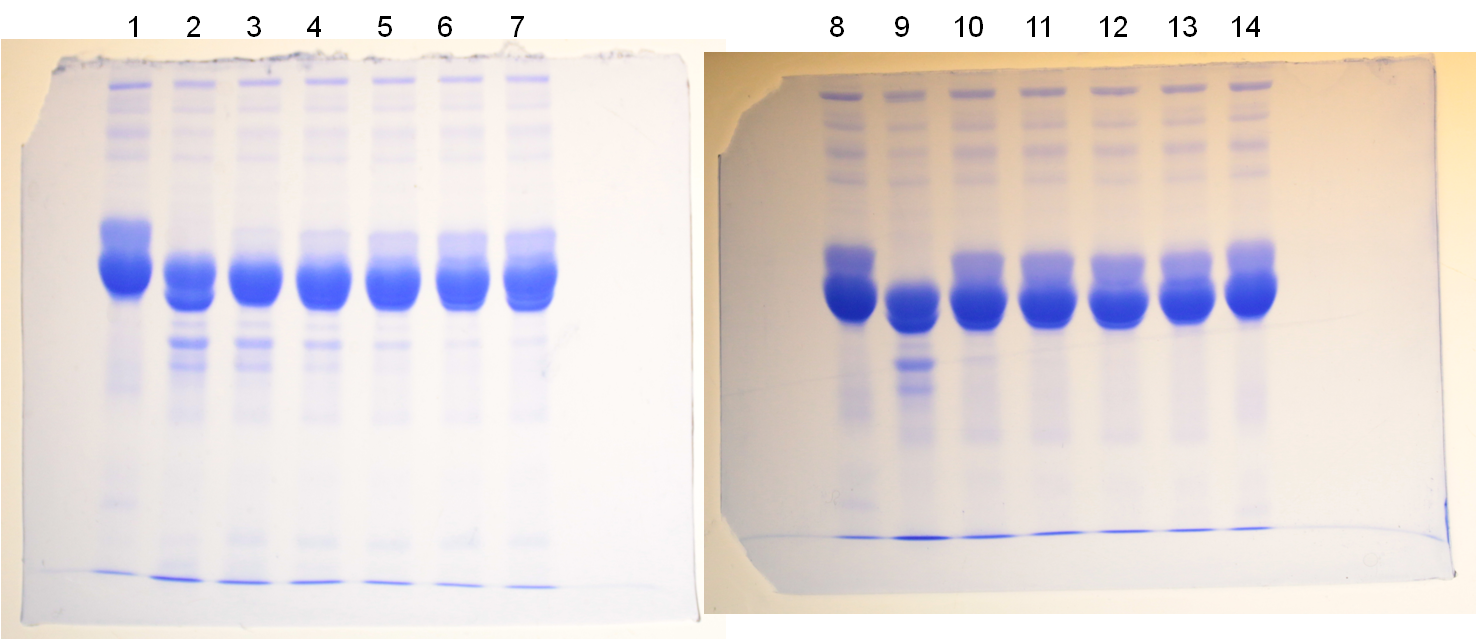
**

**Supplementary Table S1.** List of ions detected in ECV by MALDI-TOF-MS analysis of gel filtration fractions. The analysis was done in the following four m/z ranges: 5 to 20 kDa, 21 to 40 kDa, 41 to 100 kDa, and >100 kDa.

| **m/z ranges** | **m/z of the ions detected in ECV** |
| --- | --- |
| 5 - 20 kDa  (32 entries) | 5030.796, 5973.688, 6253.363, 6485.840, 6715.618, 6899.573, 7067.003, 7448.088, 7560.279, 8009.272, 8142.186, 8309.519, 9001.142, 9925.126, 10224.263, 10869.903, 11183.008, 11388.615,11859.818, 12118.274, 12387.351, 13385.215, 13612.567, 13738.201, 13919.183, 14699.368, 14878.930, 16031.483, 16920.697, 19350.431, 19954.615, 20209.128 |
| 21- 40 kDa  (57 entries) | 20624.049, 21226.845, 21636.645, 21784.312, 22191.261, 22316.228, 23030.931, 23386.306, 23767.319, 23995.830, 24595.317, 24828.813, 24977.040, 25263.072, 25608.543, 25726.380, 26075.416, 26237.328, 26831.176, 27081.348, 27272.203, 27562.353, 28233.071, 28392.300, 28839.105, 29208.035, 29861.621, 30071.268, 30559.411, 30717.427, 31162.640, 31687.830, 32073.876, 32406.373, 32711.003, 33047.222, 33781.109, 34026.521, 34621.276, 35301.941, 35591.798, 35979.553, 36110.888, 36263.347, 36434.801, 36717.771, 37245.461, 37448.942, 37747.955, 37881.684, 38343.302, 38760.858, 38979.202, 39178.828, 39534.397, 39766.017, 40242.983 |
| 41- 100 kDa  (146 entries) | 41407.446, 41654.298, 41828.126, 42076.767, 43113.797, 43388.662, 43630.884, 43875.287, 44041.798, 44319.642, 44464.460, 44742.717, 44867.079, 45358.109, 45564.474, 45730.648, 46153.269, 46425.496, 46628.721, 47032.765, 47504.186, 48092.085, 48430.930, 48652.863, 49579.172, 49740.927, 50108.689, 50609.941, 50800.493, 51191.246, 52240.770, 52601.862, 52957.570, 53309.616, 53971.629, 54183.552, 54714.587, 55099.268, 55222.128, 56093.805, 56222.534, 56524.895, 56763.013, 57232.248, 57447.063, 57865.651, 58106.791, 58424.900, 59019.490, 59301.110, 59548.153, 59793.728, 60001.326, 60764.695, 61358.123, 61963.126, 62250.478, 62796.340, 63142.029, 63274.553, 63687.403, 64655.332, 65019.141, 65328.889, 65991.015, 66472.096, 66678.193, 67212.860, 67460.101, 68246.064, 68399.044, 68717.739, 68885.152, 69608.501, 69885.349, 70241.202, 70803.276, 71199.439, 71652.885, 71836.337, 72036.204, 72319.342, 72734.330, 73222.129, 73388.943, 73548.792, 74199.835, 74661.591, 74894.592, 75025.893, 75329.418, 75534.325, 75824.952, 76321.907, 76743.893, 77012.108, 77308.242, 78062.453, 79191.482, 79536.089, 79925.225, 81110.263, 81492.307, 81611.287, 81779.188, 81962.027, 82598.871, 82829.832, 83487.666, 84359.627, 84562.877, 84995.588, 85197.520, 85412.219, 85787.264, 86020.294, 86737.532, 86860.452, 87500.763, 87866.082, 88970.808, 89269.122, 89398.442, 90257.112, 90747.882, 91181.777, 91632.572, 92081.363, 92272.922, 92388.909, 93212.450, 93615.839, 93906.016, 94646.004, 95299.463, 95825.149, 96072.403, 96649.139, 96962.589, 97290.950, 97624.933, 98090.070, 98205.203, 98856.653, 99257.312, 99853.413 |
| >101 kDa  (72 entries) | 100460.774, 100634.077, 100870.028, 101292.065, 102290.306, 102612.958, 102920.969, 103125.264, 104388.251, 104972.766, 105233.605, 105472.366, 105767.284, 106232.671, 106390.029, 107532.610, 107712.153, 108565.163, 108952.096, 109374.712, 109496.334, 110112.445, 110466.282, 111137.280, 112035.560, 112251.815, 112916.690, 113337.505, 113630.497, 113811.013, 114312.312, 114511.353, 115190.275, 115896.961, 118023.698, 118445.350, 118886.591, 119639.144, 120079.252, 120685.780, 122597.376, 123419.297, 123768.314, 124298.653, 125273.298, 125532.656, 125723.906, 126821.141, 127713.016, 129369.418, 130349.769, 130733.231, 131505.187, 132277.272, 133207.397, 134531.232, 135330.604, 135560.579, 136931.638, 137374.855, 137644.589, 138164.694, 139295.092, 140406.930, 141145.563, 141785.964, 143834.597, 145650.332, 146470.321, 147288.705, 149977.760, 150205.569 |

**Supplementary Table S2.** List of all the proteins identified by LC-MS/MS analysis of gel filtration fractions of ECV against Viperidae venoms protein database. The table shows the identified peptide ions, their mass, charge (z), score for the ID, ΔM (ppm), modified residues. Ca, O, D and PG represent carbamidomethylation, oxidation, Deamidation and Pyro-glu from Q respectively.

| **Accession No.** | **Protein** | **-10lgP** | **Coverage (%)** | **peptide** | **-10lgP** | **Mass (Da)** | **z** | **ppm** | **Modified residues** |
| --- | --- | --- | --- | --- | --- | --- | --- | --- | --- |
| **Enzymatic proteins** | | | | | | | | | |
| **P- I SVMP (1 protein)** | | | | | | | | | |
| gi\|727360735 | Snake venom metalloproteinase K, partial | 102.69 | 8 | (Q)cILnKPLKTDIVSPPVcGnYFVEVGEEcDcGSPR(N) | 28.56 | 3910.8 | 3 | 0 | Ca (C1, C17, C28, C30); D (N4, N19) |
| **P- II SVMP (5 proteins)** | | | | | | | | | |
| gi\|297594086 | metalloproteinase | 114.32 | 34 | (C)KFLNAGTIcK(K) | 35.89 | 1150.6 | 2 | 0.6 | Ca (C9) |
|  |  |  |  | (K)FLNAGTIcKK(A) | 35.45 | 1150.6 | 3 | 0.8 | Ca (C8) |
|  |  |  |  | (R)EGEHcISGPccR(N) | 25.15 | 1460.6 | 3 | 0.2 | Ca (C5, C10, C11) |
|  |  |  |  | (A)mNSVHPccDPVTcEPR(E) | 15.12 | 1973.8 | 3 | 0.7 | O (M1); Ca (C7, C8, C13) |
|  |  |  |  |  |  |  |  |  |  |
| gi\|297593820 | metalloproteinase, partial | 113.74 | 15 | (R)DDcDVPEHcTGQSAEcPR(N) | 79.26 | 2131.8 | 2 | 2.6 | Ca (C3, C9, C16) |
|  |  |  |  | (K)cIFDPPLRND(I) | 27.47 | 1245.6 | 2 | 1.5 | Ca (C1) |
|  |  |  |  | (R)GIFDGYcTK(E) | 27.01 | 1059.5 | 2 | 1.2 | Ca (C7) |
|  |  |  |  |  |  |  |  |  |  |
| gi\|297594078 | metalloproteinase | 100.95 | 9 | (K)RAVGDDmDDYcSGITPDcPR(N) | 43.19 | 2314.9 | 3 | 0.6 | O (M7); Ca (C11, C18) |
|  |  |  |  | (L)qNSANPccDPETcQPK(R | 30.01 | 1887.7 | 2 | 0.1 | PG(Q1); Ca (C7, C8, C13) |
|  |  |  |  |  |  |  |  |  |  |
| gi\|297594068 | metalloproteinase | 98.47 | 31 | (K)FLNAGTIcKR(A) | 48.48 | 1178.6 | 2 | 0.8 | Ca (C8) |
|  |  |  |  | (V)GDNmNDYcTGISSDcPR(N) | 33.37 | 1976.7 | 2 | -0.7 | O (M4); Ca (C8, C15) |
|  |  |  |  |  |  |  |  |  |  |
| gi\|297594070 | metalloproteinase, partial | 77.02 | 8 | (Q)NSANPcYDPLTcHPR(E) | 49.24 | 1800.8 | 2 | 3.2 | Ca (C6, C12) |
| **P- III SVMP (18 proteins)** | | | | | | | | | |
| gi\|297593950 | metalloproteinase | 165.72 | 13 | (R)LYcLDNSPGNKNPcK(M) | 67.92 | 1778.8 | 2 | 1.8 | Ca (C3, C14) |
|  |  |  |  | (K)ARHEcDLPEHcTGQSAEcPIDEFQR(N) | 67.74 | 3041.3 | 3 | 2.5 | Ca (C5, C11, C18, ) |
|  |  |  |  | (C)KLKPGAEcGNGmcCDKcK(I) | 15.13 | 2070.9 | 2 | -0.7 | Ca (C8, C13, C17); O (M12) |
|  |  |  |  |  |  |  |  |  |  |
| gi\|300079900 | factor X activator heavy chain | 153.11 | 11 | (K)LKPGAEcGNGLccYQcK(I) | 65.06 | 2013.9 | 2 | 2.7 | Ca (C7, C12, C13, C,16) |
|  |  |  |  | (R)ARNEcDVPEHcTGQSAEcPR(D) | 48.00 | 2372.0 | 3 | 2.1 | Ca (C5, C11, C18) |
|  |  |  |  | (K)cILYPPLRK(D) | 32.13 | 1158.7 | 3 | 2.9 | Ca (C1) |
|  |  |  |  |  |  |  |  |  |  |
| gi\|297593790 | metalloproteinase, partial | 149.47 | 17 | (R)LYcFDNLPEHK(N) | 59.48 | 1434.7 | 2 | 1.8 | Ca (C3) |
|  |  |  |  | (R)KEnGIKIPcAPEDVK(C) | 53.29 | 1697.9 | 2 | 1.7 | D(N3); Ca (C9) |
|  |  |  |  | (M)GNQcISLFGSR(A) | 44.19 | 1237.6 | 2 | 0.7 | Ca (C4) |
|  |  |  |  | (R)AGTEcRPAKDEcDmADLcNGQSDEcPKDQFQR(N) | 35.41 | 3803.5 | 4 | 1.4 | Ca (C5, C12, C18, C25, ); O (M14) |
|  |  |  |  |  |  |  |  |  |  |
| gi\|297593830 | metalloproteinase, partial | 147.78 | 22 | (R)ATVAEDScFQENLK(G) | 60.96 | 1610.7 | 2 | 1.3 | Ca (C8) |
|  |  |  |  | (R)KASDEcDVPEYcTGQSADcPR(N) | 54.62 | 2444.0 | 2 | 1.4 | Ca (C6, C12, C19) |
|  |  |  |  | (R)LYcLDNSSR(K) | 42.78 | 1126.5 | 2 | 2.4 | Ca (C3) |
|  |  |  |  | (R)KIPcAPQDVK(C) | 37.63 | 1154.6 | 2 | 0.1 | Ca (C4) |
|  |  |  |  | (R)VSDLLNR(K) | 35.87 | 815.5 | 2 | 0.2 |  |
|  |  |  |  |  |  |  |  |  |  |
| gi\|297593798 | metalloproteinase, partial | 145.07 | 25 | (R)KENDVmIPcAPEDVK(R | 57.48 | 1759.8 | 2 | 1.2 | O (M6); Ca (C9) |
|  |  |  |  | (R)nGQPcLNNQGYcYNGK(C) | 56.23 | 1886.8 | 2 | 1.4 | D (N1); Ca (C5, C12) |
|  |  |  |  | (L)HLGVEcESGEccQQGR(F) | 53.78 | 1904.8 | 2 | 1 | Ca (C6, C11, C12) |
|  |  |  |  | (R)LFcEIIKDTcK(Y) | 39.5 | 1425.7 | 2 | -4.4 | Ca (C3, C10) |
|  |  |  |  | (Y)SLFGSIATVGQDGcFEWNK(K) | 36.74 | 2115.0 | 2 | 0.6 | Ca (C14) |
|  |  |  |  |  |  |  |  |  |  |
| gi\|297593862 | metalloproteinase, partial | 130.71 | 14 | (R)ATVAEDAcFQFNSLGSDYGYcR(K) | 59.98 | 2530.1 | 2 | 3.4 | Ca (C8 ,C21) |
|  |  |  |  |  |  |  |  |  |  |
| gi\|162329887 | Chain A, Crystal Structure Of Russell's Viper Venom Metalloproteinase | 124.48 | 13 | (R)DEcDVPEHcTGQSAEcPR(D) | 52.57 | 2145.8 | 2 | 5.4 | Ca (C3, C9, C16) |
| gi\|83523634 | Group III snake venom metalloproteinase | 119.25 | 15 | (R)TDIVSPPVcGnDLLEKGEEcDCGSPENcQnPccDAASCKL(H) | 26.18 | 4442.8 | 3 | -0.9 | Ca (C9, C20, C28,C32, C33) ; D (N11, N30, ) |
|  |  |  |  | (R)NEcDLPEYcTGQSAECPIDRSHRn(G) | 17.02 | 2851.2 | 3 | -5.8 | Ca (C3, C9); D (N24) |
|  |  |  |  |  |  |  |  |  |  |
| gi\|297593842 | metalloproteinase, partial | 116.46 | 26 | (R)TDIVSPPVcGNDLLER(G) | 32.14 | 1783.9 | 2 | 2.4 | Ca (C9) |
|  |  |  |  | (R)NEcDLPEYcTGQSGDcPTDHFHR(N) | 24.98 | 2794.1 | 2 | 9.8 | Ca (C3, C9, C16) |
|  |  |  |  |  |  |  |  |  |  |
| gi\|297593794 | metalloproteinase | 115.08 | 7 | (R)KEnDVPVPcAQEDVK(C) | 67.62 | 1727.8 | 2 | 3.7 | D (N3); Ca (C9) |
|  |  |  |  |  |  |  |  |  |  |
| gi\|297593836 | metalloproteinase, partial | 107.73 | 9 | (K)LPIDNTPLcNYR(Y) | 64.46 | 1474.7 | 2 | 1.3 | Ca (C9) |
|  |  |  |  | (R)SADcPTDYFHR(N) | 54.86 | 1367.6 | 2 | 1.8 | Ca (C4) |
|  |  |  |  | (R)FATAETVcRPAK(S) | 47.5 | 1349.7 | 2 | 0.2 | Ca (C8) |
|  |  |  |  |  |  |  |  |  |  |
| gi\|297593852 | metalloproteinase, partial | 103.55 | 10 | (K)KAESYFYcR(K) | 47.77 | 1222.5 | 2 | 0.1 | Ca (C8) |
|  |  |  |  | (R)SEcDLPEYcTGQSADcPTDHFHR(N) | 46.64 | 2781.1 | 2 | 1.4 | Ca (C3, C9, C16) |
|  |  |  |  |  |  |  |  |  |  |
| gi\|320579375 | group III snake venom metalloproteinase, partial | 103.03 | 9 | (R)YPccDAAScK(L) | 41.66 | 1230.4 | 2 | 0.2 | Ca (C3, C4, C9) |
|  |  |  |  | (R)TDIISPPVcGNDLLEK(G) | 34.36 | 1769.9 | 2 | 1 | Ca (C9) |
|  |  |  |  |  |  |  |  |  |  |
| gi\|297593822 | metalloproteinase, partial | 99.09 | 11 | (V)PLVGMEFWNQR(D) | 70.45 | 1474.7 | 2 | 3.9 |  |
|  |  |  |  | (R)TDIVSPPVcGNALLEK(G) | 40.85 | 1711.9 | 2 | 2.1 | Ca (C9) |
|  |  |  |  |  |  |  |  |  |  |
| gi\|297593854 | metalloproteinase | 91.01 | 5 | (E)NCQDPccDAALcK(L) | 34.03 | 1553.6 | 2 | 0.2 | Ca (C6, C7, C12) |
|  |  |  |  |  |  |  |  |  |  |
| gi\|297593858 | metalloproteinase | 72.99 | 5 | (T)YcQNPccDAATcK(L) | 47 | 1646.6 | 2 | 0.7 | Ca (C2, C6, C7, C12) |
|  |  |  |  | (R)HcVDVNTAY- | 33.05 | 1077.5 | 2 | -0.1 | Ca (C2) |
|  |  |  |  |  |  |  |  |  |  |
| gi\|52000738 | Hemorrhagic metalloproteinase HT-1 | 62.04 | 7 | (R)KEnGEKIPcAPEDVK(C) | 31.44 | 1713.8 | 2 | 1.3 | D (N3); Ca (C9) |
|  |  |  |  |  |  |  |  |  |  |
| gi\|297593984 | metalloproteinase, partial | 53.13 | 5 | (D)IVSPPVcGNELLEEGEEcDcGSR(A) | 23.32 | 2605.1 | 3 | 0.2 | Ca (C7, C18, C20) |
|  |  |  |  |  |  |  |  |  |  |
| **PLA_2_ (12 proteins)** | | | | | | | | | |
| gi\|82096307 | Acidic phospholipase A2 EC-I | 178.05 | 59 | (K)LPILSYGSYGCYCGWGGQGPPK(D) | 77.44 | 2416.098 | 2 | -0.2 | Ca (C11, C13) |
|  |  |  |  | (R)AVCECDREAAICLGENVNTYDKK(Y) | 61.41 | 2714.21 | 3 | 0.9 | Ca (C3,C5,C12) |
|  |  |  |  | (R)CCLVHDCCYTR(V) | 48.43 | 1542.584 | 2 | 1.1 | Ca (C1 , C2, C7, C8) |
|  |  |  |  | (G)NLYQFGR(M) | 40.62 | 896.4504 | 2 | 0.4 |  |
|  |  |  |  |  |  |  |  |  |  |
| gi\|87130858 | phospholipase A2-III | 83.27 | 27 | (R)AAAICLGQNVNTYDK(N) | 56.19 | 1636.788 | 2 | 1 | Ca (C5) |
|  |  |  |  | (R)CCFVHDCCYGTVNDCNPK(M) | 54.14 | 2304.848 | 3 | 1.2 | Ca (C1,C2,C7,C8,C15) |
|  |  |  |  |  |  |  |  |  |  |
| gi\|59727030 | D1E6b phospholipase A2 | 68.37 | 10 | (K)QVCECDKAAAICFR(D) | 44.61 | 1726.759 | 2 | 1 | Ca (C3, C5, C12) |
|  |  |  |  |  |  |  |  |  |  |
|  |  |  |  |  |  |  |  |  |  |
| gi\|1041577423 | Phospholipase A2 1h | 60.88 | 6 | (D)GAAAICFR(D) | 29.64 | 864.4276 | 2 | 0.6 | Ca (C6) |
|  |  |  |  |  |  |  |  |  |  |
|  |  |  |  |  |  |  |  |  |  |
| gi\|298351762 | Basic phospholipase A2 | 198.21 | 69 | (K)RVNGAIVCEQGTSCENRICECDK(K) | 53.87 | 2754.194 | 3 | 2 | Ca (C8, C14, C19) |
|  |  |  |  | (R)VNGAIVCEQGTSCENRICECDKAAAICFR(R) | 34.93 | 3387.489 | 3 | 2 | Ca (C7, C13, C18, C20, C27) |
|  |  |  |  | (K)IYMLYPDFLCK(E) | 25.19 | 1477.699 | 2 | 1.6 | O (M3); Ca (C10) |
|  |  |  |  |  |  |  |  |  |  |
| gi\|81174981 | Basic phospholipase A2 | 183.48 | 46 | (K)LAVPFYSSYGCYCGWGGKG(T) | 36.74 | 2127.918 | 2 | 2.5 | Ca (C11, C13) |
|  |  |  |  |  |  |  |  |  |  |
| gi\|71912229 | phospholipase A2 | 183.45 | 75 | (R)VNGAIVCEKGTSCENR(I) | 60.41 | 1792.82 | 2 | 1.2 | Ca(C7,C13) |
|  |  |  |  | (R)ICECDKAAAICFRQNLNTYSK(K) | 26.85 | 2561.182 | 4 | 1.5 | Ca(C2,C4) |
|  |  |  |  | (E)GSLLEFGK(M) | 26.7 | 849.4596 | 2 | 0.5 |  |
|  |  |  |  | (K)KYMLYPDFLCKGELKC- | 15.95 | 2079.983 | 3 | 0.7 | O (M3); Ca(C10,C16) |
|  |  |  |  |  |  |  |  |  |  |
| gi\|149243451 | Chain A, Crystal Structure Of A New Isoform Of Phospholipase A2 | 121.82 | 60 | -SLIEFGK(M) | 32.51 | 792.4381 | 2 | 0.8 |  |
|  |  |  |  | (K)DATDRCCFVHDCCYGNLPDNPK(S) | 28.59 | 2873.108 | 3 | 1.2 | Ca (C6, C7, C12, C13) |
|  |  |  |  | (K)LAIPSYSSYGCYCGGGGKGTPKDA(T) | 15.38 | 2465.099 | 2 | 8.9 | Ca (C11,C13) |
|  |  |  |  |  |  |  |  |  |  |
| gi\|163311140 | Chain B, Crystal Structure Of Ecarpholin S Complexed With Suramin | 69.3 | 25 | (R)ccLAHSccYDTLPDcSPK(T) | 56.31 | 2242.857 | 2 | 1.5 | Ca (C1, C2, C7, C8, C15) |
|  |  |  |  | (E)NGEIIcENSTScK(K) | 25.97 | 1510.639 | 2 | 0.6 | Ca (C6, C12) |
|  |  |  |  |  |  |  |  |  |  |
|  |  |  |  |  |  |  |  |  |  |
| gi\|1041577114 | Phospholipase A2 1h | 63.46 | 10 | (K)QICECNRAAAIcFR(D) | 34.01 | 1653.754 | 3 | -6.1 | Ca (C12) |
|  |  |  |  |  |  |  |  |  |  |
| gi\|71912223 | basic phospholipase A2 | 43.99 | 13 | (K)NPLSSYSNYGcYcGWGGK(G) | 43.99 | 2068.841 | 2 | 1.8 | Ca (C11, C13) |
|  |  |  |  |  |  |  |  |  |  |
| gi\|157834128 | Chain A, Anticoagulant Class Ii Phospholipase A2 | 33.79 | 25 | (M)TGKnPLSSYSDYGcYCGWGGKGKPQDATDR(C) | 33.79 | 3268.419 | 3 | -3.4 | D (N4); Ca (C14) |
|  |  |  |  |  |  |  |  |  |  |
| **SVSP (11 proteins)** | | | | | | | | | |
| gi\|297593766 | serine protease, partial | 76.47 | 8 | (R)TFcAGVLQGGK(D) | 56.31 | 1136.6 | 2 | -0.1 | Ca (C3) |
|  |  |  |  | (R)AAYPWLPVNSR(T) | 40.31 | 1272.7 | 2 | 0.5 |  |
|  |  |  |  |  |  |  |  |  |  |
| gi\|293491172 | serine protease, partial | 73.07 | 14 | (R)TLcAGILR(G) | 39.68 | 902.5 | 2 | -0.2 | Ca (C3) |
|  |  |  |  | (K)SSPHIAPISLPSNPPR(L) | 21.76 | 1668.89 | 2 | -9.2 |  |
|  |  |  |  |  |  |  |  |  |  |
| gi\|306756038 | serine protease VLSP-3 precursor | 66.96 | 14 | (K)TSTHIAPLSLPSSPPSVGSVcR(I) | 47.58 | 2249.1 | 3 | 0.2 | Ca (C21) |
|  |  |  |  | (L)VIGGDEcNInEHR(S) | 38.74 | 1512.7 | 2 | 0.4 | Ca (C7); D (N10) |
|  |  |  |  |  |  |  |  |  |  |
| gi\|13959655 | Venom serine proteinase-like protein 2 | 57.74 | 6 | (R)TLcAGILQGGIDScK(V) | 57.74 | 1591.8 | 2 | 1.1 | Ca (C3,C13) |
|  |  |  |  |  |  |  |  |  |  |
| gi\|297593786 | serine protease, partial | 56.82 | 5 | (K)YFcLPR(R) | 34.27 | 854.4 | 2 | 0.3 | Ca (C3) |
|  |  |  |  |  |  |  |  |  |  |
| gi\|306756034 | serine protease VLSP-1 precursor | 54.18 | 6 | (P)FSLPSSPPTVGSVcR(I) | 54.18 | 1589.8 | 2 | 1.4 | Ca (C13) |
|  |  |  |  |  |  |  |  |  |  |
| gi\|297593736 | serine protease, partial | 51.71 | 8 | (R)ETYFcLPSR(N) | 42.42 | 1171.5 | 2 | 0.4 | Ca (C5) |
|  |  |  |  | (R)TLCAGILGR(R) | 18.58 | 902.5 | 2 | -0.4 |  |
|  |  |  |  |  |  |  |  |  |  |
| gi\|311223824 | serine beta-fibrinogenase-like protein precursor | 91.84 | 11 | (K)TSTYIAPLSLPSSPPR(V) | 51.7 | 1685.9 | 2 | 1.2 |  |
|  |  |  |  | (R)NNAEIRLPEER(F) | 21.91 | 1339.7 | 2 | 1.9 |  |
|  |  |  |  |  |  |  |  |  |  |
| gi\|134129 | Factor V activator RVV-V alpha | 115.68 | 34 | (K)WcEPLYPWVPADSR(T) | 52.15 | 1774.8 | 2 | 1 | Ca (C2) |
|  |  |  |  | (R)EWVLTAAHcDR(R) | 43.24 | 1356.6 | 2 | 0.1 | Ca (C9) |
|  |  |  |  | (R)TLcAGILK(G) | 35.54 | 874.5 | 2 | 0.5 | Ca (C3) |
|  |  |  |  | (K)ISTTEDTYPDVPHcTNIFIVK(H) | 30.64 | 2449.2 | 3 | 1.2 | Ca (C13) |
|  |  |  |  | (K)YFcLNTK(F) | 29.21 | 944.4 | 2 | 0.7 | Ca (C3) |
|  |  |  |  | (R)LRRPVTYSTHIAPVSLPSR(S) | 27.35 | 2149.2 | 3 | 0.7 |  |
|  |  |  |  |  |  |  |  |  |  |
| gi\|297593764 | serine protease, partial | 63.79 | 10 | (K)IFDYSVcR(G) | 49.68 | 1058.5 | 2 | 0.9 | Ca (C6) |
|  |  |  |  | (R)TLcAGVLEGGIDScK(A) | 28.2 | 1578.7 | 2 | 1 | Ca (C3, C14) |
|  |  |  |  |  |  |  |  |  |  |
| gi\|298351881 | Snake venom serine protease rhinocerase | 53.95 | 28 | (R)TLcAGVLEGGK(D) | 40.07 | 1103.6 | 2 | 0.3 | Ca (C3) |
|  |  |  |  | (A)HPcAQPLLPAFYTK(V) | 27.75 | 1641.8 | 2 | 1.2 | Ca (C3) |
|  |  |  |  |  |  |  |  |  |  |
| **LAAO (3 proteins)** | | | | | | | | | |
| gi\|194400545 | secreted L-amino acid oxidase precursor | 212.0 | 29 | (K)KDLQTFcYPSIIQK(W) | 75.6 | 1739.9 | 2 | 2.2 | Ca (C7) |
|  |  |  |  | (K)SAGQLYQESLGK(A) | 70.4 | 1279.6 | 2 | 1.8 |  |
|  |  |  |  | (K)KFWEDDGIQGGK(S) | 56.8 | 1378.7 | 2 | 1.3 |  |
|  |  |  |  | (S)GLSAAYVLAGAGHK(V) | 54.7 | 1313.7 | 2 | 0.8 |  |
|  |  |  |  | (K)LNEFVQETEnGWYFIKN(I) | 48.4 | 2130.0 | 2 | 2.9 | D (N10) |
|  |  |  |  | (K)VTVTYQTTQK(N) | 42.2 | 1167.6 | 2 | 1.9 |  |
|  |  |  |  | (R)AIEESVHFK(A) | 38.1 | 1058.5 | 2 | 1.6 |  |
|  |  |  |  | (K)VTVLEASERPGGR(V) | 33.7 | 1369.7 | 3 | 1.5 |  |
|  |  |  |  | (S)LKHDDIFAYEK(R) | 30.3 | 1377.7 | 3 | 0.8 |  |
|  |  |  |  | (C)ADDKNPLEEcFREDDYEEFLEIAK(N) | 25.5 | 2974.3 | 3 | 2.6 | Ca (C10) |
|  |  |  |  | (R)ITFKPPLPPKK(A) | 24.8 | 1264.8 | 4 | 1.1 |  |
|  |  |  |  |  |  |  |  |  |  |
| gi\|727360693 | L-amino acid oxidase B variant 2 | 115.2 | 15 | (R)EADYEEFLEIAR(N) | 40.7 | 1483.7 | 2 | 0.5 |  |
|  |  |  |  |  |  |  |  |  |  |
|  |  |  |  |  |  |  |  |  |  |
| gi\|1002590400 | PREDICTED: L-amino-acid oxidase isoform X1 | 104.8 | 8 | (Y)TANAHGWIDSTIK(S) | 50.0 | 1412.7 | 2 | -0.4 |  |
|  |  |  |  | (C)ADNKNPLEEcFR(E | 40.4 | 1491.7 | 3 | -0.8 | Ca (C10) |
|  |  |  |  | (K)IFLTcTK(K) | 26.2 | 881.5 | 2 | 0.3 | Ca (C5) |
| **PDE (1 protein)** | | | | | | | | | |
| gi\|586829527 | phosphodiesterase | 124.14 | 8 | (K)VLSFILPHRPDNSEScADTSPDNLWVEER(I) | 71.06 | 3382.6 | 3 | 4.6 | Ca (C16) |
| **NT (1 protein)** | | | | | | | | | |
| gi\|586829529 | 5'-nucleotidase, partial | 119.06 | 23 | (K)IQLQNYYSQEIGK(T) | 51.31 | 1582.799 | 2 | -0.6 |  |
|  |  |  |  | (R)VVSLNVLcTK(C) | 46.79 | 1131.632 | 2 | -0.6 | Ca (C8) |
|  |  |  |  | (K)VGIIGYTTK(E) | 38.06 | 950.5436 | 2 | 0.7 |  |
|  |  |  |  | (T)GELLQVSGIK(V) | 28.37 | 1042.602 | 2 | 0.2 |  |
|  |  |  |  | (K)VFPAVEGR(V) | 24.13 | 873.4708 | 2 | 0.5 |  |
|  |  |  |  | (K)VVYDLSQKPGSR(V) | 21.17 | 1347.715 | 3 | -0.5 |  |
|  |  |  |  | (K)LTTLGVNK(I) | 19.79 | 844.5018 | 2 | -0.6 |  |
|  |  |  |  |  |  |  |  |  |  |
| **GC (1 protein)** | | | | | | | | | |
| gi\|380846517 | glutaminyl-peptide cyclotransferases | 84.26 | 6 | (Q)HPVEDDHIPFLR(R) | 59.72 | 1473.7 | 3 | 2 |  |
|  |  |  |  | (R)YPGSPGSYAVR(Q) | 49.09 | 1152.6 | 2 | 2.5 |  |
| **ASPro (1 protein)** | | | | | | | | | |
| gi\|109287596 | renin-like aspartic protease | 66.41 | 5 | (R)YDSSESSTYKPK(G) | 48.61 | 1390.6 | 2 | 0.8 |  |
|  |  |  |  | (N)VFSVYYSR(H) | 35.58 | 1019.5 | 2 | 0 |  |
|  |  |  |  |  |  |  |  |  |  |
| **APase (1 protein)** | | | | | | | | | |
| gi\|743538216 | endoplasmic reticulum aminopeptidase 1-like protein | 86.52 | 5 | (K)AYIVNLFK(N) | 48.26 | 966.6 | 2 | 0.8 |  |
|  |  |  |  | (Y)FGIAYPLPK(Q) | 41.18 | 1004.6 | 2 | 1.4 |  |
|  |  |  |  | (R)LLKDNHETISSNDR(A) | 31.28 | 1640.8 | 3 | 0.6 |  |
|  |  |  |  | (S)SSAGSLWHIPLTYITNK(C) | 28.98 | 1887.0 | 3 | 1 |  |
| **PLB (1 protein)** | | | | | | | | | |
| gi\|727360709 | Phospholipase B | 80.51 | 6 | (R)HGYWPSYNIPFHK(V) | 50 | 1644.8 | 3 | 2.2 |  |
|  |  |  |  | (K)TWAQIFEK(E) | 42.67 | 1021.5 | 2 | 2.1 |  |
|  |  |  |  | (K)QVVPESLFAWER(V) | 27.51 | 1459.7 | 2 | 3.6 |  |
| **Non enzymatic proteins** | | | | | | | | | |
| **Snaclec (20 proteins)** | | | | | | | | | |
| gi\|300490474 | P68 alpha subunit | 122.92 | 23 | (K)TPADYVWIGLR(N) | 64.55 | 1289.7 | 2 | 3.3 |  |
|  |  |  |  |  |  |  |  |  |  |
| gi\|758377456 | C-type lectin-like protein 3A | 115.87 | 37 | (R)TWFNLScGDDYPFVcK(F) | 65.1 | 2007.8 | 2 | 5.9 | Ca (C7,C15) |
|  |  |  |  | (K)FcTEQANGGHLVSIESV(E) | 37.53 | 1846.9 | 2 | 7.6 | Ca (C2) |
|  |  |  |  | (K)WTDGSSVIYK(N) | 24.44 | 1154.6 | 2 | -0.3 |  |
|  |  |  |  | (V)EEAEFVAQLVSENIK(T) | 15.05 | 1704.9 | 2 | 2.7 |  |
|  |  |  |  |  |  |  |  |  |  |
| gi\|758377450 | C-type lectin-like protein 3B | 94.95 | 26 | (A)AFccPSGWSAYDQNcYK(V) | 69.3 | 2112.8 | 2 | 1.9 | Ca (C3,C4,C15) |
|  |  |  |  | (K)AWNEGTNcFVFK(I) | 24.1 | 1471.7 | 2 | 2.4 | Ca (C8) |
|  |  |  |  |  |  |  |  |  |  |
|  |  |  |  |  |  |  |  |  |  |
| gi\|55670410 | Chain B, Structure Of Ems16-Alpha2-I Domain Complex | 123.97 | 45 | (R)LASIHSSEEEAFVSK(L) | 66.22 | 1632.8 | 2 | 0.6 |  |
|  |  |  |  | -cPLGWSSFDQHcYK(V) | 55.86 | 1783.7 | 2 | 0.2 | Ca (C1, C12) |
|  |  |  |  | (K)WEWSDNAR(F) | 40.42 | 1062.5 | 2 | -0.3 |  |
|  |  |  |  | (R)IFWFTR(G) | 33.5 | 868.5 | 2 | -0.5 |  |
|  |  |  |  | (K)SVSFVcK(F) | 25.08 | 825.4 | 2 | -0.1 | Ca (C6) |
|  |  |  |  | (K)FLTDPAV- | 17.63 | 761.4 | 2 | -0.9 |  |
|  |  |  |  |  |  |  |  |  |  |
| gi\|38493055 | Chain A, Crystal Structure Of Ems16, An Antagonist Of Collagen Receptor Integrin Alpha2beta1 (GpiaIIA) | 114.95 | 49 | (R)REEQQcNPEWNDGSK(I) | 50.36 | 1875.8 | 2 | 0.6 | Ca (C6) |
|  |  |  |  | (A)IGEPQNWYEAER(F) | 44.88 | 1490.7 | 2 | 0 |  |
|  |  |  |  | (K)DGHLVSIQSR(E) | 34.69 | 1110.6 | 2 | 0 |  |
|  |  |  |  | (K)WTNFHDWNNINcEDLYPFVcK(F) | 30.74 | 2771.2 | 3 | 0.6 | Ca (C12, C20) |
|  |  |  |  | (K)IIYVNWK(E) | 30.73 | 934.5 | 2 | -0.5 |  |
|  |  |  |  |  |  |  |  |  |  |
| gi\|300490472 | P68 beta subunit | 110.56 | 32 | (K)GSHLLSLHNIAEADFVLKK(T) | 46.83 | 2091.1 | 4 | -0.8 |  |
|  |  |  |  | (W)GWTDGAKLDYK(A) | 16.03 | 1252.6 | 2 | 1.4 |  |
|  |  |  |  |  |  |  |  |  |  |
| gi\|998226344 | C-type lectin 2 | 93.67 | 32 | (A)EKFCVEqAGHLASIESQEEADFVAQLVSEnVK(S) | 44.94 | 3535.7 | 3 | 2.4 | D (Q7, N30) |
|  |  |  |  | (K)NcFGLEK(E) | 25.48 | 866.4 | 2 | -0.3 | Ca (C2) |
|  |  |  |  |  |  |  |  |  |  |
|  |  |  |  |  |  |  |  |  |  |
| gi\|82175557 | Snaclec salmorin subunit A; Flags: Precursor | 53.46 | 6 | (H)KWVNVYCGqR(N) | 23.33 | 1252.6 | 3 | 0.5 | D (Q9) |
|  |  |  |  |  |  |  |  |  |  |
| gi\|300490478 | P31 alpha subunit | 52.92 | 14 | (K)FcTEQANSGHLVSIK(S) | 37.55 | 1689.8 | 3 | 0.2 | Ca (C2) |
|  |  |  |  |  |  |  |  |  |  |
| gi\|205275155 | C-type lectin | 36.75 | 9 | (K)FcTEQANGGHLAS(F) | 36.75 | 1390.6 | 2 | -0.5 | Ca (C2) |
|  |  |  |  |  |  |  |  |  |  |
|  |  |  |  |  |  |  |  |  |  |
| gi\|538259843 | C-type lectin factor IX/X binding protein B subunit, partial | 43.47 | 8 | (S)SEEADFVVK(L) | 43.47 | 1022.5 | 2 | 1.8 |  |
|  |  |  |  |  |  |  |  |  |  |
| gi\|578004418 | lebecin B, partial | 52.69 | 18 | (K)FcTQqAnGGHLVSIESV(E) | 36.42 | 1847.8 | 2 | 8.2 | Ca (C2); D (Q5, N7) |
|  |  |  |  |  |  |  |  |  |  |
| gi\|727360769 | C-type lectin J | 116.83 | 23 | (K)SSADYVWIGLWNK(R) | 71.53 | 1537.8 | 2 | 2.7 |  |
|  |  |  |  | (R)TWFNLR(C) | 22.06 | 835.4 | 2 | 0.4 |  |
|  |  |  |  |  |  |  |  |  |  |
| gi\|802148 | echicetin beta subunit=inhibitor of von Willebrand factor and thrombin-glycoprotein Ib binding | 104.7 | 31 | (R)DcYWEWSDGAQLDYK(A) | 62.29 | 1934.8 | 2 | 2.8 | Ca (C2) |
|  |  |  |  | _-ncLPDWSVYEGYcYK(V) | 59.92 | 1953.8 | 2 | 5.2 | D (N1); Ca (C2, C13) |
|  |  |  |  |  |  |  |  |  |  |
| gi\|32452854 | echicetin A-chain | 103.17 | 31 | (K)TWDEAEKFcNK(Q) | 59.9 | 1426.6 | 2 | 1.4 | Ca (C9) |
|  |  |  |  | (R)SYEIAIR(Y) | 41.29 | 850.5 | 2 | 0.6 |  |
|  |  |  |  | (K)IWTGLSER(S) | 39.54 | 960.5 | 2 | 0.5 |  |
|  |  |  |  | (K)YPVPR- | 22.56 | 630.3 | 2 | 0.6 |  |
|  |  |  |  | (R)YSEcFVLEK(Q) | 19.01 | 1173.5 | 2 | -5.4 | Ca (C4) |
|  |  |  |  |  |  |  |  |  |  |
| gi\|40889261 | Chain B, Crystal Structure Of Echicetin From The Venom Of Indian Saw- Scaled Viper At 2.4 Resolution | 101.88 | 29 | (K)NDLVWIGLTDYWR(D) | 59.04 | 1649.8 | 2 | 1.1 |  |
|  |  |  |  | (K)DGHLVSFR(N) | 37.32 | 929.5 | 2 | 0.4 |  |
|  |  |  |  |  |  |  |  |  |  |
| gi\|300490462 | dabocetin alpha subunit | 86.53 | 27 | (K)YHEWITLPcGDKNPFIcK(S) | 44.53 | 2277.1 | 3 | 0.7 | Ca (C9, C17) |
|  |  |  |  | (A)DcPSDWSSHEGHcYK(V) | 43.04 | 1863.7 | 2 | 0.8 | Ca (C2, C13) |
|  |  |  |  |  |  |  |  |  |  |
| gi\|218526484 | Snaclec A13; | 77.52 | 15 | (K)HLATIEWLGK(A) | 56.09 | 1166.6 | 2 | 0.7 |  |
|  |  |  |  | (K)TWEDAEKFcQ(K) | 42.85 | 1312.5 | 2 | 0.5 | Ca (C9) |
|  |  |  |  |  |  |  |  |  |  |
| gi\|2829697 | Snaclec echicetin subunit alpha | 36.05 | 14 | -DQDcLSGWSFYEGHCYqL(F) | 36.05 | 2207.9 | 2 | 6.7 | Ca (C4); D (Q17) |
|  |  |  |  |  |  |  |  |  |  |
| gi\|82174836 | Snaclec coagulation factor IX/factor X-binding protein subunit B; Short=IX/X-BP subunit B; AltName: Full=ECLV IX/X-BP subunit B | 34.43 | 7 | (A)nGGHLVSFR(S) | 34.43 | 986.5 | 2 | -0.6 | D (N1) |
|  |  |  |  |  |  |  |  |  |  |
| **Disintegrin (6 proteins)** | | | | | | | | | |
| gi\|182705265 | Disintegrin schistatin-like subunit B | 104.43 | 52 | -NSVNPCCDPQTCKPIE(E) | 27.77 | 1917.8 | 2 | 1 | Ca (C6, C7, C12) |
|  |  |  |  |  |  |  |  |  |  |
| gi\|82194569 | Disintegrin | 151.29 | 89 | (K)RAmLDGLHDYcTGVTSDcPR(N) | 47.6 | 2339.0 | 3 | 1.5 | O (M3); Ca (C11, C18) |
|  |  |  |  | -NSVHPccDPVKcEPR(E) | 46.7 | 1853.8 | 3 | 0.9 | Ca (C6, C7, C12) |
|  |  |  |  | (C)KFLNAGTIcK(R) | 35.89 | 1150.6 | 2 | 0.6 | Ca (C9) |
|  |  |  |  | (R)EGEHcISGPccR(N) | 25.15 | 1460.6 | 3 | 0.2 | Ca (C5, C10 ,C11) |
|  |  |  |  |  |  |  |  |  |  |
|  |  |  |  |  |  |  |  |  |  |
| gi\|544584743 | Disintegrin EC6 subunit alpha; | 114.32 | 34 | (K)FLNAGTIcKK(A) | 35.45 | 1150.6 | 3 | 0.8 | Ca (C8) |
|  |  |  |  | (A)MnSVHPccDPVTcEPR(E) | 15.12 | 1973.8 | 3 | 0.7 | O (M2); Ca (C7, C8, C12) |
|  |  |  |  |  |  |  |  |  |  |
|  |  |  |  |  |  |  |  |  |  |
| gi\|82203514 | =Disintegrin gabonin-1 | 94.79 | 18 | (D)YcTGVTPDcPR(N) | 22.13 | 1324.6 | 2 | 1.6 | Ca (C2, C9) |
|  |  |  |  | (K)FLNAGTIcKRG(R) | 20.81 | 1235.6 | 3 | 0.6 | Ca (C8) |
|  |  |  |  |  |  |  |  |  |  |
|  |  |  |  |  |  |  |  |  |  |
| gi\|182705262 | Disintegrin VLO4 | 91.67 | 46 | (R)ARGDDmnDYcTGISPDcPR(N) | 26.3 | 2215.9 | 2 | -3 | O (M6); D (N7); Ca (C10,C17) |
|  |  |  |  |  |  |  |  |  |  |
|  |  |  |  |  |  |  |  |  |  |
| gi\|110346540 | RTS-containing short disintegrin, partial | 46.91 | 23 | (K)LKPAGTTcWR(T) | 46.91 | 1188.6 | 2 | 1.1 | Ca (C8) |
|  |  |  |  |  |  |  |  |  |  |
| **VEGF (1 protein)** | | | | | | | | | |
| gi\|48429241 | Snake venom vascular endothelial growth factor toxin ICPP; | 57.74 | 10 | -qVRPFPDVYQR(S) | 57.74 | 1386.7 | 2 | 1.7 | PG (Q1) |
|  |  |  |  |  |  |  |  |  |  |
|  |  |  |  |  |  |  |  |  |  |
| **CRISP (3 proteins)** | | | | | | | | | |
| gi\|190195321 | cysteine-rich seceretory protein Dr-CRPK | 125.59 | 22 | (R)cILNHSPYNSR(V) | 54.69 | 1359.6 | 2 | 0 | C1 (Ca) |
|  |  |  |  | (K)TKcPAScFcHNEII_ | 38.34 | 1735.7 | 2 | 2.3 | Ca (C3,C7,C9) |
|  |  |  |  | (S)GSVDFDSESPR(R) | 36 | 1194.5 | 2 | 0.8 |  |
|  |  |  |  | (R)RRPEIQNEIVDLHNSLR(R) | 26.37 | 2088.1 | 4 | 0 |  |
|  |  |  |  |  |  |  |  |  |  |
| gi\|803374854 | RecName: Full=Cysteine-rich venom protein | 116.84 | 19 | -NVDFDSESPR(K) | 54.71 | 1164.5 | 2 | 0.4 |  |
|  |  |  |  | (K)SNcAAScFcHSEIK- | 48.15 | 1669.7 | 2 | -0.1 | Ca (C3,C7,C9) |
|  |  |  |  |  |  |  |  |  |  |
|  |  |  |  |  |  |  |  |  |  |
| gi\|1041577503 | Cysteine-rich secretory protein 1 | 84.05 | 12 | (R)KPEIqNQIVDLHNSLRR(S) | 26.52 | 2060.1 | 3 | -1.1 | D (Q5) |
| **KSPI (3 proteins)** | | | | | | | | | |
|  |  |  |  |  |  |  |  |  |  |
| gi\|123913154 | Kunitz-type serine protease inhibitor 4; | 67.17 | 14 | (K)FcHLPVDSGIcR(A) | 49.59 | 1459.67 | 2 | 0 | Ca (C2, C11) |
|  |  |  |  |  |  |  |  |  |  |
| gi\|159883522 | trypsin inhibitor-3 precursor | 118.27 | 51 | (K)EFIYGGcHGNANNFPTR(D) | 71.97 | 1952.859 | 2 | 3.9 | Ca (C7) |
|  |  |  |  | (K)FcYLPADPGEcmAYIR(S) | 56.44 | 1977.842 | 2 | 1.9 | Ca (C2, C11); O (M12) |
|  |  |  |  | (R)SFYYDSESKK(C) | 54.25 | 1252.561 | 2 | 0.8 |  |
|  |  |  |  |  |  |  |  |  |  |
| gi\|239977245 | Kunitz-type serine protease inhibitor B1 | 69.75 | 31 | (K)FcYLPADPGEcLAHmR(S) | 30.99 | 1951.838 | 2 | 3 | Ca (C2, C10); O (M14) |
| **NGF (1 protein)** | | | | | | | | | |
| gi\|400499 | Venom nerve growth factor | 61.53 | 21 | (R)INTAcVcVISR(K) | 49.54 | 1291.6 | 2 | 0.6 | Ca (C5, C7) |
|  |  |  |  | (K)HWNSYcTTTDTFVR(A) | 23.96 | 1786.8 | 3 | 0.2 | Ca (C6) |

**Supplementary Table S3a.** Summary of different proteins identified in Indian Saw Scaled Viper (*E. c. carinatus*) venom by tandem mass spectrometry analysis of gel filtration peaks searched against *Echis* non-redundant protein database.

| **Sl No.** | **Accession** | **Description** | **Source organism** | **-10lgP** | **Coverage (%)** | **Distinct peptide** | **Avg. Mass (Da)** | **GF peaks** |
| --- | --- | --- | --- | --- | --- | --- | --- | --- |
| **Enzymatic proteins** | | | | | | | | |
| **P-I SVMP (1 protein)** | | | | | | | | |
| 1 | JAC96597.1 | Snake venom metalloproteinase N, partial | *Echis coloratus* | 146.85 | 25 | 2 | 18290 | 1,7,10 |
| **P-II SVMP (4 protein)** | | | | | | | | |
| 1 | ADI47726.1 | metalloproteinase | *Echis carinatus sochureki* | 113.05 | 19 | 1 | 12578 | 3,5,6-7,10 |
| 2 | ADI47717.1 | metalloproteinase | *Echis coloratus* | 122.87 | 17 | 1 | 13935 | 5-6,10 |
| 3 | ADI47722.1 | metalloproteinase | *Echis carinatus sochureki* | 107.64 | 4 | 1 | 53640 | 3,7-8,10 |
| 4 | ADI47689.1 | metalloproteinase | *Echis coloratus* | 72.44 | 4 | 1 | 53916 | 3,7-8 |
| **P-III SVMP (19 protein)** | | | | | | | | |
| 1 | ADI47598.1 | metalloproteinase, partial | *Echis carinatus sochureki* | 193.47 | 20 | 2 | 35963 | 1,4-8,10 |
| 2 | ADI47643.1 | metalloproteinase | *Echis coloratus* | 168.73 | 8 | 2 | 69905 | 1-2,7,10 |
| 3 | ADI47593.1 | metalloproteinase, | *Echis carinatus sochureki* | 145.26 | 14 | 3 | 28319 | 1,3-8 |
| 4 | ADI47664.1 | metalloproteinase, partial | *Echis pyramidum leakeyi* | 120.98 | 12 | 2 | 36939 | 1-3,7-8 |
| 5 | ADI47590.1 | metalloproteinase | *Echis carinatus sochureki* | 114.86 | 6 | 2 | 69526 | 1-2,4 |
| 6 | ADW54341.1 | group III snake venom metalloproteinase | *Echis ocellatus* | 95.55 | 3 | 1 | 69422 | 1,3 |
| 7 | ANT80539.1 | venom metalloprotease PIII-SVMP EOC00089 | *Echis ocellatus* | 36.06 | 2 | 1 | 68419 | 1 |
| 8 | ADI47635.1 | metalloproteinase | *Echis coloratus* | 106.54 | 5 | 1 | 70808 | 2,4,6,10 |
| 9 | ADI47645.1 | metalloproteinase | *Echis coloratus* | 77.67 | 3 | 1 | 69834 | 2 |
| 10 | ADW54348.1 | group III snake venom metalloproteinase | *Echis ocellatus* | 76.14 | 3 | 1 | 69412 | 1,3,10 |
| 11 | ADW54346.1 | group III snake venom metalloproteinase, partial | *Echis ocellatus* | 114.96 | 6 | 1 | 63254 | 1,3,7-8 |
| 12 | ADI47580.1 | metalloproteinase | *Echis carinatus sochureki* | 135.56 | 6 | 1 | 68205 | 1,3,7 |
| 13 | ADI47584.1 | metalloproteinase | *Echis carinatus sochureki* | 73.96 | 3 | 1 | 69371 | 1,3,7-8 |
| 14 | ADI47601.1 | metalloproteinase, partial | *Echis carinatus sochureki* | 139.81 | 12 | 4 | 41105 | 1,3,7-8,10 |
| 15 | ADI47604.1 | metalloproteinase, partial | *Echis carinatus sochureki* | 112.99 | 10 | 1 | 27821 | 1,3,6-8 |
| 16 | CAJ01683.1 | Group III snake venom metalloproteinase | *Echis ocellatus* | 99.59 | 4 | 1 | 69598 | 2-4,7-8 |
| 17 | ADI47583.1 | metalloproteinase | *Echis carinatus sochureki* | 169.87 | 11 | 2 | 46177 | 3,7-8,10 |
| 18 | ADI47592.1 | metalloproteinase | *Echis carinatus sochureki* | 127.79 | 6 | 1 | 68427 | 7,8 |
| 19 | ADI47609.1 | metalloproteinase, partial | *Echis coloratus* | 166.67 | 10 | 2 | 56407 | 3-4,7-8,10 |
| **PLA_2_ (2 proteins)** | | | | | | | | |
| 1 | 1OZ6 | Chain A, X-ray Structure Of Acidic Phospholipase A2 From Indian Saw- Scaled Viper (*Echis* Carinatus) With A Potent Platelet Aggregation Inhibitory Activity | *Echis carinatus* | 191.97 | 57 | 3 | 13711 | 3-4,7-10 |
| 2 | JAC96563.1 | Phospholipase A2 Group IIE, partial | *Echis coloratus* | 62.89 | 6 | 1 | 13353 | 7,8-10 |
| **SVSP (2 proteins)** | | | | | | | | |
| 1 | JAC96574.1 | Serine protease D | *Echis coloratus* | 106.51 | 8 | 2 | 28837 | 2,3-8 |
| 2 | ADI47567.1 | serine protease, partial | *Echis coloratus* | 61.5 | 5 | 1 | 25441 | 3,7 |
| 3 | ADC52861.1 | serine proteinase, partial | *Echis ocellatus* | 30.35 | 3 | 1 | 28286 | 6 |
| 4 | ADI47565.1 | serine protease, partial | *Echis carinatus sochureki* | 76.94 | 10 | 1 | 26218 | 7-8,10 |
| 5 | JAC96575.1 | Serine protease C, partial | *Echis coloratus* | 70.08 | 8 | 1 | 13344 | 7,8 |
| 6 | ADI47576.1 | serine protease, partial | *Echis coloratus* | 70.22 | 5 | 2 | 28184 | 2-3,7-8 |
| 7 | ADI47570.1 | serine protease, partial | *Echis coloratus* | 58.17 | 4 | 1 | 26457 | 3-6,9 |
| **LAAO (1 protein)** | | | | | | | | |
| 1 | CAQ72894.1 | L-amino oxidase | *Echis ocellatus* | 115.24 | 4 | 1 | 56523 | 1,2-10 |
| **PLB (1 protein)** | | | | | | | | |
| 1 | JAC96587.1 | Phospholipase B | *Echis coloratus* | 105.7 | 5 | 3 | 64541 | 1,2-5 |
| **ASPro** **(1 protein)** | | | | | | | | |
| 1 | CAJ55260.1 | renin-like aspartic protease | *Echis ocellatus* | 72.32 | 3 | 1 | 43872 | 3 |
| **Snaclec (6 proteins)** | | | | | | | | |
| 1 | Q6X5S4.1 | Snaclec 9 | *Echis carinatus sochureki* | 37.18 | 4 | 1 | 16733 | 1,3 |
| 2 | Q7T2Q1.1 | Full=Snaclec EMS16 subunit alpha | *Echis multisquamatus* | 142.69 | 34 | 4 | 18214 | 1,3-7,9 |
| 3 | JAC96620.1 | C-type lectin G, partial | *Echis coloratus* | 50.23 | 5 | 1 | 14817 | 4 |
| 4 | JAC96617.1 | C-type lectin J | *Echis coloratus* | 132.66 | 23 | 4 | 18279 | 1-8,10 |
| 5 | AAP41218.2 | echicetin A-chain | *Echis carinatus* | 119.78 | 31 | 2 | 15363 | 1,2-9 |
| 6 | P81996.1 | Snaclec echicetin subunit beta | *Echis carinatus sochureki* | 110.75 | 31 | 1 | 14869 | 3-7,9 |
| **CRISP (1 protein)** | | | | | | | | |
| 1 | P0DMT4.1 | Cysteine-rich venom protein | *Echis coloratus* | 146.54 | 14 | 2 | 24699 | 5,6-10 |
| **Disntegrin (3 proteins)** | | | | | | | | |
| 1 | ANJ00861.1 | jerdostatin-1 | *Echis ocellatus* | 65.4 | 9 | 1 | 11849 | 10 |
| 2 | P81631.1 | Disintegrin EC3B | *Echis carinatus* | 122.87 | 33 | 1 | 7424 | 5-6,10 |
| 3 | P0C6A3.1 | Disintegrin EMS11A | *Echis multisquamatus* | 113.05 | 32 | 1 | 7469 | 3,5-7,10 |
| **NGF (1 protein)** | | | | | | | | |
| 1 | JAC88974.1 | nerve growth factor a | *Echis coloratus* | 71.96 | 5 | 1 | 27186 | 3,7-8 |

**Supplementary Table S3b.** List of all the proteins identified by LC-MS/MS analysis of gel filtration fractions of ECV against *Echis* non-redundant protein database. The table shows the identified peptide ions, their mass, charge (z), score for the ID, ΔM (ppm), modified residues. Ca and O represent carbamidomethylation of cysteine and oxidation of methionine, respectively.

| **P-I SVMP (1 protein)** | | | | | | | | | | |
| --- | --- | --- | --- | --- | --- | --- | --- | --- | --- | --- |
|  |  |  |  |  |  |  |  |  |  |  |
| 1 | JAC96597.1 | Snake venom metalloproteinase N, partial | 146.85 | 25 | R.ATVAEDAcFQFNSLGSDYGYcR.K | 101.07 | 2530.053 | 2 | 3.7 | Ca (C8,C22) |
|  |  |  |  |  |  |  |  |  |  |  |
| **P-II SVMP (4 proteins)** | | | | | | | | | | |
| 1 | ADI47726.1 | metalloproteinase | 113.05 | 19 | K.FLNAGTIcKK.A | 48.6 | 1150.617 | 3 | 1.6 | Ca (C8) |
|  |  |  |  |  | R.EGEHcISGPccR.N | 42.32 | 1460.559 | 3 | 0.6 | Ca (C5, 10,C11) |
|  |  |  |  |  |  |  |  |  |  |  |
| 2 | ADI47717.1 | metalloproteinase | 122.87 | 17 | K.FLNAGTIcKR.A | 68.24 | 1178.623 | 2 | 0.7 | Ca (C8) |
|  |  |  |  |  |  |  |  |  |  |  |
| 3 | ADI47722.1 | metalloproteinase | 107.64 | 4 | K.RAVGDDmDDYcSGITPDcPR.N | 55.91 | 2314.925 | 3 | 0.6 | O (M7); Ca(C11,C18) |
|  |  |  |  |  |  |  |  |  |  |  |
| 4 | ADI47689.1 | metalloproteinase | 72.44 | 4 | K.GDWMDDYcTGISSDcPR.N | 72.44 | 2033.755 | 2 | -1.6 | Ca (C8,C15) |
| **P-III SVMP (19 proteins)** | | | | | | | | | | |
| 1 | ADI47598.1 | metalloproteinase, partial | 193.47 | 20 | R.KASDEcDVPEYcTGQSADcPR.N | 111.27 | 2443.968 | 2 | 2.4 | Ca (C6,C12,C19) |
|  |  |  |  |  | K.NQcISLFGSR.A | 65.95 | 1180.566 | 2 | 2.3 | Ca (C3) |
|  |  |  |  |  | K.cILDPPLR.K | 43.81 | 1110.622 | 2 | -0.7 | Ca (C1) |
|  |  |  |  |  | R.ATVAEDScFQENLK.G | 31.38 | 1610.725 | 2 | 1.1 | Ca (C8) |
|  |  |  |  |  |  |  |  |  |  |  |
| 2 | ADI47643.1 | metalloproteinase | 168.73 | 8 | K.ARHEcDLPEHcTGQSAEcPIDEFQR.N | 85.84 | 3041.282 | 3 | 2.5 | Ca (C5,C11,C18) |
|  |  |  |  |  | R.LYcLDNSPGNKNPcK.M | 73.57 | 1778.808 | 2 | 1.8 | Ca (C3,C14) |
|  |  |  |  |  |  |  |  |  |  |  |
| 3 | ADI47593.1 | metalloproteinase, | 145.26 | 14 | R.DDcDVPEHcTGQSAEcPR.N | 102.11 | 2131.799 | 2 | 2.6 | Ca (C3,C11,C18) |
|  |  |  |  |  | K.cIFDPPLR.N | 45.63 | 1016.511 | 2 | 1.2 | Ca (C1) |
|  |  |  |  |  | R.GIFDGYcTK.E | 42.82 | 1059.47 | 2 | 1.2 | Ca (C7) |
|  |  |  |  |  |  |  |  |  |  |  |
| 4 | ADI47664.1 | metalloproteinase, partial | 120.98 | 12 | R.KENDVPIPcAQEDVK.C | 64.8 | 1740.835 | 2 | 2.7 | Ca (C9) |
|  |  |  |  |  | R.YPYGDEGmVDPGTK.C | 58.72 | 1543.65 | 2 | 0.6 | O (M8) |
|  |  |  |  |  |  |  |  |  |  |  |
|  |  |  |  |  |  |  |  |  |  |  |
| 5 | ADI47590.1 | metalloproteinase | 114.86 | 6 | R.VPLVGMEFWNQR.D | 72.15 | 1474.739 | 2 | 3.9 |  |
|  |  |  |  |  | R.TDIVSPPVcGNALLEK.G | 52.02 | 1711.882 | 2 | 2.1 | Ca (C9) |
|  |  |  |  |  |  |  |  |  |  |  |
| 6 | ADW54341.1 | group III snake venom metalloproteinase | 95.55 | 3 | R.LGNTYAYcR.K | 59.32 | 1116.502 | 2 | 0.6 | Ca (C8) |
|  |  |  |  |  | K.cPLTLYQcR.A | 55.06 | 1209.564 | 2 | 2.5 | Ca (C1,C14) |
|  |  |  |  |  |  |  |  |  |  |  |
| 7 | ANT80539.1 | venom metalloprotease PIII-SVMP EOC00089 | 36.06 | 2 | R.KENDVEIPcAPEDVK.C | 36.06 | 1741.819 | 2 | 5 | Ca (C9) |
|  |  |  |  |  |  |  |  |  |  |  |
| 8 | ADI47635.1 | metalloproteinase | 106.54 | 5 | K.YENIEKEDEAPK.M | 67.79 | 1463.678 | 3 | -0.2 |  |
|  |  |  |  |  | R.VSDLLNR.K | 45.41 | 815.4501 | 2 | 1.4 |  |
|  |  |  |  |  |  |  |  |  |  |  |
| 9 | ADI47645.1 | metalloproteinase | 77.67 | 3 | R.HCVDVTRAY | 19.76 | 1062.492 | 2 | 8.1 |  |
|  |  |  |  |  |  |  |  |  |  |  |
| 10 | ADW54348.1 | group III snake venom metalloproteinase | 76.14 | 3 | R.LFcEIVK.N | 45.1 | 907.4837 | 2 | -0.2 | Ca (C3) |
|  |  |  |  |  |  |  |  |  |  |  |
| 11 | ADW54346.1 | group III snake venom metalloproteinase, partial | 114.96 | 6 | R.TDIISPPVcGNDLLEK.G | 62.62 | 1769.887 | 2 | 1 | Ca (C9) |
|  |  |  |  |  | R.YPccDAAScK.L | 52.29 | 1230.447 | 2 | 0.2 | Ca (C3,C4,C9) |
|  |  |  |  |  |  |  |  |  |  |  |
| 12 | ADI47580.1 | metalloproteinase | 135.56 | 6 | R.KENDVPVPcAQEDVK.C | 74.28 | 1726.82 | 2 | 1.4 | Ca (C9) |
|  |  |  |  |  | R.LFcETEPNmcR.H | 45.32 | 1471.589 | 2 | 0.1 | Ca (C3,C10) O (M9) |
|  |  |  |  |  |  |  |  |  |  |  |
| 13 | ADI47584.1 | metalloproteinase | 73.96 | 3 | R.LFcVEPSTGNK.I | 54.26 | 1250.597 | 2 | -1.2 | Ca (C3) |
|  |  |  |  |  | R.HcVDVNTAY | 39.41 | 1077.455 | 2 | -0.1 | Ca (C2) |
|  |  |  |  |  |  |  |  |  |  |  |
| 14 | ADI47601.1 | metalloproteinase, partial | 139.81 | 12 | R.SADcPTDYFHR.N | 74.16 | 1367.556 | 2 | 0.7 | Ca (C4) |
|  |  |  |  |  | K.LPIDNTPLcNYR.Y | 70.18 | 1474.724 | 2 | 0.1 | Ca (C9) |
|  |  |  |  |  | R.FATAETVcRPAK.S | 56.04 | 1349.676 | 2 | 0.8 | Ca (C8) |
|  |  |  |  |  | R.NREcVNVNTAY | 47.45 | 1338.599 | 2 | 0.1 | Ca (C4) |
|  |  |  |  |  |  |  |  |  |  |  |
| 15 | ADI47604.1 | metalloproteinase, partial | 112.99 | 10 | R.TDIVSPPVcGNDLLER.G | 65.53 | 1783.877 | 2 | 2 | Ca (C9) |
|  |  |  |  |  |  |  |  |  |  |  |
| 16 | CAJ01683.1 | Group III snake venom metalloproteinase | 99.59 | 4 | R.TDIVSPPVcGNDLLEK.G | 71.27 | 1755.871 | 2 | 0.3 | Ca (C9) |
|  |  |  |  |  |  |  |  |  |  |  |
| 17 | ADI47583.1 | metalloproteinase | 169.87 | 11 | R.NGQPcLNNQGYcYNGK.C | 74.3 | 1885.784 | 2 | 1.9 | Ca (C5,C12) |
|  |  |  |  |  | K.KAESYFYcR.K | 66.34 | 1222.544 | 2 | 0.1 | Ca (C8) |
|  |  |  |  |  | R.LFcEIIK.D | 60.95 | 921.4993 | 2 | 0.1 | Ca (C3) |
|  |  |  |  |  | R.KENDVmIPcAPEDVK.C | 55.64 | 1759.812 | 2 | -0.1 | O (M6), Ca (C9) |
|  |  |  |  |  |  |  |  |  |  |  |
| 18 | ADI47592.1 | metalloproteinase | 127.79 | 6 | R.LFcDIGPNR.C | 75.89 | 1090.523 | 2 | -1 | Ca (C3) |
|  |  |  |  |  | R.NGQPCLHNYGYcYNGK.C | 42 | 1886.783 | 2 | -4 | Ca (C12) |
|  |  |  |  |  |  |  |  |  |  |  |
| 19 | ADI47609.1 | metalloproteinase, partial | 166.67 | 10 | R.SEcDLPEYcTGQSADcPTDHFHR.N | 80.58 | 2781.085 | 2 | 1.4 | Ca (C3,C9,C16) |
|  |  |  |  |  | R.HcVDVTTAY | 56.64 | 1064.46 | 2 | -0.5 | Ca (C2) |
| **PLA_2_ (2 proteins)** | | | | | | | | | | |
| 1 | 1OZ6 | Chain A, X-ray Structure Of Acidic Phospholipase A2 From Indian Saw- Scaled Viper (*Echis* Carinatus) With A Potent Platelet Aggregation Inhibitory Activity | 191.97 | 57 | K.LPILSYGSYGcYcGWGGQGPPK.D | 89.92 | 2416.098 | 2 | -0.2 | Ca (C11,C13) |
|  |  |  |  |  | R.AVcEcDREAAIcLGENVNTYDKK.Y | 71.63 | 2714.21 | 3 | 0.9 | Ca (C3,C5,C12) |
|  |  |  |  |  | NLYQFGR.M | 55.51 | 896.4504 | 2 | 0.4 |  |
|  |  |  |  |  | R.FENGDIIcDNKDPcKR.A | 49.8 | 1979.883 | 3 | 0.9 | Ca (C8,C14) |
|  |  |  |  |  |  | 46.46 | 1823.782 | 2 | 1 | Ca (C8,C14) |
|  |  |  |  |  |  |  |  |  |  |  |
| 2 | JAC96563.1 | Phospholipase A2 Group IIE, partial | 62.89 | 6 | K.AAALcFR.H | 62.89 | 807.4061 | 2 | 0.6 | Ca (C5) |
|  |  |  |  |  |  |  |  |  |  |  |
| **SVSP (7 proteins)** | | | | | | | | | | |
| 1 | JAC96574.1 | Serine protease D | 106.51 | 8 | R.TFcAGVLQGGK.D | 80.99 | 1136.565 | 2 | -0.1 | Ca (C3) |
|  |  |  |  |  | R.AAYPWLPVNSR.T | 51.02 | 1272.662 | 2 | 0.5 |  |
|  |  |  |  |  |  |  |  |  |  |  |
| 2 | ADI47567.1 | serine protease, partial | 61.5 | 5 | R.TLcAGVLEGGK.D | 61.5 | 1103.565 | 2 | -0.4 | Ca (C3) |
|  |  |  |  |  |  |  |  |  |  |  |
| 3 | ADC52861.1 | serine proteinase, partial | 30.35 | 3 | R.TLCAGILGR.R | 30.35 | 902.5007 | 2 | 1.7 |  |
|  |  |  |  |  |  |  |  |  |  |  |
| 4 | ADI47565.1 | serine protease, partial | 76.94 | 10 | R.TLcAGVLEGGIDScK.A | 33.56 | 1578.738 | 2 | 1 | Ca (C3,C14) |
|  |  |  |  |  |  |  |  |  |  |  |
| 5 | JAC96575.1 | Serine protease C, partial | 70.08 | 8 | R.ETYFcLPSR.N | 61.18 | 1171.533 | 2 | -0.8 | Ca (C5) |
|  |  |  |  |  |  |  |  |  |  |  |
| 6 | ADI47576.1 | serine protease, partial | 70.22 | 5 | R.TLcAGILR.G | 54.34 | 902.5007 | 2 | -0.6 | Ca (C3) |
|  |  |  |  |  | K.YFcLPR.R | 31.74 | 854.4109 | 2 | -0.1 | Ca (C3) |
|  |  |  |  |  |  |  |  |  |  |  |
| 7 | ADI47570.1 | serine protease, partial | 58.17 | 4 | R.EAYGGLPEK.S | 58.17 | 962.4709 | 2 | 0.2 |  |
| **LAAO (1 protein)** | |  |  |  |  |  |  |  |  |  |
| 1 | CAQ72894.1 | L-amino oxidase | 115.24 | 4 | K.KFWEDDGIQGGK.S | 60.14 | 1378.652 | 2 | 1.3 |  |
|  |  |  |  |  |  |  |  |  |  |  |
| **PLB (1 protein)** | | | | | | | | | | |
| 1 | JAC96587.1 | Phospholipase B | 105.7 | 5 | K.YSDQTEVLR.H | 69.06 | 1109.535 | 2 | 1.8 |  |
|  |  |  |  |  | K.TWAQIFEK.E | 55.52 | 1021.523 | 2 | 2.2 |  |
|  |  |  |  |  | K.SDFLSMPDVSR.I | 26.62 | 1252.576 | 2 | 4.3 |  |
|  |  |  |  |  |  |  |  |  |  |  |
| **ASPro (1 protein)** | | | | | | | | | | |
| 1 | CAJ55260.1 | renin-like aspartic protease | 72.32 | 3 | R.YDSSESSTYKPK.G | 60.09 | 1390.625 | 2 | 0.8 |  |
| **Snaclec (6 proteins)** | | | | | | | | | |  |
|  |  |  |  |  |  |  |  |  |  |  |
| 1 | Q6X5S4.1 | Snaclec 9 | 37.18 | 4 | R.VFDEPK.T | 37.18 | 733.3646 | 2 | -0.2 |  |
|  |  |  |  |  |  |  |  |  |  |  |
| 2 | Q7T2Q1.1 | Full=Snaclec EMS16 subunit alpha | 142.69 | 34 | R.REEQQcNPEWNDGSK.I | 71.31 | 1875.781 | 2 | 0.6 | Ca (C6) |
|  |  |  |  |  | K.DGHLVSIQSR.E | 52.54 | 1110.578 | 2 | 0 |  |
|  |  |  |  |  | K.IIYVNWK.E | 46.72 | 934.5276 | 2 | -0.5 |  |
|  |  |  |  |  | K.WTNFHDWNNINcEDLYPFVcK.F | 45.88 | 2771.19 | 3 | 0.6 | Ca (C12,C20) |
|  |  |  |  |  |  |  |  |  |  |  |
| 3 | JAC96620.1 | C-type lectin G, partial | 50.23 | 5 | K.VFNEYK.S | 50.23 | 798.3912 | 2 | 0.6 |  |
|  |  |  |  |  |  |  |  |  |  |  |
| 4 | JAC96617.1 | C-type lectin J | 132.66 | 23 | K.SSADYVWIGLWNK.R | 81.87 | 1537.757 | 2 | 2.7 |  |
|  |  |  |  |  | R.cGDDYPFVcK.F | 57.29 | 1259.495 | 2 | 0.5 | Ca (C1,C9) |
|  |  |  |  |  | R.TWFNLR.C | 40.26 | 835.4341 | 2 | -0.4 |  |
|  |  |  |  |  | K.NcFGLEK.K | 34.89 | 866.3956 | 2 | 0.6 | Ca (C2) |
|  |  |  |  |  |  |  |  |  |  |  |
| 5 | AAP41218.2 | echicetin A-chain | 119.78 | 31 | R.SYEIAIR.Y | 57.43 | 850.4548 | 2 | 0.6 |  |
|  |  |  |  |  | K.IWTGLSER.S | 48 | 960.5029 | 2 | -1.7 |  |
|  |  |  |  |  |  |  |  |  |  |  |
| 6 | P81996.1 | Snaclec echicetin subunit beta | 110.75 | 31 | R.DcYWEWSDGAQLDYK.A | 68.77 | 1934.778 | 2 | 2.8 | Ca (C2) |
|  |  |  |  |  | NcLPDWSVYEGYcYK.V | 50.56 | 1952.807 | 2 | 1.4 | Ca (C2) |
| **CRISP (1 protein)** | | | | | | | | | | |
|  |  |  |  |  |  |  |  |  |  |  |
| 1 | P0DMT4.1 | Cysteine-rich venom protein | 146.54 | 14 | R.KPEIQNEIIDLHNSLR.R | 98.92 | 1918.027 | 3 | -0.1 |  |
|  |  |  |  |  | K.SNcAAScFcHSEIK | 69.34 | 1669.665 | 2 | 0.4 | Ca (C3,C7,C9) |
|  |  |  |  |  |  |  |  |  |  |  |
| **Disintegrin (3 proteins)** | | | | | | | | | | |
| 1 | ANJ00861.1 | jerdostatin-1 | 65.4 | 9 | K.LKPAGTTcWR.T | 65.4 | 1188.607 | 2 | 1.1 | Ca (C8) |
|  |  |  |  |  |  |  |  |  |  |  |
| 2 | P81631.1 | Disintegrin EC3B | 122.87 | 33 | K.FLNAGTIcKR.A | 68.24 | 1178.623 | 2 | 0.7 | Ca (C8) |
|  |  |  |  |  |  |  |  |  |  |  |
| 3 | P0C6A3.1 | Disintegrin EMS11A | 113.05 | 32 | K.FLNAGTIcKK.A | 48.6 | 1150.617 | 3 | 1.6 | Ca (C8) |
| **NGF (1 protein)** | | | | | | | | | | |
| 1 | JAC88974.1 | nerve growth factor a | 71.96 | 5 | R.INTAcVcVISR.K | 50.88 | 1291.638 | 2 | 0.6 | Ca (C5,C7) |

**Supplementary Table S4a.** Summary of different proteins identified in ECV by tandem mass spectrometry analysis of gel filtration peaks searched against *Echis carinatus* non-redundant protein database.

| **Sl No** | **Accession** | **Description** | **Source organism** | **-10lgP** | **Coverage (%)** | **Distinct peptides** | **Avg. Mass (Da)** | **GF peak(s)** |
| --- | --- | --- | --- | --- | --- | --- | --- | --- |
| **Enzymatic proteins** | | | | | | | | |
| **SVMP (12 proteins)** | | | | | | | | |
| 1 | ADI47593.1 | metalloproteinase, partial | *Echis carinatus sochureki* | 131.25 | 14 | 4 | 28319 | 1-8 |
| 2 | ADI47590.1 | metalloproteinase | *Echis carinatus sochureki* | 115.54 | 6 | 2 | 69526 | 1,2,8 |
| 3 | ADI47597.1 | metalloproteinase, partial | *Echis carinatus sochureki* | 70.36 | 3 | 1 | 42715 | 1,3,7,8 |
| 4 | ADI47581.1 | metalloproteinase | *Echis carinatus sochureki* | 220.67 | 12 | 5 | 68765 | 1-8, 10 |
| 5 | ADI47578.1 | metalloproteinase, partial | *Echis carinatus sochureki* | 123.42 | 10 | 6 | 63999 | 1-4,6-8,10 |
| 6 | ADI47580.1 | metalloproteinase | *Echis carinatus sochureki* | 117.08 | 6 | 2 | 68205 | 1,3,7 |
| 7 | ADI47604.1 | metalloproteinase, partial | *Echis carinatus sochureki* | 109.86 | 19 | 2 | 27821 | 1,3,6,7 |
| 8 | ADI47585.1 | metalloproteinase | *Echis carinatus sochureki* | 62.92 | 3 | 1 | 69245 | 1,3,7 |
| 9 | ADI47725.1 | metalloproteinase, partial | *Echis carinatus sochureki* | 178.71 | 11 | 3 | 27704 | 2-10 |
| 10 | ADI47588.1 | metalloproteinase, partial | *Echis carinatus sochureki* | 128.63 | 7 | 4 | 57650 | 1,3,7,8,10 |
| 11 | ADI47582.1 | metalloproteinase, partial | *Echis carinatus sochureki* | 136.77 | 14 | 4 | 39004 | 3,7,8,10 |
| 12 | ADI47589.1 | metalloproteinase | *Echis carinatus sochureki* | 107.76 | 6 | 1 | 68453 | 7,8 |
| **SVSP (1 protein)** | | | | | | | | |
| 1 | ADI47565.1 | serine protease, partial | *Echis carinatus sochureki* | 67.06 | 10 | 2 | 26218 | 7,8,10 |
| **PLA_2_ (1 protein)** | | | | | | | | |
| 1 | 1OZ6 | Chain A, X-ray Structure Of Acidic Phospholipase A2 From Indian Saw- Scaled Viper With A Potent Platelet Aggregation Inhibitory Activity | *Echis carinatus* | 204.59 | 57 | 5 | 13711 | 3,4,7-10 |
| **Non-enzymatic proteins** | | | | | | | | |
| **Snaclec (5 proteins)** | | | | | | | | |
| 1 | AAP41218.2 | echicetin A-chain | *Echis carinatus* | 119.54 | 31 | 4 | 15363 | 1-9 |
| 2 | P81017.1 | Snaclec echicetin subunit alpha | *Echis carinatus sochureki* | 40.03 | 7 | 1 | 15803 | 3 |
| 3 | Q6X5S4.1 | Snaclec 9 | *Echis carinatus sochureki* | 34.37 | 4 | 1 | 16733 | 3 |
| 4 | Q9PSM9.1 | Snaclec coagulation factor IX/factor X-binding protein subunit A | *Echis carinatus* | 37.26 | 5 | 1 | 15439 | 4 |
| 5 | P81996.1 | Snaclec echicetin subunit beta | *Echis carinatus sochureki* | 116.3 | 31 | 1 | 14869 | 3-7,9 |
| **CRISP (1 protein)** | | | | | | | | |
| 1 | P0DMT4.1 | Cysteine-rich venom protein | *Echis carinatus* | 139.45 | 19 | 3 | 24699 | 3-10 |
| **Disintegrin (2 proteins)** | | | | | | | | |
| 1 | Q5EE07.1 | Disintegrin | *Echis carinatus* | 173.93 | 88 | 3 | 7127 | 3,5-8,10 |
| 2 | P82465.2 | Disintegrin EC6 subunit alpha | *Echis carinatus sochureki* | 99.79 | 19 | 1 | 12578 | 3,5-7,10 |

**Supplementary table S4b.** List of all the proteins identified by LC-MS/MS analysis of gel filtration fractions of ECV against *Echis carinatus* database. The table shows the identified peptide ions, their mass, charge (z), score for the ID, ΔM (ppm), modified residues. Ca and O represent carbamidomethylation of cysteine and oxidation of methionine, respectively.

| **Accession** | **Description** | **-10lgP** | **Coverage (%)** | **Peptide** | **-10lgP** | **Mass (Da)** | **z** | **ppm** | **Modified residues** |
| --- | --- | --- | --- | --- | --- | --- | --- | --- | --- |
| **Enzymatic proteins** | | | | | | | | | |
| **SVMP (12 proteins)** | | | | | | | | | |
| ADI47593.1 | metalloproteinase, partial | 131.25 | 14 | R.DDcDVPEHcTGQSAEcPR.N | 82.45 | 2131.80 | 2 | 2.6 | Ca (C3, C9, C16) |
|  |  |  |  | R.GIFDGYcTK.E | 48.17 | 1059.47 | 2 | 1.2 | Ca (C7) |
|  |  |  |  | K.cIFDPPLR.N | 44.94 | 1016.51 | 2 | 1.2 | Ca (C1) |
|  |  |  |  | R.RFPcAPQDVK.C | 40.66 | 1216.60 | 2 | -0.2 | Ca (C4) |
|  |  |  |  |  |  |  |  |  |  |
| ADI47590.1 | metalloproteinase | 115.54 | 6 | R.VPLVGMEFWNQR.D | 75.67 | 1474.74 | 2 | 3.9 |  |
|  |  |  |  | R.TDIVSPPVcGNALLEK.G | 50.52 | 1711.88 | 2 | 2.1 | Ca (C9) |
|  |  |  |  |  |  |  |  |  |  |
| ADI47597.1 | metalloproteinase, partial | 70.36 | 3 | R.LFcVEPSTGNK.I | 61.15 | 1250.60 | 2 | 0.9 | Ca (C3) |
|  |  |  |  |  |  |  |  |  |  |
| ADI47581.1 | metalloproteinase | 220.67 | 12 | R.KASDEcDVPEYcTGQSADcPR.N | 120.03 | 2443.97 | 2 | 2.4 | Ca (C6, C12, C19) |
|  |  |  |  | R.ATVAEDScFQENLK.G | 81.52 | 1610.72 | 2 | 1.3 | Ca (C8) |
|  |  |  |  | K.NQcISLFGSR.A | 66.31 | 1180.57 | 2 | 0.8 | Ca (C3) |
|  |  |  |  | R.LYcLDNSSR.K | 57.02 | 1126.51 | 2 | 2.4 | Ca (C3) |
|  |  |  |  | R.VSDLLNR.K | 46.26 | 815.45 | 2 | 0.2 |  |
|  |  |  |  | R.KIPcAPQDVK.C | 41.50 | 1154.61 | 2 | 0.1 | Ca (C4) |
|  |  |  |  | K.cILDPPLRK.D | 41.10 | 1110.62 | 2 | -0.7 | Ca (C1) |
|  |  |  |  |  |  |  |  |  |  |
| ADI47578.1 | metalloproteinase, partial | 123.42 | 10 | K.IPcAPEDVK.C | 57.78 | 1027.50 | 2 | -1.8 | Ca (C3) |
|  |  |  |  | R.LYcFDNLPEHK.N | 54.66 | 1434.66 | 2 | 0.6 | Ca (C3) |
|  |  |  |  | R.LGSDYGYcR.K | 48.64 | 1089.46 | 2 | -1.1 | Ca (C8) |
|  |  |  |  | R.KcVDVNTAY- | 41.32 | 1068.49 | 2 | -0.1 | Ca (C2) |
|  |  |  |  | R.LSDLLNR.R | 34.83 | 829.47 | 2 | -0.2 |  |
|  |  |  |  | R.NGHPcQNNNGYcYNGK.C | 31.66 | 1895.74 | 3 | 0.6 | Ca (C5, C12) |
|  |  |  |  | R.NLFTIR.T | 29.43 | 762.44 | 2 | -0.7 |  |
|  |  |  |  |  |  |  |  |  |  |
| ADI47580.1 | metalloproteinase | 117.08 | 6 | R.KENDVPVPcAQEDVK.C | 65.15 | 1726.82 | 2 | 1.4 | Ca (C9) |
|  |  |  |  | R.LFcETEPNmcR.H | 46.82 | 1471.59 | 2 | 0.1 | Ca (C3, C10); O (M9) |
|  |  |  |  | R.YPccDAAScK.L | 44.89 | 1230.45 | 2 | 0.2 | Ca (C3, C4, C9) |
|  |  |  |  |  |  |  |  |  |  |
| ADI47604.1 | metalloproteinase, partial | 109.86 | 19 | R.TDIVSPPVcGNDLLER.G | 64.51 | 1783.88 | 2 | 2 | Ca (C10) |
|  |  |  |  | R.NEcDLPEYcTGQSGDcPTDHFHR.N | 29.22 | 2794.08 | 4 | 9.3 | Ca (C3, C9, C16) |
|  |  |  |  |  |  |  |  |  |  |
| ADI47585.1 | metalloproteinase | 62.92 | 3 | R.HcVDVTTAY- | 45.93 | 1064.46 | 2 | -0.4 | Ca (C2) |
|  |  |  |  | R.LFcEIVK.N | 33.99 | 907.48 | 2 | -0.2 | Ca (C3) |
|  |  |  |  |  |  |  |  |  |  |
| ADI47588.1 | metalloproteinase, partial | 128.63 | 7 | R.FATAETVcRPAK.S | 69.58 | 1349.68 | 2 | 1.4 | Ca (C8) |
|  |  |  |  | R.SADcPTDYFHR.N | 65.41 | 1367.56 | 2 | 1.3 | Ca (C4) |
|  |  |  |  | K.LPIDNTPLcNYR.Y | 67.92 | 1474.72 | 2 | 0.1 | Ca (C9) |
|  |  |  |  | R.NREcVNVNTAY- | 45.88 | 1338.60 | 2 | 0.1 | Ca (C4) |
|  |  |  |  |  |  |  |  |  |  |
| ADI47582.1 | metalloproteinase, partial | 136.77 | 14 | R.NGQPcLNNQGYcYNGK.C | 63.16 | 1885.78 | 2 | 1.9 | Ca (C5, C12) |
|  |  |  |  | K.KAESYFYcR.K | 51.04 | 1222.54 | 2 | 0.1 | Ca (C8) |
|  |  |  |  | R.LFcEIIK.D | 44.85 | 921.50 | 2 | 0.1 | Ca (C3) |
|  |  |  |  | R.KENDVmIPcAPEDVK.C | 43.14 | 1759.81 | 2 | -0.1 | Ca (C9); O (M6) |
|  |  |  |  |  |  |  |  |  |  |
| ADI47589.1 | metalloproteinase | 107.76 | 6 | R.LFcDIGPNR.C | 62.04 | 1090.52 | 2 | -1 | Ca (C3) |
|  |  |  |  | R.NGQPCLHNYGYcYNGK.C | 39.32 | 1886.78 | 2 | -4 | Ca (C12) |
|  |  |  |  |  |  |  |  |  |  |
| ADI47725.1 | metalloproteinase, partial | 178.71 | 11 | K.RDLINVVSSSIDTLR.S | 84.01 | 1686.93 | 2 | 2.1 |  |
|  |  |  |  | R.ERDLLNR.R | 30.66 | 914.49 | 2 | 2.2 |  |
|  |  |  |  | R.SFAEWRER.D | 22.39 | 1079.51 | 2 | 0.3 |  |
|  |  |  |  |  |  |  |  |  |  |
| **SVSP (1 protein)** | | | | | | | | | |
| ADI47565.1 | serine protease, partial | 67.06 | 10 | K.IFDYSVcR.G | 51.78 | 1058.49 | 2 | 0.9 | Ca (C7) |
|  |  |  |  | R.TLcAGVLEGGIDScK.A | 30.55 | 1578.74 | 2 | 1 | Ca (C3, C14) |
|  |  |  |  |  |  |  |  |  |  |
| **PLA_2_ (1 protein)** | | | | | | | | | |
| 1OZ6 | Chain A, X-ray Structure Of Acidic Phospholipase A2 From Indian Saw- Scaled Viper With A Potent Platelet Aggregation Inhibitory Activity | 204.59 | 57 | K.LPILSYGSYGcYcGWGGQGPPK.D | 92.47 | 2416.10 | 2 | -0.2 | Ca (C11, C13) |
|  |  |  |  | R.EAAIcLGENVNTYDKK.Y | 87.03 | 1823.87 | 2 | 1.6 | Ca (C5) |
|  |  |  |  | R.AVcEcDREAAIcLGENVNTYDKK.Y | 74.37 | 2714.21 | 3 | 0.9 | Ca (C3, C5, C12) |
|  |  |  |  | R.FENGDIIcDNKDPcKR.A | 54 | 1979.88 | 3 | 0.9 | Ca (C8, C14) |
|  |  |  |  | -NLYQFGR.M | 51.12 | 896.45 | 2 | 0.4 |  |
|  |  |  |  |  |  |  |  |  |  |
| **Non-enzymatic proteins** | | | | | | | | | |
| **Snaclec (5 proteins)** | | | | | | | | | |
| AAP41218.2 | echicetin A-chain | 119.54 | 31 | R.YSEcFVLEK.Q | 59.19 | 1173.54 | 2 | 0.4 | Ca (C4) |
|  |  |  |  | K.TWDEAEKFcNK.Q | 56.58 | 1426.62 | 2 | 2 | Ca (C9) |
|  |  |  |  | R.SYEIAIR.Y | 45.72 | 850.45 | 2 | 0.2 |  |
|  |  |  |  | K.IWTGLSER.S | 42.3 | 960.50 | 2 | -0.7 |  |
|  |  |  |  | K.YPVPR- | 31.21 | 630.35 | 2 | 0.2 |  |
|  |  |  |  |  |  |  |  |  |  |
| P81017.1 | Snaclec echicetin subunit alpha | 40.03 | 7 | R.LKTWDEAEK.Y | 40.03 | 1118.56 | 3 | -0.8 |  |
|  |  |  |  |  |  |  |  |  |  |
| Q6X5S4.1 | Snaclec 9 | 34.37 | 4 | R.VFDEPK.T | 34.37 | 733.36 | 2 | -0.2 |  |
|  |  |  |  |  |  |  |  |  |  |
| Q9PSM9.1 | Snaclec coagulation factor IX/factor X-binding protein subunit A | 37.26 | 5 | K.VFNEYK.T | 37.26 | 798.39 | 2 | 0.6 |  |
|  |  |  |  |  |  |  |  |  |  |
| P81996.1 | Snaclec echicetin subunit beta | 116.3 | 31 | R.DcYWEWSDGAQLDYK.A | 73.27 | 1934.78 | 2 | 2.8 | Ca (C2) |
|  |  |  |  | -NcLPDWSVYEGYcYK.V | 47.77 | 1952.81 | 2 | 1.4 | Ca (C2, C13) |
|  |  |  |  | K.DGHLVSFR.N | 60.09 | 929.47 | 2 | 1.5 |  |
|  |  |  |  |  |  |  |  |  |  |
| **CRISP (1 protein)** | | | | | | | | | |
| P0DMT4.1 | Cysteine-rich venom protein | 139.45 | 19 | -NVDFDSESPR.K | 56.58 | 1164.50 | 2 | 0.4 |  |
|  |  |  |  | K.SNcAAScFcHSEIK- | 53.57 | 1669.66 | 2 | -0.1 | Ca (C3, C7, C9) |
|  |  |  |  | R.KPEIQNEIIDLHNSLRR.S | 40.47 | 2074.13 | 4 | -0.1 |  |
|  |  |  |  |  |  |  |  |  |  |
| **Disintegrin (2 proteins)** | | | | | | | | | |
| Q5EE07.1 | Disintegrin | 173.93 | 88 | -NSVHPccDPVKcEPR.E | 56.19 | 1853.80 | 3 | 0.9 | Ca (C6, C7, C12) |
|  |  |  |  | R.RAmLDGLHDYcTGVTSDcPR.N | 53.53 | 2339.01 | 3 | 1.5 | Ca (C11, C18); O (M3) |
|  |  |  |  | K.FLNAGTIcKR.A | 52.74 | 1178.62 | 2 | 0.8 | Ca (C8) |
|  |  |  |  | R.EGEHcISGPccR.N | 46.40 | 1460.56 | 3 | 0.2 | Ca (C5, C10, C11) |
|  |  |  |  |  |  |  |  |  |  |
| P82465.2 | Disintegrin EC6 subunit alpha | 99.79 | 19 | K.FLNAGTIcKK.A | 40.64 | 1150.62 | 3 | 1.6 | Ca (C8) |

**Supplementary Table S5a.** Summary of different proteins identified in Indian Saw Scaled Viper (*E. c. carinatus*) venom by tandem mass spectrometry analysis of gel filtration peaks searched against Transcriptome Shotgun Assembly (TSA) sequences of *Echis coloratus*.

| **Sl. No.** | **Accession** | **Description** | **Source Organism** | **-10lgP** | **Coverage (%) Sample 1** | **Distinct peptides** | **Avg. Mass (Da)** | **GF peaks** |
| --- | --- | --- | --- | --- | --- | --- | --- | --- |
| **Enzymatic proteins** | | | | | | | | |
| **SVMP (6 proteins)** | | | | | | | | |
| 1 | JAC96604.1 | Snake venom metalloproteinase G, partial | *Echis coloratus* | 278.2 | 11 | 5 | 68160 | 1-3,5-7,10 |
| 2 | JAC96600.1 | Snake venom metalloproteinase K, partial | *Echis coloratus* | 213.31 | 9 | 2 | 48851 | 3,4-7 |
| 3 | JAC96597.1 | Snake venom metalloproteinase N, partial | *Echis coloratus* | 242.92 | 31 | 4 | 18290 | 1,3,5,7-8,10 |
| 4 | JAC96593.1 | Snake venom metalloproteinase R, partial | *Echis coloratus* | 97.44 | 7 | 1 | 15316 | 3 |
| 5 | JAC96602.1 | Snake venom metalloproteinase I, partial | *Echis coloratus* | 70.35 | 2 | 1 | 35174 | 4,5-6 |
| 6 | JAC96608.1 | Snake venom metalloproteinase C, partial | *Echis coloratus* | 175.26 | 12 | 2 | 16968 | 1,3-4,7-8 |
| **PLA_2_ (1 protein)** | | | | | | | | |
| 1 | JAC96563.1 | Phospholipase A2 Group IIE, partial | *Echis coloratus* | 106.87 | 6 | 1 | 13353 | 7,8-10 |
| **SVSP (2 proteins)** | | | | | | | | |
| 1 | JAC96577.1 | Serine protease A, partial | *Echis coloratus* | 50.62 | 6 | 1 | 10640 | 3 |
| 2 | JAC96575.1 | Serine protease C, partial | *Echis coloratus* | 105.85 | 8 | 1 | 13344 | 3,7-8 |
| **LAAO (1 protein)** | | | | | | | | |
| 1 | JAC96580.1 | L-amino acid oxidase B variant 1 | *Echis coloratus* | 244.33 | 7 | 4 | 56738 | 2,3-10 |
| **Non-enzymatic proteins** | | | | | | | | |
| **Snaclec (1 protein)** | | | | | | | | |
| 1 | JAC96617.1 | C-type lectin J | *Echis coloratus* | 268.08 | 23 | 4 | 18279 | 1-8,10 |
| **CRISP (1 protein)** | | | | | | | | |
| 1 | JAC96631.1 | Cysteine-Rich Secretory Protein B | *Echis coloratus* | 259.96 | 17 | 3 | 26686 | 5,6-10 |
| **NGF (1 protein)** | | | | | | | | |
| 1 | JAC88974.1 | nerve growth factor a | *Echis coloratus* | 112.4 | 10 | 2 | 27186 | 3,4-10 |
| **VEGF (1 protein)** | | | | | | | | |
| 1 | JAC96562.1 | Vascular endothelial growth factor F | *Echis coloratus* | 46.08 | 7 | 1 | 16163 | 4 |

**Supplementary Table S5b.** List of all the proteins identified by LC-MS/MS analysis of gel filtration fractions of ECV against the Transcriptome Shotgun Assembly (TSA) sequences of *Echis coloratus*. The table shows the identified peptide ions, their mass, charge (z), score for the ID, ΔM (ppm), modified residues. Ca and O represent carbamidomethylation of cysteine and oxidation of methionine, respectively.

| **Sl. No.** | **Accession** | **Description** | **-10lgP** | **Coverage (%)** | | **Peptide** | **-10lgP** | **Mass (Da)** | **z** | **ppm** | **PTM** |
| --- | --- | --- | --- | --- | --- | --- | --- | --- | --- | --- | --- |
| **Enzymatic proteins** | | | | | | | | | | | |
| **SVMP (6 proteins)** | | | | | | | | | | | |
|  |  |  |  |  |  | |  |  |  |  |  |
| 1 | JAC96604.1 | Snake venom metalloproteinase G, partial | 278.2 | 11 | R.ATVAEDScFQENLK.G | | 123.26 | 1610.725 | 2 | 1.3 | Ca(C8) |
|  |  |  |  |  | T.NQcISLFGSR.A | | 86.79 | 1180.566 | 2 | 0.8 | Ca(C3) |
|  |  |  |  |  | R.LYcLDNSSR.K | | 85.17 | 1126.508 | 2 | 2.4 | Ca(C3) |
|  |  |  |  |  | R.VSDLLNR.K | | 77.29 | 815.4501 | 2 | 0.2 |  |
|  |  |  |  |  | K.IPcAPQDVK.C | | 73.81 | 1154.612 | 2 | 0.1 | Ca(C3) |
|  |  |  |  |  | K.cILDPPLRK.D | | 43.08 | 1110.622 | 3 | 0.1 | Ca(C1) |
|  |  |  |  |  |  | |  |  |  |  |  |
| 2 | JAC96600.1 | Snake venom metalloproteinase K, partial | 213.31 | 9 | P.LKTDIVSPPVcGNYFVEVGEEcDcGSPR.N | | 68.73 | 3183.431 | 3 | 0.1 | Ca(C11,C22,C24) |
|  |  |  |  |  | N.KNIPcAPQDVK.C | | 46.18 | 1268.655 | 2 | 0.1 | Ca(C5) |
|  |  |  |  |  |  | |  |  |  |  |  |
| 3 | JAC96597.1 | Snake venom metalloproteinase N, partial | 242.92 | 31 | R.ATVAEDAcFQFNSLGSDYGYcR.K | | 122.8 | 2530.053 | 2 | 3.4 | Ca(C8) |
|  |  |  |  |  | R.LYcFDNLPEHK.N | | 89.73 | 1434.66 | 2 | 0.6 | Ca(C3) |
|  |  |  |  |  | K.IPcAPEDVK.C | | 86.93 | 1027.501 | 2 | -1.8 | Ca(C3) |
|  |  |  |  |  | N.GKcVDVNTAY | | 52.23 | 1125.513 | 2 | -0.3 | Ca(C3) |
|  |  |  |  |  |  | |  |  |  |  |  |
| 4 | JAC96593.1 | Snake venom metalloproteinase R, partial | 97.44 | 7 | R.HcVDVNTAY | | 64.88 | 1077.455 | 2 | -0.1 | Ca(C2) |
|  |  |  |  |  |  | |  |  |  |  |  |
| 5 | JAC96602.1 | Snake venom metalloproteinase I, partial | 70.35 | 2 | R.ERDLLNR.R | | 59.88 | 914.4933 | 2 | 2 |  |
|  |  |  |  |  |  | |  |  |  |  |  |
|  |  |  |  |  |  | |  |  |  |  |  |
| 6 | JAC96608.1 | Snake venom metalloproteinase C, partial | 175.26 | 12 | K.cPITLYQcR.A | | 116.43 | 1209.564 | 2 | 0 | Ca(C1,C8) |
|  |  |  |  |  |  | |  |  |  |  |  |
|  |  |  |  |  | R.HcVDVTTAY | | 93.92 | 1064.46 | 2 | 0 | Ca(C2) |
| **PLA_2_ (1 protein)** | | | | | | | | | | | |
|  |  |  |  |  | |  |  |  |  |  |  |
| 1 | JAC96563.1 | Phospholipase A2 Group IIE, partial | 106.87 | 6 | | K.AAALcFR.H |  | 807.4061 | 2 | 0 | Ca(C5) |
|  |  |  |  |  | |  |  |  |  |  |  |
| **SVSP (2 proteins)** | | | | | | | | | | | |
| 1 | JAC96577.1 | Serine protease A, partial | 50.62 | 6 | | K.YFcLK.S | 50.62 | 729.352 | 2 | -0.3 | Ca(C3) |
|  |  |  |  |  | |  |  |  |  |  |  |
| 2 | JAC96575.1 | Serine protease C, partial | 105.85 | 8 | | R.ETYFcLPSR.N | 90.72 | 1171.533 | 2 | -0.8 | Ca(C5) |
|  |  |  |  |  | |  |  |  |  |  |  |
| **LAAO (1 protein)** | | | | | | | | | | | |
| 1 | JAC96580.1 | L-amino acid oxidase B variant 1 | 244.33 | 7 | | A.GHKVTVLEASER.V | 109.09 | 1324.71 | 2 | 1.7 |  |
|  |  |  |  |  | | G.WIDSTIK.S | 53.56 | 918.4811 | 2 | 0.6 |  |
|  |  |  |  |  | | E.FLEIAR.N | 35.63 | 747.4279 | 2 | 0.7 |  |
|  |  |  |  |  | | K.HDDIFAY.E | 35.22 | 879.3762 | 2 | 1.9 |  |
|  |  |  |  |  | |  |  |  |  |  |  |
| **Non-enzymatic proteins** | | | | | | | | | | | |
| **Snaclec (1 protein)** | | | | | | | | | | | |
| 1 | JAC96617.1 | C-type lectin J | 268.08 | 23 | | K.SSADYVWIGLWNK.R | 151.45 | 1537.757 | 2 | 2.7 |  |
|  |  |  |  |  | | R.cGDDYPFVcK.F | 126.6 | 1259.495 | 2 | 0.5 | Ca(C1,C9) |
|  |  |  |  |  | | R.TWFNLR.C | 67.03 | 835.4341 | 2 | 0.4 |  |
|  |  |  |  |  | | K.NcFGLEK.K | 62.81 | 866.3956 | 2 | 0.6 | Ca(C2) |
|  |  |  |  |  | |  |  |  |  |  |  |
| **CRISP (1 protein)** | | | | | | | | | | | |
|  |  |  |  |  | |  |  |  |  |  |  |
|  |  |  |  |  | |  |  |  |  |  |  |
| 1 | JAC96631.1 | Cysteine-Rich Secretory Protein B | 259.96 | 17 | | G.NVDFDSESPR.K | 102.49 | 1164.505 | 2 | 0.4 |  |
|  |  |  |  |  | | K.SNcAAScFcHSEIK | 89.74 | 1669.665 | 2 | -0.1 | Ca(C3,C7) |
|  |  |  |  |  | | R.KPEIQNEIIDLHNSLRR.S | 69.18 | 2074.128 | 4 | -0.1 |  |
| **NGF (1 protein)** | | | | | | | | | | | |
| 1 | JAC88974.1 | nerve growth factor a | 112.4 | 10 | | R.INTAcVcVISR.K |  | 1291.638 | 2 | 0.6 | Ca(C5,C7) |
|  |  |  |  |  | | K.HWNSYcTTTDTFVR.A | 45.5 | 1786.773 | 3 | 0.2 | Ca(C6) |
| **VEGF (1 protein)** | | | | | | | | | | | |
| 1 | JAC96562.1 | Vascular endothelial growth factor F | 46.08 | 7 | | K.RNRSPEEGER.E | 32.18 | 1228.591 | 2 | -1.9 |  |
